# Total and specific potato intake and risk of type 2 diabetes: results from three US cohort studies and a substitution meta-analysis of prospective cohorts

**DOI:** 10.1136/bmj-2024-082121

**Published:** 2025-08-06

**Authors:** Seyed Mohammad Mousavi, Xiao Gu, Fumiaki Imamura, Hala B AlEssa, Orrin Devinsky, Qi Sun, Frank B Hu, JoAnn E Manson, Eric B Rimm, Nita G Forouhi, Walter C Willett

**Affiliations:** 1 Department of Nutrition, Harvard T.H. Chan School of Public Health, Boston, MA 02115, USA; 2 MRC Epidemiology Unit, University of Cambridge School of Clinical Medicine, Cambridge, UK; 3 Department of Public Health Practice, College of Public Health, Kuwait University, Kuwait, Kuwait; 4 Department of Neurology, New York University Grossman School of Medicine, New York, NY, USA; 5 Channing Division of Network Medicine, Department of Medicine, Brigham and Women’s Hospital, Boston, MA, USA; 6 Department of Epidemiology, Harvard T.H. Chan School of Public Health, Boston, MA, USA; 7 Division of Preventive Medicine, Department of Medicine, Brigham and Women’s Hospital and Harvard Medical School, Boston, MA, USA

## Abstract

**Objectives:**

To investigate the associations between total and individual potato intake and risk of type 2 diabetes (T2D), estimate the effect on T2D risk of replacing potatoes with whole grains and other major carbohydrate sources, and conduct a dose-response and substitution meta-analysis of prospective cohort studies.

**Design:**

Prospective cohort study and dose-response meta-analysis of prospective cohort studies.

**Setting:**

Individual participant data from Nurses’ Health Study (1984-2020), Nurses’ Health Study II (1991-2021), and Health Professionals Follow-up Study (1986-2018).

**Participants:**

205 107 men and women free of diabetes, cardiovascular disease, or cancer at baseline.

**Main outcome measure:**

Incident type 2 diabetes.

**Results:**

During 5 175 501 person years of follow-up, T2D was documented in 22 299 participants. After adjustment for updated body mass index and other diabetes related risk factors, higher intakes of total potatoes and French fries were associated with increased risk of T2D. For every increment of three servings weekly of total potato, the rate for T2D increased by 5% (hazard ratio 1.05, 95% confidence interval (CI) 1.02 to 1.08) and for every increment of three servings weekly of French fries the rate increased by 20% (1.20, 1.12 to 1.28). Intake of combined baked, boiled, or mashed potatoes was not significantly associated with T2D risk (pooled hazard ratio 1.01, 95% CI 0.98 to 1.05). In substitution analyses, replacing three servings weekly of potatoes with whole grains was estimated to lower T2D rates by 8% (95% CI 5% to 11%) for total potatoes, 4% (1% to 8%) for baked, boiled, or mashed potatoes, and 19% (14% to 25%) for French fries. In contrast, replacing total potatoes or baked, boiled, or mashed potatoes with white rice was associated with an increased risk of T2D. In a meta-analysis of 13 cohorts (587 081 participants and 43 471 diagnoses of T2D), the pooled hazard ratio for risk of T2D with each increment of three servings weekly of total potato was 1.03 (95% CI 1.02 to 1.05) and of fried potatoes was 1.16 (1.09 to 1.23). In substitution meta-analyses, replacing three servings weekly of total, non-fried, and fried potatoes with whole grains was estimated to lower the risk of T2D by 7% (95% CI 5% to 9%), 5% (3% to 7%), and 17% (12% to 22%), respectively.

**Conclusions:**

Higher intake of French fries, but not combined baked, boiled, or mashed potatoes, was associated with a higher risk of T2D. The T2D risk linked to potato intake seemed to depend on the food being replaced: replacing potato with whole grains was associated with lower risk, whereas replacing with white rice was associated with increased risk.

## Introduction

Potatoes, the third most commonly consumed food crop and the main non-cereal food, contribute a substantial quantity of daily energy.^1^ Several US guidelines classify potatoes as vegetables.^2^ ^3^ Although potatoes contain various nutrients such as fiber, vitamin C, potassium, polyphenols, and magnesium, previous research has raised caution about their effects on health.^4^ The high starch content of potatoes, leading to a high glycemic index and load, combined with possible loss of nutrients and possible health risks resulting from various cooking methods, could contribute to adverse health outcomes.^5^ ^6^ Although the health benefits of most vegetables are widely acknowledged, the association between potato consumption and health outcomes, particularly type 2 diabetes (T2D), remains a subject of debate.^7^

The results of previous prospective studies investigating the association between potato consumption and T2D were inconclusive. Whereas some studies have indicated a positive association,^8^ ^9^ others found no statistically significant association,^10^ ^11^ ^12^ ^13^ and some even observed an inverse association.^14^ Similar inconsistencies have been found in meta-analyses evaluating these relations.^15^ ^16^ A recent individual participant data (IPD) meta-analysis^17^ of seven US cohorts found no association with total potato intake but a modest increased risk with fried potato intake. That study did not, however, assess dietary substitutions, which may have an effect on T2D risk. Few studies have estimated the effect on T2D risk of replacing potatoes with alternative carbohydrate sources; though some suggest benefits from replacing potatoes with whole grains and non-starchy vegetables.^8^ ^18^ Inconsistent findings may stem from regional differences in potato consumption, variations in methodology (including only baseline versus repeated dietary assessments), inadequate control for confounding variables, and disregard of the potential for reverse causation. Thus, long term observational studies with advanced methodologies and high quality data are needed to clarify these associations. Additionally, substitution analyses are particularly important as the effect of potato consumption might depend on the replacement sources of energy; in contrast with potatoes, whole grains are consistently linked to lower risks of many adverse health outcomes.^19^

The extensive data from the Nurses’ Health Study (NHS), Nurses’ Health Study II (NHSII), and Health Professionals Follow-up Study (HPFS), encompassing almost four decades with many repeated assessments of diet and other variables, provide a more precise evaluation of potato and T2D associations than studies with a single, baseline only assessment. Our previous analyses of data from the US health professional cohorts indicated a robust positive association between high potato consumption—particularly French fries—and increased incidence of T2D.^18^ In the current study, we examined the same three cohorts, including more than 7000 additional people with T2D documented, with extended follow-up, and evaluated the extent to which having only baseline diet assessments, a common limitation in most epidemiological studies, might attenuate associations. Furthermore, we assessed the impact of replacing potatoes with other frequently consumed foods, and explored latency periods along with the possibility of reverse causation. Finally, to inform dietary guidelines, we also conducted an updated dose-response meta-analysis to evaluate the relation between total and specific potato intake and T2D risk and compare the associations of potatoes and whole grains with T2D risk.

## Methods

### Cohort analyses

#### Study population and design

This analysis involved participants from three ongoing longitudinal cohort studies: NHS, NHSII, and HPFS. NHS was initiated in 1976 and comprised a sample of 121 700 female registered nurses aged 30-55 years from 11 US states. NHSII, which started in 1989, included 116 429 female nurses aged 25-42 years from 14 states. HPFS was established in 1986 and included 51 529 male US health professionals aged 40-75 years. Participants in these cohorts were surveyed biennially using questionnaires that collected information on disease diagnoses, risk factors, medication use, and lifestyle factors. Dietary data were collected through a validated food frequency questionnaire every 2-4 years. The study’s baseline was set in 1984 for NHS, 1986 for HPFS, and 1991 for NHSII. Detailed documentation on the design and methods of these cohorts is provided elsewhere.^20^ ^21^ Participants who completed the baseline food frequency questionnaire (NHS 1984, n=81 702; NHSII 1991, n=95 221; HPFS 1986, n=51 530) and had no previous diagnosis of cancer, myocardial infarction, angina, stroke, coronary artery bypass grafting, or T2D were included. We excluded participants with incomplete baseline information on age or potato intake, with implausible reported energy intake (<500 or >3500 kcal/day (1 kcal=4.18 kJ=0.00418 MJ) for women, and <800 or >4200 kcal/day for men), who died at or before baseline, and who only completed the baseline survey. After exclusions, a total of 205 107 participants including 72 712 women from NHS, 90 232 women from NHSII, and 42 163 men from HPFS were included in the final analysis (see supplementary figure 1 for participant flow chart).

#### Assessment of dietary intake

Diet was assessed using validated semiquantitative food frequency questionnaires on usual dietary intake over the previous year. Dietary intakes in NHS were first collected in 1980 using a short questionnaire that was expanded and used in 1984, 1986, and then every four years to 2010. In both NHSII and HPFS, dietary intakes were evaluated every four years, starting in 1991 and 1986, respectively. In all food frequency questionnaires, participants were asked how often they consumed each food item, with a standard portion size provided. Nine response options were available, ranging from “never, or <1 time/month” to “≥6 times/day.” Three questions on potato consumption were asked: one about baked, boiled, or mashed potatoes (one medium or one cup), another about French fries (4-6 oz or one serving), and one about potato or corn chips (small bag or 1 oz). We adjusted the grams per serving overtime by monitoring weights in NHANES (National Health and Nutrition Examination Survey), allowing us to account for temporal variations in portion sizes for specific foods (see supplementary table 1). Total potato intake was calculated by summing the servings of baked, boiled, or mashed potatoes and French fries. We did not include consumption of chips (referred to as crisps in the UK) in the total potato intake as the food frequency questionnaire combined potato and corn chips in a single question, and therefore we treated chips as a separate item. Supplementary table 2 provides information on other food groups, such as total red meat, fish, dairy, nuts and legumes, poultry, eggs, fruits, vegetables, and sugar sweetened beverages. We calculated intakes of total energy and alcohol using data from the Harvard University Food Composition Database. The validity and reproducibility of the food frequency questionnaire have been evaluated through dietary records in a subset of 649 male participants in HPFS and 736 female participants in NHS and NHSII.^22^ The deattenuated Pearson correlations between total potato intake reported on the food frequency questionnaires and seven day dietary records (as reference) were 0.61 for women and 0.63 for men.

#### Assessment of diabetes

Participants from each cohort self-reported newly diagnosed T2D through biennial questionnaires. These participants were mailed a detailed follow-up questionnaire to gather more information on symptoms, diagnostic tests, and use of hypoglycemic drugs. A T2D diagnosis was considered confirmed if participants fulfilled one or more of the American Diabetes Association’s criteria listed on the supplementary questionnaire: the presence of one or more classic symptoms (eg, excessive thirst, polyuria, weight loss, hunger, pruritus, or coma) along with a fasting plasma glucose level of ≥7.0 mmol/L or a random plasma glucose level of ≥11.1 mmol/L; in the absence of symptoms, at least two separate instances of raised plasma glucose levels, including fasting levels of ≥7.8 mmol/L, random plasma glucose levels of ≥11.1 mmol/L, or a plasma glucose level of ≥11.1 mmol/L during an oral glucose tolerance test; or using hypoglycemic medications such as insulin or oral diabetes drugs. Before 1998, people with T2D were identified based on the National Diabetes Data Group criteria,^23^ which defined diabetes as a fasting plasma glucose level of ≥7.8 mmol/L.

#### Assessment of covariates

We collected and updated data on a variety of risk factors and potential confounders for the association between potato consumption and risk of T2D, including age, race/ethnicity, family history of T2D, body weight, smoking status, physical activity, use of multivitamins, use of antihypertensives, use of cholesterol lowering drugs, and history of hypertension. This information was obtained through the primary biennial questionnaires. Additionally, we collected data on menopausal status and postmenopausal hormone use for women. Alcohol intake was assessed through food frequency questionnaires. The reliability and reproducibility of self-reported information on body weight, physical activity, and alcohol consumption have been documented elsewhere.^24^ ^25^ Body mass index (BMI) was calculated by dividing weight in kilograms by height in meters squared. Physical activity was quantified by assigning a metabolic equivalent of task (MET) value to each activity and multiplying it by the time spent on that activity weekly (MET-h/week). From the food frequency questionnaires, we calculated the modified Alternate Healthy Eating Index (AHEI) score after excluding trans fatty acid and polyunsaturated fat components owing to their presence in French fries and potato chips. Additionally, we used a geo-coded composite score, encompassing educational background, income, property value, and marital status, to represent the neighborhood socioeconomic status of each participant area level.^26^

### Statistical analysis

Person years of follow-up were determined by calculating the period from the date of returning the baseline food frequency questionnaire (1984 for NHS, 1991 for NHSII, and 1986 for HPFS) until the date of T2D diagnosis, death, loss to follow-up, or the end of follow-up (30 June 2020 for NHS, 30 June 2021 for NHSII, and 31 January 2018 for HPFS), whichever came first.

To assess the associations between various forms of potato consumption (total; baked, boiled, or mashed; French fries; and chips) and incidence of T2D, we used cohort specific Cox proportional hazards models to compute hazard ratios and corresponding 95% confidence intervals (CIs). In our main analysis, we calculated cumulative averages of dietary intakes from the baseline food frequency questionnaire to the beginning of each subsequent four year follow-up interval.^27^ This approach was taken to minimize the impact of random measurement errors resulting from within person variations and to reflect any changes in diet over time. For example, in NHS, we used the average potato consumption from 1984, 1986, 1990, and 1994 to estimate T2D risk from 1994 to 1998. Potato consumption was categorized based on the frequency of servings: total potato intake: <1/week (reference), 1/week, 2-4/week, 5-6/week, and ≥7/week, and specific potato types: almost never (reference), 1-3/month, 1/week, 2-4/week, and ≥5/week. To maintain an adequate sample size, we combined the two lowest consumption categories for baked, boiled, or mashed potatoes into a single reference category. Additionally, potato intake was assessed on a continuous scale, with increments of three servings weekly. We determined the model covariates using the modified disjunctive cause criterion,^28^ which was informed by a thorough review of the relevant literature. All analyses were stratified by age (months) as the time scale and calendar time (two year intervals) to control for confounding by age and account for secular trends. In the first multivariable model, we adjusted for total energy intake. The second model included further adjustments for race/ethnicity, smoking status, alcohol consumption, physical activity, multivitamin use, menopausal status and hormone use (NHS and NHSII only), family history of diabetes, use of antihypertensives, use of cholesterol lowering drugs, baseline hypertension history, BMI, and socioeconomic status. The third model included further adjustments for various food groups, including total red meat, poultry, fish, eggs, dairy products, nuts and legumes, fruits, vegetables, whole and refined grains, and sugar sweetened beverages, and was mutually adjusted for the various types of potatoes. Most covariates except for race, family history of diabetes, and baseline hypertension were updated biennially, whereas dietary variables and physical activity were updated every four years. The overall proportion of missing data on covariates was low across the three cohorts (eg, 10% on average for dietary variables). We handled missing values after baseline using a last observation carried forward approach, which is appropriate given the stability of covariates over short intervals in these cohorts.^29^ For residual missingness (see supplementary table 3), continuous variables with <0.5% missing data were imputed using cohort specific medians, whereas categorical variables (eg, race, smoking status, menopausal status, and hormone use) were handled using the missing indicator method. In previous analyses conducted within our cohorts, the missing indicator method generated results that were largely consistent with those obtained using the multiple imputation method.^30^

In the categorical analysis, we evaluated linear trends across categories by treating the median intake of each category as a continuous variable, with significance tested using the Wald test. To assess the potential dose-response relation between potato intake and risk of T2D, we pooled individual level data from the three cohorts and harmonized covariates to ensure consistent definitions and measurement across studies. We then applied a restricted cubic spline regression with three knots placed at the 10th, 50th, and 90th centiles of potato intake, using the SAS Macro %LGTPHCURV9.^31^ The model was stratified by cohort to allow for cohort specific baseline hazard functions, whereas covariate effects were assumed to be common across cohorts given the harmonized data collection methods and standardized definitions for covariates. To evaluate the robustness of this approach, we conducted sensitivity analyses including cohort-covariate interaction terms and cohort specific spline models. The presence of non-linearity was evaluated using a likelihood ratio test, comparing a model with a linear term to one including both linear and cubic spline terms. To assess the proportional hazards assumption in our Cox regression model, we introduced interaction terms between age (years) and potato intake (three servings weekly) or each of all confounding variables (see supplementary tables 4-6).

We also conducted several sensitivity analyses to determine the robustness of our findings. First, we explored whether the association between potato and T2D risk differed across various subgroups such as age, diet quality (assessed by modified AHEI score), physical activity levels, BMI, sex, history of hypertension at baseline, smoking status, and race/ethnicity. The interaction between potato consumption and dichotomous stratification factors was examined using the Wald test with one degree of freedom. We used likelihood ratio tests to evaluate the interaction of strata with multiple levels, such as smoking status and racial/ethnic groups, by comparing models with and without their product terms for potato consumption. Additionally, for variables treated as continuous (eg, BMI, physical activity, modified AHEI scores), we included interaction terms between potato consumption and the continuous form of these variables in the models and reported the corresponding P values for interaction. Second, we modeled dietary data in four additional ways: using only the baseline food frequency questionnaire, cumulative average intake while excluding the last three food frequency questionnaires before T2D diagnosis, the average of the most recent three food frequency questionnaires at the start of a two year follow-up period, and simple updated potato consumption over the follow-up period. Third, we repeated the main analyses adjusting for the modified AHEI,^32^ instead of individual food groups. Fourth, the main analysis was repeated, focusing on people with T2D symptoms, ascertained through reports of at least one diabetes related symptom in the supplementary questionnaire. This approach was taken because individuals at higher risk for diabetes tend to undergo more frequent screenings and may receive a diagnosis earlier, which can lead to surveillance bias. Fifth, to assess the potential for confounding by the diagnosis of disease endpoints during the follow-up period, we stopped updating the cumulative average dietary intakes of participants once they self-reported angina, myocardial infarction, or a coronary artery bypass graft procedure. Sixth, the analysis was adjusted for baseline BMI rather than time varying BMI. For intake of French fries, we further adjusted for BMI and BMI^2^ instead of using categorical BMI to account for both linear and non-linear effects. We also adjusted for confectionery intake, trans fatty acid intake, and the polygenic risk score for T2D.^33^ Seventh, to assess the extent to which time varying BMI mediates the associations, we excluded BMI from the main analysis and estimated the percentage of the associations that was mediated by BMI. Eighth, we examined the relation between every increment of three servings weekly for potato intake and T2D risk over intervals of 0-4, 5-8, 9-12, 13-16, 17-20, and 21-24 years to identify any potential bias from reverse causation through latency analysis. For instance, in NHSII, for a presumed latency period of 8-12 years, potato intake in 1991 was used to predict risk of T2D from 1999 to 2003, 1995 was used to predict risk of T2D from 2003 to 2007, and so on. We additionally excluded individuals with incident T2D diagnosed within the first 10 years of follow-up to assess potential bias from latent undetected diabetes. Finally, for predictors that showed non-proportional hazards, we modified our primary cohort risk models by including interaction terms between these covariates and a log-transformed age variable. In making this adjustment we aimed to account for potential time dependent effects and to examine whether these altered the primary estimates of the association between potato intake and T2D risk.

In modeled substitution analyses, for estimating risk of T2D associated with replacing three servings weekly of potatoes with common alternatives (whole grains, refined grains, starchy and non-starchy vegetables, legumes, white and brown rice), we applied the serving focused strategy instead of an energy focused one. We included both consumption of potatoes and replacement foods as continuous variables in the Cox proportional hazard models^34^ ^35^ and calculated hazard ratios and 95% CIs for the incidence of T2D, based on differences in the estimated coefficients along with their pooled variances. This approach compares the associations of specific foods with T2D risk, rather than assuming a direct causal effect of substitution. In a sensitivity analysis, we adjusted for energy from macronutrients instead of total energy to evaluate the robustness of the observed associations. We employed a two stage IPD meta-analysis approach, conducting all analyses, except those stratified by race/ethnicity, individually within each cohort. The resulting coefficients were subsequently combined using variance weighted fixed effect meta-analysis. Alternatively, as a secondary approach, we combined coefficients using random effects models. Because of limited numbers of people with T2D for race/ethnicity subgroups, we analyzed the association using combined data from all cohorts. All statistical analyses were performed using SAS for UNIX version 9.4 (SAS Institute, Cary, NC), with all P values calculated as two sided and a significance level set at α=0.05.

#### Dose-response and substitution meta-analysis of published cohorts

The methods section in the supplementary file provides a comprehensive overview of the linear and non-linear dose-response meta-analyses, as well as the subgroup and sensitivity analyses and the assessment of evidence quality. Briefly, our findings were reported following the preferred reporting items for systematic reviews and meta-analyses (PRISMA) guidelines,^36^ and the study protocol was pre-registered on PROSPERO (CRD42023448736). We carried out comprehensive searches in PubMed/Medline, ISI Web of Science, and Embase up to July 2024, using predetermined search terms, without imposing any restrictions (see supplementary table 7). We included prospective cohort studies that investigated the association between potato consumption, total and specific types according to cooking method, and risk of T2D in populations without pre-existing cardiovascular diseases, cancer, and T2D at baseline (see supplementary table 8). First, we conducted a linear dose-response meta-analysis,^37^ ^38^ which examined the risk of T2D in relation to an increase of three servings weekly of potato using an inverse variance weighted model. Second, we performed a one stage mixed effects meta-analysis to model a potential non-linear association between potato intake and T2D risk using restricted cubic splines with three knots at the 10th, 50th, and 90th centiles of the distribution analysis.^39^ Finally, using meta-analyzed results, we estimated the effect of substituting three servings weekly of potatoes, both total and specific types, with whole grain. The estimate for potatoes was derived from the current meta-analysis, whereas the estimate for whole grains was obtained by updating our previously published dose-response meta-analysis.^40^ We first calculated β coefficients (log hazard ratios) for every three servings weekly of whole grains and potato (ie, total, fried, and non-fried). Then, using the variances of these coefficients and their covariance, we estimated the 95% CIs for this difference. We then exponentiated the difference to determine the hazard ratio for each substitution.^34^ The covariance between two coefficients was estimated from the multivariable meta-analysis of NHSI, NHSII, and HPFS. This approach was assumed to be valid, given the substantial weight of our cohorts in both meta-analyses. Additionally, we conducted sensitivity analyses using two alternative assumptions about covariance: assuming independence between parameters (*r*=0) and then adjusting our analysis by accounting for a modest correlation between parameter estimates (*r*=±0.20). We also performed several sensitivity analyses to evaluate the robustness of our findings against potential sources of heterogeneity.

### Patient and public involvement

No participants were involved in formulating the research question, selecting outcome measures, or contributing to the study’s design and implementation. They were also not asked to advise on data interpretation or manuscript writing. This was primarily due to the lack of infrastructure, resources, funding, and time necessary to support public involvement in these aspects of the research process. However, participants provided feedback on our questionnaires during follow-up, which we incorporated when feasible.

## Results

### Analyses from three US cohorts

#### Participant characteristics

During 5 175 501 person years of follow-up, we documented 22 299 diagnoses of T2D, including 9625 in NHS (median 27.5 years), 8698 in NHSII (median 25.6 years), and 3976 in HPFS (median 23.4 years). Men and women who reported higher total potato consumption tended to be less active, be less likely to take supplements, and have a higher total energy intake (table 1). Their dietary intakes were also characterized by higher consumption of total red meat, eggs, dairy products, legumes, starchy vegetables, refined grains, sugar sweetened beverages, and overall lower diet quality, as indicated by their scores on the modified AHEI. In all three cohorts, the average frequency of consumption of baked, boiled, or mashed potatoes was considerably higher than that of French fries (see supplementary figure 2).

**Table 1:** Characteristics of participants according to cumulative total potato intake in NHS (n=72 712), NHSII (n=90 232), and HPFS (n=42 163). Values are mean (standard deviation) unless stated otherwise*

Characteristics	Cumulative total potato intake (servings/week)										
	NHS				NHSII				HPFS		
	<1 (n=8922)	2-4 (n=23 442)	≥7 (n=7683)		<1 (n=8872)	2-4 (n=31 968)	≥7 (n=8798)		<1 (n=4142)	2-4 (n=13 662)	≥7 (n=5662)
Age at baseline (years)	51.9 (6.8)	50.1 (7.2)	49.7 (7.3)		36.5 (4.6)	36.0 (4.6)	35.5 (4.7)		55.0 (9.4)	53.0 (9.5)	52.0 (9.6)
Body mass index	24.6 (4.2)	24.8 (4.4)	25.1 (4.8)		23.8 (4.5)	24.5 (5.1)	25.7 (6.1)		25.4 (3.3)	25.4 (3.3)	25.5 (3.2)
Physical activity (MET-h/week)	16.2 (22.4)	13.6 (18.4)	11.8 (19.8)		25.1 (32.0)	20.5 (27.0)	18.7 (26.1)		22.5 (26.6)	21.3 (24.9)	19.5 (23.8)
Alcohol intake (g/day)	7.0 (11.2)	7.1 (11.2)	6.4 (11.2)		3.1 (6.1)	3.2 (6.2)	2.9 (6.3)		10.7 (15.1)	11.2 (14.9)	11.9 (15.9)
Current smoking (cigarettes/day) (No (%)):											
1-14	729 (8)	1739 (7)	550 (7)		474 (5)	1797 (6)	491 (6)		98 (2)	321 (2)	152 (3)
15-24	718 (8)	2286 (10)	845 (11)		325 (4)	1437 (4)	537 (6)		137 (3)	399 (3)	215 (4)
>24	535 (6)	1559 (7)	653 (9)		144 (2)	620 (2)	268 (3)		88 (2)	347 (3)	215 (4)
Race/ethnicity (No (%)):											
White	8066 (90)	22 092 (94)	7312 (96)		7859 (89)	29 824 (93)	8316 (95)		3502 (84)	12 396 (91)	5279 (95)
Black	197 (2)	229 (1)	37 (0)		181 (2)	429 (1)	75 (1)		91 (2)	108 (1)	28 (0)
Hispanic	107 (1)	190 (1)	38 (0)		205 (2)	570 (2)	103 (1)		24 (1)	66 (0)	24 (0)
Asian	148 (2)	100 (0)	15 (0)		358 (4)	312 (1)	36 (0)		214 (5)	178 (1)	25 (0)
Family history of diabetes (No (%))	1749 (20)	4370 (19)	1471 (19)		3529 (40)	13 128 (41)	3893 (44)		919 (22)	3116 (23)	1303 (23)
History of hypertension (No (%))	1838 (21)	4605 (20)	1542 (20)		511 (6)	1841 (6)	669 (8)		842 (20)	2600 (19)	1063 (19)
Current supplement use (No (%))	3817 (43)	8822 (38)	2327 (30)		4266 (48)	14 041 (44)	3445 (39)		1978 (48)	5825 (43)	2045 (36)
Postmenopausal hormone use (No (%))	1431 (16)	3140 (13)	821 (11)		214 (2)	777 (2)	265 (3)		-	-	-
Dietary intake†:											
Total energy intake (kcal/d)	1400 (460)	1718 (477)	2147 (530)		1450 (478)	1737 (491)	2252 (541)		1620 (519)	1908 (547)	2482 (630)
Baked, boiled, or mashed potato	0.05 (0.03)	0.27 (0.14)	0.82 (0.35)		0.05 (0.03)	0.20 (0.11)	0.65 (0.29)		0.05 (0.03)	0.24 (0.14)	0.70 (0.41)
French fries	0.01 (0.01)	0.05 (0.05)	0.16 (0.17)		0.01 (0.02)	0.08 (0.06)	0.32 (0.23)		0.01 (0.02)	0.07 (0.06)	0.30 (0.25)
Potato or corn chips	0.05 (0.11)	0.11 (0.19)	0.18 (0.27)		0.07 (0.15)	0.15 (0.19)	0.27 (0.29)		0.06 (0.15)	0.13 (0.20)	0.22 (0.29)
Total red meat	0.77 (0.61)	1.10 (0.62)	1.56 (0.79)		0.53 (0.52)	0.90 (0.56)	1.47 (0.82)		0.78 (0.72)	1.04 (0.74)	1.67 (0.98)
Processed red meat	0.19 (0.28)	0.29 (0.30)	0.42 (0.39)		0.13 (0.22)	0.22 (0.24)	0.35 (0.35)		0.25 (0.37)	0.33 (0.38)	0.50 (0.50)
Unprocessed red meat	0.58 (0.47)	0.80 (0.49)	1.13 (0.61)		0.41 (0.41)	0.68 (0.44)	1.11 (0.64)		0.53 (0.52)	0.70 (0.52)	1.17 (0.71)
Poultry	0.55 (0.49)	0.51 (0.37)	0.48 (0.37)		0.65 (0.51)	0.67 (0.43)	0.75 (0.50)		0.55 (0.50)	0.56 (0.43)	0.62 (0.47)
Fish	0.29 (0.27)	0.27 (0.23)	0.24 (0.21)		0.24 (0.26)	0.23 (0.21)	0.24 (0.24)		0.34 (0.35)	0.33 (0.29)	0.33 (0.34)
Egg	0.21 (0.34)	0.34 (0.30)	0.39 (0.36)		0.13 (0.19)	0.17 (0.19)	0.23 (0.25)		0.28 (0.41)	0.31 (0.38)	0.43 (0.51)
Total dairy	1.87 (1.38)	2.00 (1.34)	2.09 (1.38)		2.15 (1.48)	2.26 (1.43)	2.36 (1.50)		1.75 (1.46)	1.90 (1.38)	2.14 (1.53)
Legumes	0.15 (0.18)	0.19 (0.17)	0.24 (0.21)		0.16 (0.22)	0.20 (0.20)	0.26 (0.27)		0.20 (0.23)	0.24 (0.23)	0.32 (0.31)
Nuts	0.20 (0.38)	0.21 (0.31)	0.23 (0.34)		0.13 (0.22)	0.15 (0.21)	0.18 (0.23)		0.33 (0.54)	0.36 (0.53)	0.41 (0.54)
Fruits	1.49 (1.21)	1.42 (1.05)	1.36 (1.07)		1.27 (1.07)	1.19 (0.93)	1.15 (0.97)		1.64 (1.55)	1.60 (1.30)	1.55 (1.29)
Vegetables	2.68 (1.72)	2.70 (1.41)	2.84 (1.55)		2.39 (1.80)	2.37 (1.50)	2.60 (1.70)		3.60 (2.35)	3.86 (2.12)	4.18 (2.22)
Non-starchy vegetables	2.52 (1.66)	2.49 (1.35)	2.58 (1.46)		2.33 (1.76)	2.31 (1.47)	2.54 (1.66)		3.72 (2.39)	4.00 (2.15)	4.38 (2.27)
Starchy vegetables	0.31 (0.31)	0.41 (0.29)	0.51 (0.36)		0.32 (0.33)	0.41 (0.33)	0.55 (0.43)		0.38 (0.38)	0.48 (0.37)	0.62 (0.49)
Brown rice	0.04 (0.13)	0.03 (0.08)	0.02 (0.06)		0.07 (0.16)	0.06 (0.11)	0.05 (0.13)		0.07 (0.20)	0.07 (0.13)	0.06 (0.15)
White rice	0.10 (0.19)	0.11 (0.14)	0.10 (0.12)		0.17 (0.34)	0.14 (0.19)	0.14 (0.18)		0.14 (0.32)	0.13 (0.20)	0.13 (0.17)
Sugar sweetened beverages	0.16 (0.48)	0.28 (0.54)	0.48 (0.76)		0.28 (0.65)	0.45 (0.82)	0.75 (1.11)		0.25 (0.55)	0.32 (0.56)	0.54 (0.76)
Whole grains	0.53 (0.58)	0.56 (0.52)	0.74 (0.51)		0.70 (0.68)	0.71 (0.56)	0.71 (0.59)		0.69 (0.77)	0.78 (0.71)	0.76 (0.76)
Refined grains	1.32 (0.80)	1.79 (0.81)	2.20 (0.93)		1.85 (1.04)	2.14 (0.93)	2.62 (1.06)		1.62 (1.00)	1.98 (0.96)	2.52 (1.15)
Modified AHEI score‡	42.3 (10.1)	37.2 (9.3)	31.2 (8.9)		42.5 (9.9)	36.9 (9.3)	31.0 (9.2)		43.0 (10.4)	41.0 (10.3)	36.0 (10.2)

AHEI=Alternate Healthy Eating Index; NHS=Nurses’ Health Study; NHSII=Nurses’ Health Study II; HPFS=Health Professionals’ Follow-up Study; MET-h=metabolic equivalent tasks per hour.

* Except for age, all variables have been standardized according to age distribution. The cumulative average overtime was used to represent all variables, except for age, family history of diabetes, and baseline hypertension.

† Dietary intakes are in servings per day unless stated otherwise.

‡ Calculated without including trans fatty acids and polyunsaturated fat because French fries and potato or corn chips were among major contributors to these components.

#### Potato intake and T2D risk

**Fig 1:**
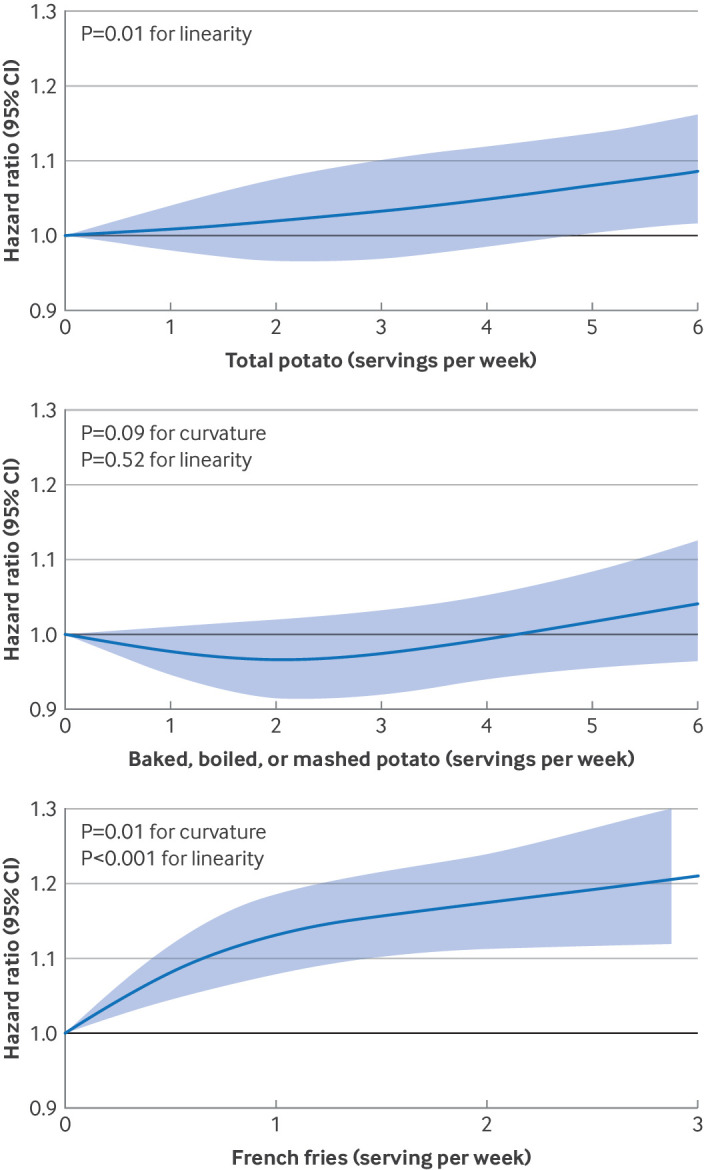
Dose-response relations between intake of total potato; baked, boiled, or mashed potatoes; and French fries and incidence of type 2 diabetes. Data were combined from three cohorts (Nurses’ Health Study, Nurses’ Health Study II, and Health Professionals Follow-up Study; total n=205 107). Associations were evaluated using restricted cubic spline models with three knots placed at the 10th, 50th, and 90th centiles, adjusting for multiple covariates. The model was stratified by age (months), cohort, and calendar time (two year intervals), and adjusted for total energy intake, race/ethnicity (white adults, non-white adults), smoking status (never, past, and current (cigarettes/day): 1-14, >15-24, >24), alcohol intake (0, 0-4.9, 5-9.9, 10-14.9, 15-29.9, and >30 g/day), physical activity (<3, 3.0-8.9, 9.0-17.9, 18.0-26.9, ≥27 MET-h/week), multivitamin use, menopausal status and hormone use (Nurses’ Health Study or Nurses’ Health Study II), family history of type 2 diabetes, antihypertensive use, cholesterol lowering drug use, history of hypertension, socioeconomic status, time varying body mass index (<21, 21.0-22.9, 23.0-24.9, 25.0-26.9, 27.0-29.9, 30.0-32.9, 33.0-34.9, 35-39.9, ≥40), and dietary covariates intakes (including total red meat, poultry, fish, egg, total dairy, nuts and legumes, fruits, vegetables, sugar sweetened beverages, whole grain, and refined grain, and mutual adjustment for baked, boiled or mashed potatoes and for French fries. MET-h=metabolic equivalent tasks per hour

In the pooled analysis of the three cohorts, adjusted for age and total energy intake, a strong association was found between total potato intake and higher risk of T2D. This association was substantially attenuated, yet remained positive, after further adjustments for demographic, lifestyle, and dietary factors (table 2). Participants with an intake of seven or more servings weekly of total potato had a 12% higher risk of T2D (pooled hazard ratio 1.12, 95% CI 1.02 to 1.24, P<0.001 for trend) compared to those with an intake of less than one serving weekly. An increment of three servings weekly for total potato was associated with a higher risk of T2D (hazard ratio 1.05, 95% CI 1.02 to 1.08). We found no evidence of departure from linearity (P=0.72 for curvature, fig 1).

**Table 2:** Associations between potato intakes and risk of diabetes in NHS (n=72 712), NHSII (n=90 232), and HPFS (n=42 163)

	Frequency of potato consumption (servings/week)					P for trend	Hazard ratio* (95% CI)
**Total potato**	**<1**	**1**	**2-4**	**5-6**	**≥7**		
No of participants with T2D/person years	788/270 016	2172/676 037	11 458/2 645 546	6705/1 338 773	1176/245 130		
Model 1	Ref	1.05 (0.97 to 1.14)	1.29 (1.19 to 1.38)	1.57 (1.45 to 1.69)	1.85 (1.68 to 2.03)	<0.001	1.30 (1.27 to 1.33)
Model 2	Ref	0.99 (0.91 to 1.08)	1.05 (0.98 to 1.13)	1.13 (1.05 to 1.22)	1.25 (1.14 to 1.38)	<0.001	1.10 (1.07 to 1.13)
Model 3	Ref	0.97 (0.90 to 1.06)	1.00 (0.92 to 1.07)	1.03 (0.95 to 1.11)	1.12 (1.02 to 1.24)	<0.001	1.05 (1.02 to 1.08)
**Baked, boiled, or mashed potatoes**	**<1**		**1**	**2-4**	**≥5**		
No of participants with T2D/person years	1917/594 299		4204/1 196 275	12 754/2 731 261	3462/653 665		
Model 1	Ref		1.05 (0.99 to 1.10)	1.16 (1.11 to 1.22)	1.25 (1.18 to 1.33)	<0.001	1.13 (1.10 to 1.16)
Model 2	Ref		1.00 (0.95 to 1.06)	1.01 (0.96 to 1.07)	1.06 (1.00 to 1.13)	0.008	1.05 (1.02 to 1.08)
Model 3	Ref		0.97 (0.92 to 1.02)	0.96 (0.91 to 1.01)	0.99 (0.93 to 1.05)	0.62	1.01 (0.98 to 1.05)
**French fries**	**Almost never**	**1-3/month**	**1**	**2-4**	**≥5**		
No of participants with T2D/person years	2952/985 067	10 803/2 542 718	5234/1 103 370	3130/517 430	180/26 919		
Model 1	Ref	1.40 (1.34 to 1.46)	1.77 (1.69 to 1.86)	2.26 (2.14 to 2.40)	3.17 (2.71 to 3.71)	<0.001	2.18 (2.09 to 2.28)
Model 2	Ref	1.14 (1.09 to 1.19)	1.22 (1.16 to 1.28)	1.29 (1.22 to 1.37)	1.44 (1.23 to 1.69)	<0.001	1.35 (1.27 to 1.44)
Model 3	Ref	1.09 (1.04 to 1.13)	1.12 (1.06 to 1.18)	1.15 (1.09 to 1.23)	1.27 (1.08 to 1.49)	<0.001	1.20 (1.12 to 1.28)
**Potato or corn chips**	**Almost never**	**1-3/month**	**1**	**2-4**	**≥5**		
No of participants with T2D/person years	2763/787 691	8361/1 945 784	4893/1 112 988	5435/1 137 550	847/191 488		
Model 1	Ref	1.12 (1.07 to 1.17)	1.22 (1.16 to 1.28)	1.23 (1.17 to 1.29)	1.23 (1.14 to 1.34)	<0.001	1.15 (1.11 to 1.19)
Model 2	Ref	1.02 (0.97 to 1.06)	1.05 (1.00 to 1.10)	1.04 (0.98 to 1.09)	1.07 (0.99 to 1.16)	0.01	1.07 (1.03 to 1.11)
Model 3	Ref	0.98 (0.94 to 1.03)	0.98 (0.93 to 1.03)	0.95 (0.90 to 1.00)	0.97 (0.89 to 1.06)	0.50	1.02 (0.98 to 1.06)

NHS=Nurses’ Health Study; NHSII=Nurses’ Health Study II; HPFS=Health Professionals Follow-up Study; MET-h=metabolic equivalent tasks per hour; T2D=type 2 diabetes.

Dietary intakes were cumulative averages from the baseline food frequency questionnaire to the start of each four year follow-up interval.

Model 1 was stratified by age (months) and calendar time (two year interval) and adjusted for total energy intake. Model 2 was additionally adjusted for race/ethnicity (white, non-white), smoking status (never, past, and current (cigarettes/day): 1-14, >15-24, >24), alcohol intake (0, 0-4.9, 5-9.9, 10-14.9, 15-29.9, >30 g/day), physical activity (<3, 3.0-8.9, 9.0-17.9, 18.0-26.9, ≥27 MET-h/week), multivitamin use (yes/no), menopausal status and hormone use (NHS or NHSII), family history of type 2 diabetes (yes/no), antihypertensive use (yes/no), cholesterol lowering drug use (yes/no), history of hypertension at baseline (yes/no), socioeconomic status, and time varying body mass index (<21, 21.0-22.9, 23.0-24.9, 25.0-26.9, 27.0-29.9, 30.0-32.9, 33.0-34.9, 35-39.9, ≥40). All covariates (except race/ethnicity, family history of diabetes, and baseline hypertension) were updated every two years. Model 3 was adjusted for the covariates in model 2+updated cumulative average intake of servings daily (fifths) of total red meat, poultry, fish, egg, total dairy, nuts and legumes, fruits, vegetables, sugar sweetened beverages, whole grains, and refined grains, and mutual adjustment for baked, boiled, or mashed potatoes and for French fries.

* For three servings weekly.

However, the risks associated with potato intake varied by cooking method. Consuming five or more servings weekly of French fries compared with almost never was associated with a 27% higher rate of T2D after multivariable adjustment (pooled hazard ratio 1.27, 95% CI 1.08 to 1.49, P<0.001 for trend), and an estimated 20% higher incidence of T2D (hazard ratio 1.20, 95% CI 1.12 to 1.28) for every three servings weekly greater intake. In the dose-response analysis, a linear association was observed, showing a steady increase in risk with higher intake of French fries (P<0.001 for linearity, fig 1). In contrast, after multivariable adjustment, no increase in T2D incidence was estimated for combined baked, boiled, or mashed potato intake, or for intake of potato or corn chips. The pooled hazard ratio for consuming five or more servings weekly of baked, boiled, or mashed potatoes compared with less than one serving weekly was 0.99 (95% CI 0.93 to 1.05, P=0.62 for trend). Similarly, the hazard ratio for five or more servings weekly of potato or corn chips compared with almost no intake weekly was 0.97 (95% CI 0.89 to 1.06, P=0.50 for trend). An increase of three servings weekly of baked, boiled, or mashed potatoes and potato or corn chips showed similar minimal changes in risk (pooled hazard ratio 1.01 (95% CI 0.98 to 1.05) and 1.02 (0.98 to 1.06), respectively). The dose-response relation remained essentially null (fig 1). The pooled associations between intake of total and specific types of potato and risk of T2D remained consistent in the random effects model (data not shown).

### Sensitivity and subgroup analyses

In sensitivity analyses, the test for the proportional hazards assumption for potato consumption was not statistically significant, and the associations remained consistent after accounting for covariate specific non-proportional hazards (see supplementary table 9). Results were also robust when adjusting for baseline (instead of time varying) BMI, using a modified AHEI instead of individual food groups, stopping dietary updates after diagnosis of a cardiac event, restricting the analysis to individuals with symptoms of diabetes, and excluding individuals with a diagnosis of T2D within the first 10 years of follow-up. The increased risk associated with intake of French fries also remained statistically significant even after further adjustment for trans fatty acid intake, confectionery intake, polygenic risk score for T2D, and adjustment for BMI and BMI^2^ instead of categorical BMI (see supplementary tables 10 and 11). In subgroup analyses, we observed statistically significant effect modification by BMI and race in the associations between potato intake and risk of T2D (see supplementary table 12). The positive association between total potato intake and T2D incidence was somewhat stronger among participants with higher BMI and among white participants. Statistically significant interactions were also observed for BMI with specific forms of potato, including French fries and combined baked, boiled, or mashed potatoes. Among white participants, an increase of three servings weekly of combined baked, boiled, or mashed potatoes was associated with a slightly higher risk of T2D. In contrast, age, sex, physical activity level, adherence to the modified AHEI, smoking status, and baseline hypertension did not statistically significantly modify the associations between total or specific potato intakes and T2D risk.

In our analysis using different approaches to assessing dietary data, the strongest association between total potato consumption and T2D risk was observed when considering the cumulative average intake over follow-up, whereas the weakest association was detected when the analysis was restricted to only the three most recent assessments or when the most recent assessments were excluded (see supplementary table 13). Regardless of the dietary assessment method used, intake of French fries was consistently associated with an increased risk of T2D, with the highest point estimate in analysis of cumulative average intake over time for every three servings weekly. However, the results for baked, boiled, or mashed potatoes and for potato or corn chips were less consistent across different methods of dietary assessment. For an intake of three servings weekly of baked, boiled, or mashed potato, the hazard ratio was 1.03 (95% CI 1.01 to 1.05) based on baseline dietary data. For the same intake of potato or corn chips, the hazard ratio was 1.04 (1.02 to 1.07) using a simple updated dietary assessment. We estimated that time varying BMI collectively mediated 50% of the association between intake of French fries and T2D incidence. When BMI was removed from the model, the hazard ratio for every three servings weekly of French fries was 1.60 (95% CI 1.36 to 1.88). However, BMI was not a statistically significant mediator of the association between intake of total or combined baked, boiled, or mashed potato and T2D incidence (data not shown). When we examined relations over varying latency periods (see supplementary table 14), the association of total potato intake with T2D incidence was strongest 12-20 years before diagnosis. Additionally, our analyses showed a positive association for intake of baked, boiled, or mashed potatoes assessed 12-15 or 16-20 years before T2D diagnosis. The positive association between French fries and T2D consistently remained statistically significant across all latency periods examined. Finally, we observed a significant interaction between calendar time and the association between intake of baked, boiled, or mashed potato and incidence of T2D (P=0.008 for interaction). Although this association appeared stronger in recent years, it remained statistically non-significant (see supplementary table 15). To evaluate the assumption of homogeneous covariate effects across cohorts within the non-linear model, we incorporated interaction terms between cohort and covariates in the model. These interactions were generally small in magnitude and not statistically significant, and their inclusion did not materially change the main effect estimates (see supplementary figure 3). To further assess potential heterogeneity, we conducted cohort specific spline analyses using the same knot placements. The resulting dose-response curves were consistent in shape and direction across cohorts, supporting the validity of the pooled spline model (see supplementary figure 4).

### Substitution scenarios of potatoes with carbohydrate rich sources

**Fig 2:**
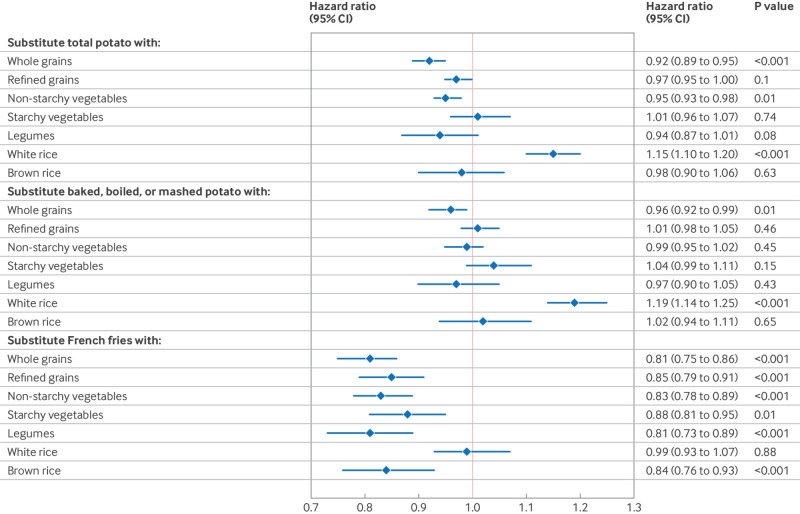
Associations between replacing potato intake with other foods and incidence of type 2 diabetes in the Nurses’ Health Study, Nurses’ Health Study II, and Health Professionals Follow-up Study (n=205 107). The effects of replacing three servings weekly of potatoes with alternative foods were assessed by simultaneously including both food intakes as continuous variables in the same multivariable Cox regression model. The model was stratified by age (months) and calendar time (two year intervals) and adjusted for race/ethnicity (white adults, non-white adults), smoking status (never, past, and current (cigarettes/day): 1-14, >15-24, >24), alcohol intake (0, 0-4.9, 5-9.9, 10-14.9, 15-29.9, >30 g/day), physical activity (<3, 3.0-8.9, 9.0-17.9, 18.0-26.9, ≥27 MET-h/week), multivitamin use, menopausal status and hormone use (Nurses’ Health Study or Nurses’ Health Study II), family history of type 2 diabetes, antihypertensive use, cholesterol lowering drug use, history of hypertension, socioeconomic status, time varying body mass index (<21, 21.0-22.9, 23.0-24.9, 25.0-26.9, 27.0-29.9, 30.0-32.9, 33.0-34.9, 35-39.9, ≥40), and dietary covariates intakes (including total red meat, poultry, fish, egg, total dairy, nuts and legumes, fruits, vegetables, sugar sweetened beverages, whole grain, and refined grain, and mutual adjustment for baked, boiled, or mashed potatoes and French fries) excluding the foods subject for substitutions. The models for brown rice and white rice did not include adjustments for whole grain and refined grain intakes, respectively. MET-h=metabolic equivalent tasks per hour

We estimated the modeled effects of substituting three servings weekly of major carbohydrate sources for potatoes (fig 2, supplementary table 16). Replacing three servings weekly of total potato with whole grains was estimated to lower the T2D rate by 8% (95% CI 5% to 11%). Substituting baked, boiled, or mashed potatoes with whole grains was estimated to lower incidence by 4% (1% to 8%), whereas replacing French fries lowered the rate by 19% (14% to 25%). Furthermore, replacing total potatoes with non-starchy vegetables, as well as replacing French fries with legumes, starchy and non-starchy vegetables, and even refined grains was estimated to lower the incidence of T2D. In contrast, replacing total and combined baked, boiled, or mashed potatoes with white rice was associated with a higher risk of T2D. When the analysis was limited to white participants only because Asian participants tended to consume more white rice, the estimates were attenuated but remained statistically significant (see supplementary table 17). In addition, adjusting energy from macronutrients instead of total energy did not alter the observed associations (data not shown). Supplementary tables 18-21 describe the results specific to each cohort.

### Meta-analysis of published cohorts

In our initial search of databases, we identified 503 papers relating potato intake to risk of T2D. After screening, 10 prospective studies (13 including three current cohorts) met the inclusion criteria for the final analysis (see supplementary figure 5 and table 22). Among them, five were from the US (39%), four from Europe (31%), two from Asia (15%), and two from Australia (15%), collectively involving 587 081 participants. Across these studies, 43 471 individuals with a diagnosis of T2D were reported during follow-up periods that ranged from four to 27 years. Three cohorts recruited only women, one cohort recruited only men, and the remaining nine cohorts recruited both men and women. In terms of dietary assessment methods, one study used food records, another employed food recall, and the remaining 11 used the food frequency questionnaire. Among these cohorts, five reassessed dietary intakes after baseline, whereas eight were limited to baseline assessments only. Notably, among the cohorts with repeated dietary assessments, only our three current cohorts included regular assessments over the follow-up period (see supplementary table 23). In terms of adjusting for confounders, seven cohorts accounted for primary confounders, whereas only four cohorts controlled for both primary and secondary confounders. Furthermore, three cohorts did not adjust for any secondary confounders (see supplementary table 24). Eight cohorts (62%) had a low risk of bias (Newcastle-Ottawa score ≥7). Using the ROBINS-E (Risk Of Bias In Non-randomized Studies - of Exposure) tool for assessment, 38% of the studies were judged as being at high risk of bias, mainly owing to the lack of adjustment for primary confounders (see supplementary tables 25 and 26).

Consistent with the findings from the pooled analysis of current cohorts, using the fixed effects model the summary hazard ratio of T2D associated with three servings weekly of total potato was 1.03 (95% CI 1.02 to 1.05; I^2^=57.3%, n=11), of fried potato was 1.16 (1.09 to 1.23; I^2^=50.9%, n=9), and of non-fried potato was 1.01 (0.99 to 1.03; I^2^=0.0%, n=6). Moreover, the summary hazard ratio for boiled potato was 1.01 (0.99 to 1.03; I^2^=62.5%, n=3) and for mashed potatoes was 1.06 (1.02 to 1.09; I^2^=50.1%, n=2) (see supplementary figures 6-10). Using the random effects model, the observed associations remained consistent in direction, with minor variations in magnitude. In the influence analysis, the pooled hazard ratio for each association of interest remained consistent when each study was sequentially excluded from the analysis (see supplementary figures 11-14). However, differences were observed in the relation between total potato intake and T2D risk across different geographic regions and dietary assessment methods (P<0.05 for interaction). A slightly higher risk for T2D was identified in studies from the US (hazard ratio 1.04, 1.01 to 1.07; I^2^=0.0%; n=5) and Europe (1.04, 1.02 to 1.06; I^2^=24.2%; n=2), as well as in cohorts utilizing repeated dietary assessments (1.04, 1.03 to 1.06; I^2^=0.0%; n=5) (see supplementary table 27). An interaction was identified between fried potato intake and variables such as geographic location, follow-up duration, study quality, participants’ sex, and dietary assessment method (P<0.05 for interaction). Specifically, we found stronger associations in US cohorts, those with more than 15 years of follow-up, cohorts consisting exclusively of women, studies with a low risk of bias, and studies utilizing a food frequency questionnaire for dietary assessment (see supplementary table 28). In contrast, the associations for intake of non-fried potato were consistent across all subgroups, with no statistically significant interactions (see supplementary table 29). In the non-linear dose-response analysis, evidence of deviation from linearity was detected for total potato (P=0.03 for non-linearity, n=11) and fried potato (P<0.001 for non-linearity, n=8) in relation to T2D incidence (see supplementary figure 15 and table 30). The risk of T2D increased by 12% with up to four servings weekly of fried potatoes, after which the risk plateaued, although data were sparse. Visual inspection of the funnel plot and the results of Egger’s regression test (P=0.13) did not indicate any evidence of publication bias on the association between total potato intake and T2D risk (see supplementary figure 16). The assessment of certainty of evidence using GRADE (Grading of Recommendations Assessment, Development and Evaluation) and NutriGrade provided a reasonable level of confidence in these findings (see supplementary tables 31 and 32). The associations of fried potato and total potato with T2D were graded as high and moderate based on GRADE, respectively. However, we found a low grade for non-fried and mashed potatoes followed by a very low grade for boiled potatoes. For NutriGrade, the association between all potato forms were determined to be moderate, except for boiled potato, which was low.

### Substitution scenarios from meta-analyses

**Fig 3:**
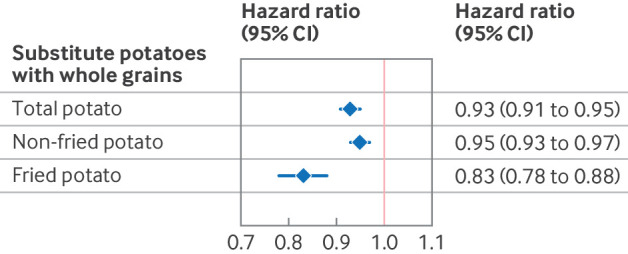
Estimated effects of replacing three servings weekly of different types of potatoes with whole grains on incidence of type 2 diabetes. Estimates are based on meta-analyzed data for potatoes and whole grains

For the substitution meta-analysis estimating the risk of T2D related to substitution of potatoes with whole grains, we updated the search date of our previously published study^40^ on whole grains and risk of T2D to include all available evidence. Eleven cohort studies investigated the relation between whole grain intake and T2D risk, with a total of 494 096 participants and 47 633 with a diagnosis of T2D, and included two additional cohorts beyond those in our earlier work^41^ ^42^ as well as four cohorts that overlapped with potato related meta-analysis. The summary hazard ratio for T2D for an increase of three servings weekly of whole grains was 0.96 (95% CI 0.96 to 0.97; I^2^=90.4%, supplementary figure 17). Replacing three servings weekly of total potatoes with whole grains was estimated to reduce the rate of T2D by 7% (95% CI 5% to 9%), with reductions of 5% (3% to 7%) for non-fried potatoes, and 17% (12% to 22%) for fried potatoes (fig 3). Confidence intervals changed minimally across sensitivity analyses with varying assumptions for covariance between intakes of whole grains and potato (see supplementary figure 18). Given the non-linear association observed between fried potato intake and risk of T2D, the substitution effects are most applicable at the intake level of three servings weekly, as derived from the linear dose-response meta-analysis, and they may not be consistent across different intake levels. Additionally, the substitution estimates remained consistent across various sensitivity analyses, including restricting to four overlapping cohorts, studies adjusting for energy, US based studies, and those employing repeated dietary assessments (see supplementary figure 19).

## Discussion

In three US cohorts comprising 205 107 men and women and 22 299 diagnoses of T2D, after adjusting for lifestyle and dietary factors related to diabetes risk, total potato intake was positively associated with a higher risk of T2D. This association was primarily driven by intake of French fries, whereas intake of baked, boiled, or mashed potatoes was not associated with T2D risk. When replacing any form of potatoes with whole grains, the incidence of T2D was estimated to be lower, with the strongest estimates for French fries. Our dose-response meta-analysis revealed similar associations between intake of total potato and intake of specific types of potato and risk of T2D. Additionally, the meta-analyzed estimates further reinforced the potential benefit of replacing potatoes with whole grains in lowering T2D risk.

### Comparison with other studies

We found positive associations between total potato intake and intake of French fries with T2D risk in both men and women, which is consistent with previous analyses of NHS, NHSII, and HPFS^18^ and with our meta-analysis of available data. Recent findings from prospective studies on the association between potato intake and T2D risk,^10^ ^11^ ^14^ ^43^ along with short term clinical trials that compared the metabolic effects of potato intake with other foods,^44^ ^45^ ^46^ have yielded inconclusive results. This finding has sparked an ongoing debate about whether potatoes, as a high starch staple food with high glycemic index and load, may influence T2D risk positively or negatively. This debate is further complicated by the choice of carbohydrate comparators, which are often considered in experimental studies but rarely assessed in long term cohort studies. In the Danish Diet, Cancer, and Health Cohort, the second largest study on this topic with 7695 participants with T2D over 16.3 years of follow-up, no significant association was observed between fried potato intake and risk of T2D after multivariable adjustment (hazard ratio 1.08, 95% CI 0.99 to 1.18).^11^ Additionally, a 2021 meta-analysis of eight prospective studies comparing high versus low potato intake reported no association between total potato intake and risk of T2D, though heterogeneity between studies was substantial.^15^ Conversely, a recent analysis of UK Biobank, which followed 174 665 participants for 11.4 years, found a 30% higher incidence of T2D among participants who consumed more than two servings daily of total potato compared with non-consumers.^8^ Similarly, a recent IPD meta-analysis of seven US cohorts reported consistent associations at intake levels comparable to those in our study (ie, >5 servings weekly versus no intake for total potatoes and >2 servings weekly versus no intake for French fries).^17^ However, our study found a higher risk of T2D at greater intake levels, as well as in our continuous analysis of an intake of three servings weekly, an aspect not examined in the recent IPD meta-analysis. Inconsistent findings could be attributed to multiple sources, such as the method of dietary assessment, which were often limited to baseline and regional differences in potato intake. The results of our subgroup meta-analysis support this, showing a statistically significant association between potato consumption and T2D risk in studies that conducted dietary assessments more than once after baseline, and within US and European cohorts. By using cumulative average intake (the mean of all individual measurements up to the start of each follow-up period), our results leverage all previous data to offer a more statistically robust analysis and reduce the effects of measurement errors compared with relying only on a baseline dietary assessment.^29^ Furthermore, our latency analysis suggested that the effect of potato consumption on diabetes risk is most notable 12-20 years before a T2D diagnosis; the weaker association in earlier follow-up could potentially represent reverse causation if participants knew they were in a prediabetic state. Global regional differences in potato intake may also contribute to the observed inconsistencies, with intake levels highest in Europe, followed by the US, and lowest in Asia and Africa.^47^ This global trend is also reflected in studies included in our meta-analysis: the Singapore Chinese Health Study reported a median intake of 4.6 g/day of potatoes,^43^ and the Tehran Lipid and Glucose Study reported a median intake of 22.4 g/day, with 60% of this from boiled instead of fried potatoes.^14^ These intakes are notably lower than the median intakes observed in our study (0.32 servings daily, or about 52 g/day) and in UK Biobank (0.64 servings daily).^8^ However, variation within populations is also substantial; for instance, in our cohorts, the median intake in the highest fifth was 0.90 servings daily, whereas it was only 0.07 servings daily in the lowest fifth, highlighting the wide range of potato consumption even within a single population. Our spline analysis indicated that the risk tended to increase at higher consumption levels.

Our findings are biologically plausible, although the exact mechanisms underlying the results are not fully understood. Potato ranks as a high glycemic index food owing to its rapidly absorbed starch content.^4^ Eating potatoes in large amounts can cause spikes in blood glucose and insulin levels, potentially resulting in oxidative stress to the pancreatic beta cells.^48^ This can initially lead to dysfunction or exhaustion of beta cells and may eventually contribute to insulin resistance, accompanied by raised levels of free fatty acids.^49^ Although glycemic index could explain some associations, it alone does not consistently predict T2D risk, and evidence from clinical trials has been inconclusive, showing mixed effects on cardiometabolic endpoints.^50^ ^51^ These complexities in glycemic response and metabolic impact could partly explain inconsistent associations for non-fried potatoes compared with the stronger relation seen with French fries, which not only have a high glycemic index but also contain added fats that have varied over time, salt, and potentially harmful products due to preparation at high temperatures. Deep frying potatoes results in the formation of harmful Maillard reaction products, including advanced glycation end products and heterocyclic amines.^52^ These compounds have been linked to adverse health outcomes, including an increased risk of T2D.^53^ Important changes in the composition of fat used for commercial production of French fries, which may increase the risk of T2D,^54^ have occurred over the follow-up of our cohorts. In the 1980s this fat was predominantly beef tallow, and in the early 1990s it shifted to partially hydrogenated plant oils with up to 30% *trans* isomers, which by 2010 was close to zero (unpublished data from our food composition analyses), and in 2018 the US Food and Drug Administration essentially banned partial hydrogenation.^55^ With additional follow-up we can further evaluate the effects of these changes on the relation between consumption of French fries and risk of T2D.

We found no statistically significant association between combined intake of baked, boiled, or mashed potatoes and risk of T2D after adjusting for other dietary variables. Our meta-analysis reinforces these findings, which similarly showed no evidence of an increased risk of T2D associated with non-fried potato intake. Consistent with our findings, an IPD meta-analysis reported no evidence of an association between intake of combined baked, boiled, or mashed potatoes and T2D risk after multivariable adjustment.^17^ Findings from a Danish cohort study likewise indicated no association between non-fried potato intake (ie, boiled, mashed, or roasted potatoes) and a higher risk of T2D after adjustment for dietary factors.^11^ Similarly, UK Biobank reported similar results for baked and boiled potatoes.^8^ However, both studies identified a positive association specifically for mashed potatoes. In our analysis, we were unable to distinguish mashed potatoes from baked or boiled potatoes, as these potatoes were grouped together in a single question in the food frequency questionnaire. Taken together, our findings suggest that the association between total potato consumption and T2D risk varies depending on the cooking method used, as different approaches can alter the nutritional profile of potatoes, potentially affecting glycemic index, energy content, and micronutrient levels.^56^ The effects of frying potatoes are likely to depend strongly on the type of fat used, and harmful compounds may be generated under some circumstances. These changes may account for the consistently strong association observed between intake of French fries and higher risk of T2D. As evidence on the associations of these potato forms is limited, future studies are needed to more accurately evaluate the individual contributions to T2D risk.

Because long term energy intake of individuals and populations is tightly regulated within narrow boundaries while maintaining body weight or physical activity levels, if intakes of specific foods are substantially changed, individuals will consciously or unconsciously change their intakes of other foods to compensate.^57^ Thus, analogous to the importance of the specific control group in a randomized trial, the association of potato consumption with risk of diabetes depends on the substituted foods; without a specified food, the implied comparison is the mix of other foods in a diet. In our substitution analysis, the greatest estimated risk reduction was seen when replacing any potato forms with whole grains, a finding further supported by the results of our meta-analysis. These findings are consistent with evidence indicating that food sources with a lower glycemic index and load and those rich in fiber, nutrients, and bioactive phytochemicals improve glycemic control and reduce inflammation and oxidative stress,^58^ and with substantial evidence that consumption of whole grains is inversely associated with risk of T2D.^59^ ^60^ Interestingly, whereas replacing potatoes with whole grains was associated with a lower risk of T2D, replacing potatoes with brown rice, despite its classification as a whole grain food, did not show a similar inverse association. This discrepancy highlights the distinct nutritional profile and dry matter content of individual whole grain foods, which could have an effect on their metabolic effects.^61^ For instance, brown rice contains substantially less fiber (about 3.5%) compared to whole grain wheat (about 10.7%), which serves as the primary whole grain source in this population.^62^ Furthermore, in our analysis, we found that the substitution of white rice for total as well as combined baked, boiled, or mashed potatoes was associated with a higher risk of T2D. This is consistent with the findings of another study, which reported lower postprandial insulin levels after potato compared with white rice consumption.^63^ Still, intake of rice, particularly brown rice, was relatively low in our population, highlighting the importance of caution in interpretation. Finally, in our analysis, substituting whole grains, legumes, starchy and non-starchy vegetables, and refined grains for French fries was associated with lower risk of T2D. Extending substitution analyses to meta-analyses could be an innovative step forward, formalizing the thought process used in dietary recommendations, such as prioritizing carbohydrate sources. Although our analysis was conducted under certain assumptions, ideally individual level data from all studies would be used. However, since such data are currently unavailable, future meta-analyses should consider incorporating individual level data**.**

### Strengths and limitations of this study

The current study has multiple strengths. First and foremost, repeated assessments of diet over three decades in three large cohorts allowed us to evaluate cumulative potato intake over time. This design enabled us to compare the effect of different dietary assessment methods, including the commonly used baseline only approach in most studies. Another strength was the substantial number of participants with a diagnosis of T2D and high follow-up rates within the prospective design, which ensured high statistical precision. Furthermore, we conducted a series of sensitivity and subgroup analyses to test the robustness of our results, along with a latency analysis, which is typically not feasible in randomized trials owing to shorter follow-up durations. Finally, combining the results of our cohort with existing evidence in the meta-analysis provided a more comprehensive view of how potato intake, various cooking methods, and alternative carbohydrate substitutions relate to T2D risk.

Although our data provided novel insights, our analysis has several limitations. First, owing to the inherent limitations of observational studies, our findings may still be influenced by residual or unmeasured confounding, despite adjusting for multiple lifestyle and dietary factors that could potentially affect the observed associations. Second, caution should be exercised when generalizing our results, as our study exclusively involved health professionals. However, owing to biological similarities across occupational groups, these findings are likely applicable to the general population. The relative homogeneity of educational and professional characteristics in our data is also a major advantage, as it enhances the quality of data and helps to minimize confounding. The consistency of our overall cohort findings with the meta-analysis also supports the generalizability of our more detailed analyses. Third, more than 90% of our study population was of European ancestry. By combining data from all three cohorts, we were able to obtain enough individuals with a diagnosis of T2D to evaluate associations. Interestingly, we observed some differences in associations among non‑Hispanic black, Asian (including east, southeast, and south Asian), and Hispanic participants, which may be related to their lower levels of potato consumption and distinct dietary patterns. More data from these populations would be beneficial. Fourth, our substitution analyses relied on statistical modeling that estimated associations using individual and population level data, rather than reflecting actual dietary replacements of potatoes with other foods over time. Therefore, the results should be interpreted considering other evidence.^64^ Ideally, future meta-analyses could incorporate individual level substitution analyses from all cohorts. Fifth, we did not differentiate between types of potatoes (eg, boiled, baked, or mashed) or account for variations in cooking methods, such as the use of butter, cream, or plant based alternatives, which may have mixed effects on T2D risk and warrant further investigation. Finally, we did not examine the effects of substituting sweet potatoes, which are rich in different carotenoids and phytochemicals, for white potatoes. Future studies are suggested to explore the health effects of replacing white potatoes with sweet potatoes.

### Policy implications and conclusions

In conclusion, the positive association of total potato intake and risk of T2D observed in our cohort and the dose-response meta-analysis was primarily driven by intake of French fries. Our substitution analysis highlights that the health effects of potatoes are strongly influenced by the food they replace. Replacing total potatoes, including baked, boiled, or mashed potatoes, or French fries with whole grains was associated with a lower risk of T2D, with the largest reduction observed when replacing with French fries. Replacing French fries with non-starchy and starchy vegetables, legumes, and even refined grains was also associated with lower risk of T2D. In contrast, replacing baked, boiled, or mashed potatoes or total potatoes with white rice was associated with a higher risk of T2D. Our findings underscore that the association between potato intake and T2D risk depends on the specific foods used as replacement. The findings also align with current dietary recommendations that promote the inclusion of whole grains as part of a healthy diet for the prevention of T2D.

**What is already known on this topic**

The association between potato intake, a high source of starch with a high glycemic index and load, and risk of type 2 diabetes (T2D) has been a subject of debate, especially in recent years
Few studies have estimated how substituting potatoes with other dietary choices might modify the risk of developing T2D

**What this study adds**

Findings from three major US cohorts, supported by an updated meta-analysis, suggest that the association between higher potato intake and increased T2D risk is primarily driven by intake of French fries
The health effect of potato intake on T2D risk varied based on the replacement food, with whole grains associated with a lower risk and white rice with an increased risk
Replacing any form of potatoes, particularly French fries, with whole grains is estimated to lower the risk of T2D, reinforcing the importance of promoting whole grains as an essential part of a healthy diet

## Data Availability

Owing to participant confidentiality and privacy considerations, access to the data are available upon written request. Following the standard controlled access protocol, all applications to utilize the Nurses’ Health Study, Nurses’ Health Study II (NHSII), or Health Professionals Follow-up Study (HPFS) resources undergo review by the External Collaborators Committee. This review process assesses the scientific objectives, ensures the appropriateness of the data for the proposed methodology, and verifies compliance with the Ethics and Governance Framework as well as the consent provided by participants. Researchers interested in accessing NHS/NHSII/HPFS data must submit a brief project proposal (contact nhsaccess@channing.harvard.edu). An initial response can typically be expected within four weeks of submission. Detailed information can be found at https://nurseshealthstudy.org/researchers. The primary analysis code utilized in this study is accessible on GitHub at: https://github.com/smmousavi1993-bot/potato_t2d .

## Web extra

Extra material supplied by authors

Supplementary information: Supplementary methods, tables 1-32, figures 1-19, and references

## Notes

Contributors: WCW conceptualized the study. SMM and FI did the systematic review and meta-analysis. SMM and XG analyzed and interpreted the cohorts’ data. QS, HA, OD, NGF, ER, JM, and FBH provided statistical advice and contributed to revision of the manuscript. SMM wrote the initial draft of the paper. SMM and WCW are the study’s guarantors, having full access to all data included in the study and taking responsibility for its integrity and the accuracy of the analysis. All authors involved have carefully reviewed and given their approval for the final draft of the manuscript. The corresponding author attests that all listed authors meet authorship criteria and that no others meeting the criteria have been omitted.

Transparency: The lead author (SMM) affirms that the manuscript is an honest, accurate, and transparent account of the study being reported; no important aspects of the study have been omitted. Any discrepancies from the study as planned have been disclosed.

Dissemination to participants and related patient and public communities: The findings will be shared with all study participants through our department’s public facing website, the Nutrition Source (https://nutritionsource.hsph.harvard.edu), a widely recognized and highly visited resource for information on evidence based nutrition. Additionally, our institutional media services will provide press releases regarding findings of public interest, such as the topic of this manuscript, and our researchers are frequently interviewed by journalists representing many forms of media. To further enhance impact and engagement, the findings will be presented at national and international scientific meetings and conferences, reaching researchers, clinicians, and policy makers.

Provenance and peer review: Not commissioned; externally peer reviewed.
