# Supplementary material for: Total and specific potato intake and risk of type 2 diabetes: results from three US cohort studies and a substitution meta-analysis of prospective cohorts
Source: BMJ. 2025 Aug 6;390:e082121. doi: 10.1136/bmj-2024-082121 (PMC12326520; doi:10.1136/bmj-2024-082121)
Supplement: Supplementary file 1 — Supplementary information: Supplementary methods, tables 1-32, figures 1-19, and references [file mous082121.ww1.pdf]

Supplementary Material

Total and specific potato intake and risk of type 2 diabetes: Results from three US cohort studies and a substitution meta-analysis of prospective cohorts

Supplementary Methods
Supplementary Table 1. Gram weight for 1 serving of different potatoes over time in NHS, NHS II, and HPFS
Supplementary Table 2. Components of food groups
Supplementary Table 3. The percentage of missingness in exposures and covariates in our cohorts
Supplementary Table 4. Assessment of the proportional hazards assumption for the primary exposure (total potato) and all the predictors in the multivariable-adjusted models assessing T2D risk
Supplementary Table 5. Assessment of the proportional hazards assumption for the primary exposure (baked, boiled, mashed potato) and all the predictors in the multivariable-adjusted models assessing T2D risk
Supplementary Table 6. Assessment of the proportional hazards assumption for the primary exposure (French fries) and all the predictors in the multivariable-adjusted models assessing T2D risk
Supplementary Table 7. Systematic review search strategy
Supplementary Table 8. Eligibility criteria by the PICOS statement
Supplementary Table 9. Hazard ratios and 95% confidence intervals for T2D associated with potatoes intake (3 servings/week) in the NHS (n=72,712), NHS II (n=90,232), and HPFS (n=42,163); models with covariate-specific non-proportional hazards
Supplementary Table 10. Associations between potato intakes and risk of diabetes in the NHS, NHS II, and HPFS adjusting for baseline BMI.
Supplementary Table 11. Sensitivity analyses of risk of type 2 diabetes incidence for potato consumption in NHS, NHS II, and HPFS.
Supplementary Table 12. Associations between 3-serving/week potato intake and risk of diabetes in the NHS, NHS II, and HPFS, stratified by key variables.
Supplementary Table 13. Associations between potato intakes and risk of diabetes in the NHS, NHS II, and HPFS using different types of dietary assessments.
Supplementary Table 14. Associations between every 3-serving/week increment in potato intakes and risk of diabetes in the NHS, NHS II, and HPFS by latency period.
Supplementary Table15. Associations between every 3 servings/week potatoes intake and risk of diabetes (NHS, NHSII, HPFS) in different time periods.
Supplementary Table 16. Associations between substituting 3-serving/week of other foods for 3-serving/week of potatoes intakes and the risk of type 2 diabetes in the NHS, NHS II, and HPFS
Supplementary Table 17. Associations between substituting 3-serving/week of white rice for 3-serving/week of potatoes intakes and the risk of type 2 diabetes in the NHS, NHS II, and HPFS, limiting the analysis to white participants only
Supplementary Table 18. Associations between potato intake and risk of diabetes in the NHS, NHS II, and HPFS (Cohort-specific results)
Supplementary Table 19. Associations between 3-serving/week potato intake and risk of diabetes in the NHS, NHS II, and HPFS, stratified by key variables (Cohort-specific results).
Supplementary Table 20. Associations between every 3-serving/week increment in potato intakes and risk of diabetes in the NHS, NHS II, and HPFS by latency period (cohort-specific results)
Supplementary Table 21. Associations between substituting 3-serving/week of other foods for three-serving/week of potatoes intakes and the risk of type 2 diabetes in the NHS, NHS II, and HPFS (Cohort-specific results)
Supplementary Table 22. List of excluded studies and exclusion reason
Supplementary Table 23. Characteristics of studies included in the meta-analysis.
Supplementary table 24. List of confounders among studies included in the meta-analysis.
Supplementary Table 25. Assessment of risk of bias with the Newcastle-Ottawa Scale.
Supplementary table 26. ROBINS-I judgement for each domain and overall
Supplementary table 27. Pre-specified subgroup meta-analyses of the association between every 3-serving/week total potato intake and risk of type 2 diabetes.

Supplementary table 28. Pre-specified subgroup meta-analyses of the association between every 3-serving/week fried potato intake and risk of type 2 diabetes.
Supplementary table 29. Pre-specified subgroup meta-analyses of the association between every 3-serving/week non-fried potato intake and risk of type 2 diabetes.
Supplementary Table 30. Summary hazard ratios from nonlinear dose-response analysis of potato consumption and risk of T2D
Supplementary Table 31. GRADE evidence table for the association of potatoes intake with risk of T2D.
Supplementary Table 32. NutriGrade Scoring system for the association of potatoes intake with risk of T2.

Supplementary Figure 1. Flowchart of study participants
Supplementary Figure 2. Trends in mean A) total potato, B) baked, boiled, mashed potato, C) French fries intake in the NHS, NHS II, and HPFS over time. (Mean potatoes calculated for each questionnaire returned)
Supplementary Figure 3. Dose–response relationships between total potato intake, baked/boiled/mashed potatoes, and French fries and the incidence of type 2 diabetes. Data was pooled from three prospective cohorts (NHS, NHS II, and HPFS; total n = 205,107). Associations were estimated using restricted cubic spline models with three knots placed at the 10th, 50th, and 90th percentiles of intake, including interaction terms between cohort and covariates.
Supplementary Figure 4. Cohort-specific dose–response relationships between (A) total potato intake, (B) baked/boiled/mashed potatoes, and (C) French fries and the incidence of type 2 diabetes in the NHS, NHS II, and HPFS
Supplementary Figure 5. Meta-analysis search strategy and study selection
Supplementary Figure 6. Forest plot indicating summary hazard ratio of the association between 3-serving/week total potato intake and T2D: A) fixed-effects model, B: random-effects model.
Supplementary Figure 7. Forest plot indicating summary hazard ratio of the association between 3-serving/week fried potato intake and T2D: A) fixed-effects model, B: random-effects model.
Supplementary Figure 8. Forest plot indicating summary hazard ratio of the association between 3-serving/week non-fried potato intake and T2D: A) fixed-effects model, B: random-effects model.
Supplementary Figure 9. Forest plot indicating summary hazard ratio of the association between 3-serving/week boiled potato intake and T2D: A) fixed-effects model, B: random-effects model.
Supplementary Figure 10. Forest plot indicating summary hazard ratio of the association between 3-serving/week mashed potato intake and T2D: A) fixed-effects model, B: random-effects model.
Supplementary Figure 11. Leave-one-out sensitivity analysis of the association between 3-serving/week **total potato** consumption and risk of T2D.
Supplementary Figure 12. Leave-one-out sensitivity analysis of the association between 3-serving/week **fried potato** consumption and risk of T2D.
Supplementary Figure 13. Leave-one-out sensitivity analysis of the association between 3-serving/week **non-fried potato** consumption and risk of T2D.
Supplementary Figure 14. Leave-one-out sensitivity analysis of the association between 3-serving/week **boiled potato** consumption and risk of T2D.
Supplementary Figure 15. Nonlinear dose-response association between potato consumption and risk of T2D
Supplementary Figure 16. Funnel plot for assessment of bias due to small studies for the association between 3-serving/week total potato consumption and risk of T2D.
Supplementary Figure 17. Summary Haz ard ratio of T2D incidence per 3 servings/week increase in whole grains consumption, using fixed effects model.
Supplementary Figure 18. The effect of replacing 3-serving/week of whole grain for different form of potatoes on T2D incidence, based on the pooled results of whole grains and potatoes meta-analyses.
Supplementary Figure 19. The effect of replacing 3-serving/week of whole grain for different forms of potatoes on T2D incidence, using pooled data from meta-analyses of whole grains and potatoes: (A) limiting the analysis to the four overlapping cohorts (NHS, NHS II, HPFS, and UK Biobank); (B) studies with energy adjustment; (C) US-based studies; (D) studies with repeated dietary assessments.
Supplementary references

Supplementary Methods

Dose-response meta-analysis of potato intake and T2D

A systematic review and an updated dose-response meta-analysis were conducted to assess the association between potato consumption and T2D incidence, incorporating findings from this study and other relevant prospective cohort studies. The results were reported in adherence to the Preferred Reporting Items for Systematic Reviews and Meta-Analyses (PRISMA) guidelines, with the study protocol pre-registered at PROSPERO (http://www.crd.york.ac.uk/PROSPERO, ID = CRD42023448736). Comprehensive searches were performed in PubMed/Medline, ISI Web of Science, and Embase databases without applying any restrictions or filters, up to July 2023, with an additional update until July 2024 using predefined search terms (**supplementary Table 2**). We also manually reviewed the reference lists of pertinent meta-analyses, reviews, and original studies, as well as key journals, to identify more eligible studies. Studies were included if they: 1) were prospective cohort studies without pre-existing diseases at baseline; 2) examined the association between T2D incidence and total or specific types of potato intake as a primary or secondary exposure; 3) provided a quantitative measure of potato consumption, including servings per day or week, cups per day or week, and grams per day or week, for two or more categories as well as multivariable adjusted relative risks (RRs) or hazard ratios (HRs) with 95% CIs, along with the total or category-specific number of cases and non-cases or person-years. Studies presenting associations in continuous terms were also considered. In cases of multiple publications from the same cohort, the report with the most cases or longest follow-up was selected.

We extracted the following information from each study: the first author's name, cohort name, publication year, geographical location, follow-up duration, total participants and T2D cases, gender of participants, their average and range of ages at baseline, method of assessing dietary intake, T2D cases ascertainment method, statistical analysis model, frequency, quantity, and units of potato consumption, fully adjusted risk estimates with 95% CIs, and a list of adjusted confounders. We evaluated the potential for bias in the included studies with Newcastle-Ottawa Scale (NOS) [1] and the Risk of Bias in Non-randomized Studies of Exposure (ROBINS-E) tool.[2] The NOS is designed to evaluate the quality of non-randomized studies, with a maximum score of nine. This scoring system assesses studies using specific criteria in three domains: selection (including representativeness of the exposed and selection of the non-exposed cohorts, exposure assessment method, and the absence of outcome at baseline), outcome ascertainment (considering follow-up length, adequacy of follow-up, and method of outcome assessment), and the comparability (controlling for primary and secondary confounding variables). Primary confounding variables include age, sex, BMI, smoking status, physical activity, alcohol intake, and total energy, all of which were selected due to their association with T2D risk. Additionally, controlling the consumption of key food groups, including red meats, whole and refined grains, fruits, vegetables, fish, eggs, sugar-sweetened beverages, poultry, dairy products, nuts, and legumes is considered as secondary confounders. The overall quality of the studies is categorized based on their scores, with 7 to 9 indicating a low risk of bias (high quality), 4 to 6 suggesting a moderate, and 0 to 3 signifying a high risk of bias (low quality). The ROBINS-E tool evaluates the risk of bias across seven key domains: confounding factors, exposure measurement, participant selection, post-exposure interventions, missing data, outcome measurement, and selective result reporting. Each domain, along with the overall risk of bias, was categorized as low risk, some

concerns, or high risk. The overall certainty of evidence for each association was judged using two tools: the revised Grading of Recommendations, Assessment, Development, and Evaluations (GRADE) approach[3] and NutriGrade [4]. The NutriGrade was used to address the argument that GRADE may not adequately consider the potential value of prospective cohort studies in nutrition assessments.[5] However, in the latest revision of GRADE, the initial evidence level for observational studies is considered "high," unlike the previous versions. The search terms were defined by the first author (SMM), who also performed literature screening, including title and abstract review, full-text evaluation, data extraction, and bias risk assessment. An independent second reviewer (FI) cross-checked the screening, data extraction, and bias assessment, with any discrepancies resolved through consensus.

HRs were the most used measure of the associations in the included cohort studies, with ORs and RRs being considered equivalent to HRs. We determined HRs and their 95% CIs for every 3-servings/week increase in potato consumption using the method established by Greenland, Longnecker [6], and Orsini [7]. This required information on the distribution of cases and person-years, and the median or range of potato consumption with related effect estimates across different intake levels. When studies reported only total cases or person-years for categorized exposures, we estimated the per-category figures by dividing the total by the number of categories. The dosage of potato consumption was determined by using the reported median for each category, or by estimating an average from the provided range. For categories with open-ended highest or lowest values (e.g., ≥2 servings/day), we assumed their intervals to be equal to those of the adjacent categories. In cases where potato intake was reported by grams, we assigned a standard weight of 150 g per serving. To explore non-linear relationships, we applied restricted cubic splines with three knots at specific distribution percentiles (10%, 50%, and 90%) [8]. The correlations within

each HR category were evaluated before combining the findings from each study into a one-stage linear mixed-effects meta-analysis [9]. This one-stage approach, different from the traditional two-stage method, estimates the slope lines for each study and combines them into an overall average slope, offering increased accuracy, adaptability, and efficiency [10].

To estimate the average actual effect size, we derived the summary HRs and 95% CIs for an increment of 3 servings/week of potato intake, employing a fixed-effects inverse-variance weighted model [11]. We also reported the random-effects DerSimonian and Laird model as the secondary approach [12]. The heterogeneity among studies was evaluated via Cochran's Q test [13] and the I^2 statistic [14], interpreting findings based on the thresholds set by the Cochrane Handbook [15]. To explore potential heterogeneity sources, we conducted subgroup and meta-regression analyses, considering factors such as follow-up duration, gender, method of dietary assessment, geographical location, number of cases and participants, risk of bias, method of T2D case ascertainment (self-reported only vs. supported by additional information), and the adjustment for primary and secondary confounders. An influence analysis was also performed, sequentially omitting each cohort and recalculating the HR, to check the robustness of the combined effect sizes. For our comparative effectiveness meta-analysis (substitution meta-analysis), we estimated the HR of substituting 3 servings/week whole grain for potato intake. We prioritized whole grain substitutions largely because of the substantial and consistent evidence demonstrating their wide-ranging health benefits, coupled with their low consumption levels worldwide, to better inform dietary guidelines.[16] The former obtained from previously published dose-response meta-analyses [17] and the latter from our current meta-analysis. We first calculated β coefficients (log HRs) for every 3 servings/week of whole grains and potato exposures (i.e., total, fried, and non-fried potato). Then, using the variances of these coefficients and their covariance, the 95% CIs for this difference

were estimated. The difference was then exponentiated to determine the HR for each substitution [18]. The covariance between the two coefficients was estimated from the multivariate meta-analysis of NHSI, NHSII and HPFS. This approach was assumed to be valid, given the substantial weight of our cohorts in both meta-analyses. As the secondary analysis, we also modeled two distinct approaches: initially, we treated the variables as independent (r = 0). Subsequently, we adjusted our analysis to consider a slight correlation between these variables (r = 0.20). This dual-method strategy allowed us to understand the impact of assuming different levels of interdependence between whole grain and potato consumption on our results. Sensitivity analyses were also conducted to test the robustness of assumptions against sources of heterogeneity. Given that the first author (SMM) had already analyzed the published meta-analysis on the relationship between whole grains and T2D, we decided to update the search date of the previously published article up to July 2024 to ensure that we included all available evidence in this regard. We found two more cohort study [19 20] and combined it with the previously included studies. When at least ten studies were available, funnel plots and Egger's regression test [21] were employed to examine publication bias and small study effects. All statistical analyses were performed using Stata software version 17.0 (StataCorp.).

Supplementary Table 1. Gram weight for 1 serving of different potatoes over time in NHS, NHS II, and HPFS.

	Baked, boiled, mashed potato	French Fries	Chips	Baked, boiled, mashed potato	French Fries	Chips
	**Grams**			**Serving size**		
**NHS**						
1984	210	112	28	1 medium/1 cup	4 oz	1 oz
1986	210	112	28	1 medium/1 cup	4 oz	1 oz
1990	210	112	28	1 medium/1 cup	4 oz	1 oz
1994	156	112	28	1 medium/1 cup	4 oz	1 oz
1998	195	112	28	1 medium/1 cup	4 oz	1 oz
2002	177	170	28	1 medium/1 cup	6 oz	1 oz
2006	177	170	28	1 medium/1 cup	6 oz	1 oz
2010	177	170	28	1 medium/1 cup	6 oz	1 oz
**NHS II**						
1991	210	112	28	1 medium/1 cup	4 oz	1 oz
1995	156	112	28	1 medium/1 cup	4 oz	1 oz
1999	195	112	28	1 medium/1 cup	4 oz	1 oz
2003	177	170	28	1 medium/1 cup	6 oz	1 oz
2007	177	170	28	1 medium/1 cup	6 oz	1 oz
2011	177	170	28	1 medium/1 cup	6 oz	1 oz
2015	177	170	28	1 medium/1 cup	6 oz	1 oz
2019	190.76	170	28	1 medium/1 cup	6 oz	1 oz
**HPFS**						
1986	210	112	28	1 medium/1 cup	4 oz	1 oz
1990	156	112	28	1 medium/1 cup	4 oz	1 oz
1994	156	112	28	1 medium/1 cup	4 oz	1 oz
1998	195	112	28	1 medium/1 cup	4 oz	1 oz
2002	177	170	28	1 medium/1 cup	6 oz	1 oz
2006	177	170	28	1 medium/1 cup	6 oz	1 oz
2010	177	170	28	1 medium/1 cup	6 oz	1 oz
2014	177	170	28	1 medium/1 cup	6 oz	1 oz

Abbreviations: NHS, Nurses’ Health Study; NHS II, Nurses’ Health Study II; HPFS, Health Professionals Follow-up Study

Supplementary Table 2. Components of food groups

**Food Groups**	**Components**
Total red meat	Beef/Pork hot dogs, Bacon, Processed meat sandwiches, Other processed meats, Hamburger-Lean or extra lean, Hamburger-Regular, Beef/Pork/Lamb as mixed dish, Pork as a main dish, Beef/Lamb as a main dish
Processed red meat	Beef/Pork hot dogs, Bacon, Processed meat sandwiches, Other processed meats
Unprocessed red meat	Hamburger-Lean or extra lean, Hamburger-Regular, Beef/Pork/Lamb as mixed dish, Pork as a main dish, Beef/Lamb as a main dish
Total dairy	Whole milk, Cream, Regular ice cream, Cream cheese, Regular cheese, Skim milk, 1%-2% milk, Frozen yogurt/sherbet, Plain yogurt, artificially sweetened yogurt, Sweetened yogurt, Cottage/Ricotta cheese, Low-fat/lite/nonfat cheese
Fish	Canned tuna, Breaded fish, Dark meat fish, other fish
Nuts and legumes	Peanut butter, Peanuts, Walnuts, Other nuts, Beans/Lentils, Peas/Lima beans
Poultry	Chicken or turkey hot dogs, Chicken/Turkey sandwich, other chicken/turkey-With skin, other chicken/turkey-Without skin
Eggs	Eggs-Regular, Eggs- Omega-3 fortified
Sugar-sweetened beverages	Carbonated and noncarbonated beverages with sugar (soft drinks, punch, lemonade, fruit drink, or sugared iced tea)
Starchy vegetables	Peas, lima beans, corn, and sweet potatoes/yams
Non-starchy vegetable	Tomatoes, Tomato sauce, String beans, Broccoli, Cauliflower, Cabbage, Brussels sprouts, Carrots, Mixed vegetables, Eggplant, Kale, Spinach, lettuce, Celery, Onion
Whole grains	Dry weight of the whole grain ingredients in whole wheat and whole wheat flour, whole oats and whole oat flour, whole cornmeal and corn flour, brown rice and brown rice flour, whole barley, whole rye and rye flour, bulgur, buckwheat, popcorn, amaranth, wheat bran, corn bran, oat bran, rice bran, and psyllium, wheat germ

**Supplementary Table 3. The percentage of missingness in exposures and covariates in our cohorts ***			
Variables	NHS (No. of person-years = 1,881,139)	NHSII (No. of person-years = 2,308,071)	HPFS (No. of person-years = 986,291)
Total potato	0	0	0
Baked, boiled, mashed potato	0	0	0
French fries	0	0	0
Age	0	0	0
Race (white, non-white)	3.6%	2.4%	0
Smoking status	0.3%	0.3%	1.0%
Alcohol intake	0	0	0
Physical activity	3.1	<0.1%	<0.1%
Body mass index	0.5%	0.4%	0.3%
Menopausal status and hormone use	5.2%	2.0%	-
Multivitamin use	0	0	0
Family history of T2D	0	0	0
Anti-hypertensive drug use	0	0	0
Cholesterol-lowering drug use	0	0	0
Hypertension at baseline	0	0	0
Socioeconomic status	3.4%	0.3%	0.2%

Abbreviations: NHS, Nurses' Health Study; NHS II, Nurses' Health Study II; HPFS, Health Professionals Follow-up Study
* Missingness values reflect the proportion of missing data after applying the last observation carried forward (LOCF) method, used due to the repeated assessment of variables over time in these cohorts.

**Supplementary Table 4**. Assessment of the proportional hazards assumption for the primary exposure (total potato) and all the predictors in the multivariable-adjusted models assessing T2D risk			
	NHS	NHSII	HPFS
	*p-value**	*p-value**	*p-value**
Interaction term between age (years) and predictors			
Primary exposure			
Total potato intake (3 servings/week)	0.14	0.29	0.61
Confounders			
Energy intake (kcal)	0.70	0.08	0.25
Race (white, non-white)	0.90	0.41	<0.001
Smoking status (never, past, current: 1-14 cigs/day, current: >15-24 cigs/day, current: >24 cigs/day)	0.05	0.31	0.09
Alcohol intake (non-alcohol drinker, 0-4.9 grams/day, 5-9.9 grams/day, 10-14.9 grams/day, 15-29.9 grams/day, >30 grams/day)	0.27	0.29	0.09
Physical activity (<3, 3-9, 9-18, 18-27, ≥ 27 METs-hr/week)	0.77	0.54	<0.001
BMI (<21, 21-23, 23-25, 25-27, 27-30, 30-33, 33-35, 35-40, ≥40 kg/m2)	<0.001	<0.001	<0.001
Menopausal status and hormone use	0.80	<0.001	-
Multivitamin use	0.32	0.64	0.95
family history of T2D	<0.001	0.01	0.10
Anti-hypertensive drug use	0.27	0.01	0.22
Cholesterol-lowering drug use	0.08	0.82	0.40
Hypertension at baseline	<0.001	<0.001	<0.001
Quintiles of socioeconomic status	0.95	0.81	0.14
Quintiles of red meat intake	0.28	0.07	0.13
Quintiles of poultry intake	0.64	0.70	0.94
Quintiles of fish intake	0.53	0.05	0.17
Quintiles of egg intake	0.63	0.44	0.21
Quintiles of total dairy intake	0.06	0.24	0.002
Quintiles of nuts and legumes intake	0.61	0.83	0.14
Quintiles of fruits intake	0.62	0.49	0.51
Quintiles of vegetables intake	0.85	0.21	0.42
Quintiles of SSB intake	0.03	0.24	0.20
Quintiles of whole grains intake	0.16	0.42	0.72
Quintiles of refined grains intake	0.63	0.82	0.39

* *p-value < 0.05* suggests a potential violation of the proportional hazards assumption.
Abbreviations: NHS, Nurses' Health Study; NHS II, Nurses' Health Study II; HPFS, Health Professionals Follow-up Study; BMI, body mass index; MET, metabolic equivalent of task; T2D, type 2 diabetes;

Supplementary Table 5. Assessment of the proportional hazards assumption for the primary exposure (baked, boiled, mashed potato) and all the predictors in the multivariable-adjusted models assessing T2D risk

	NHS	NHSII	HPFS
	*p-value**	*p-value**	*p-value**
Interaction term between age (years) and predictors			
Primary exposure			
Baked, boiled, and mashed potato intake (3 servings/week)	0.48	0.53	0.95
Confounders			
Energy intake (kcal)	0.68	0.07	0.20
Race (white, non-white)	0.94	0.40	<0.001
Smoking status (never, past, current: 1-14 cigs/day, current: >15-24 cigs/day, current: >24 cigs/day)	0.05	0.30	0.09
Alcohol intake (non-alcohol drinker, 0-4.9 grams/day, 5-9.9 grams/day, 10-14.9 grams/day, 15-29.9 grams/day, >30 grams/day)	0.26	0.28	0.10
Physical activity (<3, 3-9, 9-18, 18-27, ≥ 27 METs-hr/week)	0.75	0.54	<0.001
BMI (<21, 21-23, 23-25, 25-27, 27-30, 30-33, 33-35, 35-40, ≥40 kg/m2)	<0.001	<0.001	<0.001
Menopausal status and hormone use	0.79	<0.001	-
Multivitamin use	0.33	0.63	0.97
Family history of T2D	<0.001	<0.001	0.10
Anti-hypertensive drug use	0.26	0.003	0.22
Cholesterol-lowering drug use	0.08	0.81	0.41
Hypertension at baseline	<0.001	<0.001	<0.001
Quintiles of socioeconomic status	0.96	0.80	0.15
Quintiles of red meat intake	0.38	0.10	0.16
Quintiles of poultry intake	0.62	0.71	0.97
Quintiles of fish intake	0.55	0.05	0.16
Quintiles of egg intake	0.60	0.43	0.19
Quintiles of total dairy intake	0.08	0.21	0.01
Quintiles of nuts and legumes intake	0.61	0.86	0.15
Quintiles of fruits intake	0.59	0.53	0.52
Quintiles of vegetables intake	0.77	0.19	0.43
Quintiles of SSB intake	0.05	0.27	0.18
Quintiles of whole grains intake	0.20	0.40	0.67
Quintiles of refined grains intake	0.55	0.79	0.41
Quintiles of French fries intake	0.02	0.19	0.66

* *p-value < 0.05* suggests a potential violation of the proportional hazards assumption.
Abbreviations: NHS, Nurses' Health Study; NHS II, Nurses' Health Study II; HPFS, Health Professionals Follow-up Study; BMI, body mass index; MET, metabolic equivalent of task; T2D, type 2 diabetes;

Supplementary Table 6. Assessment of the proportional hazards assumption for the primary exposure (French fries) and all the predictors in the multivariable-adjusted models assessing T2D risk

	NHS	NHSII	HPFS
	*p-value**	*p-value**	*p-value**
Interaction term between age (years) and predictors			
Primary exposure			
French fries intake (3 servings/week)	0.15	0.29	0.97
Confounders			
Energy intake (kcal)	0.78	0.08	0.16
Race (white, non-white)	0.90	0.40	<0.001
Smoking status (never, past, current: 1-14 cigs/day, current: >15-24 cigs/day, current: >24 cigs/day)	0.06	0.31	0.09
Alcohol intake (non-alcohol drinker, 0-4.9 grams/day, 5-9.9 grams/day, 10-14.9 grams/day, 15-29.9 grams/day, >30 grams/day)	0.25	0.29	<0.001
Physical activity (<3, 3-9, 9-18, 18-27, ≥ 27 METs-hr/week)	0.76	0.53	<0.001
BMI (<21, 21-23, 23-25, 25-27, 27-30, 30-33, 33-35, 35-40, ≥40 kg/m2)	<0.001	<0.001	<0.001
Menopausal status and hormone use	0.78	<0.001	-
Multivitamin use	0.30	0.65	0.92
Family history of T2D	<0.001	0.002	0.10
Anti-hypertensive drug use	0.24	0.003	0.25
Cholesterol-lowering drug use	0.08	0.82	0.44
Hypertension at baseline	<0.001	<0.001	<0.001
Quintiles of socioeconomic status	0.92	0.81	0.15
Quintiles of red meat intake	0.24	0.07	0.17
Quintiles of poultry intake	0.67	0.72	0.99
Quintiles of fish intake	0.52	0.05	0.17
Quintiles of egg intake	0.61	0.43	0.18
Quintiles of total dairy intake	0.07	0.23	0.002
Quintiles of nuts and legumes intake	0.65	0.86	0.16
Quintiles of fruits intake	0.62	0.48	0.49
Quintiles of vegetables intake	0.71	0.20	0.37
Quintiles of SSB intake	0.04	0.26	0.23
Quintiles of whole grains intake	0.20	0.42	0.78
Quintiles of refined grains intake	0.60	0.81	0.41
Quintiles of French fries intake	0.70	0.58	0.21

* *p-value < 0.05* suggests a potential violation of the proportional hazards assumption.
Abbreviations: NHS, Nurses' Health Study; NHS II, Nurses' Health Study II; HPFS, Health Professionals Follow-up Study; BMI, body mass index; MET, metabolic equivalent of task; T2D, type 2 diabetes;

**Supplementary Table 7.** Systematic review search strategy.[1]			
**Database**	**Step**	**Search terms**	**Results, n**
Pubmed	1	Potato* OR "solanum tuberosum" OR "white potato*" OR "french fry" OR "french fries" OR "Potato chip*" OR "Fried potato" OR Chips OR "starchy vegetables"	54,721
	2	Diabetes mellitus, type 2[MeSH Terms] OR diabetes mellitus[MeSH Terms] OR Diabetes[Title/Abstract] OR "Type 2 diabetes"[Title/Abstract] OR "diabetes mellitus"[Title/Abstract] OR Diabetic[Title/Abstract] OR "Diabetes type 2"[Title/Abstract] OR "Type II diabetes"[Title/Abstract] OR "Type 2 diabetes mellitus"[Title/Abstract] OR "Non-insulin dependent diabetes"[Title/Abstract] OR NIDDM[Title/Abstract] OR "Diabetes mellitus non-insulin dependent"[Title/Abstract] OR "Diabetes non-insulin dependent"[Title/Abstract] OR "Non-insulin-dependent diabetes mellitus"[Title/Abstract] OR "Diabetes risk"[Title/Abstract] OR "Noninsulin-dependent diabetes mellitus"[Title/Abstract] OR "Noninsulin dependent diabetes mellitus"[Title/Abstract]	885,156
	3	Cohort* OR case-cohort OR "nested case-control" OR population-based OR incident* OR incidence* OR Prospective OR Longitudinal OR Observational OR Follow-Up OR "Relative risk" OR "Hazard ratio" OR "incidence rate ratio"	4,555,698
	4	#1 AND #2 AND #3	**181**
Web of Science	1	TS= (Potato* OR "solanum tuberosum" OR "white potato*" OR "french fry" OR "french fries" OR "Potato chip*" AND "Fried potato" OR "starchy vegetables")	109,165
	2	TS= (Diabetes OR "Type 2 diabetes" OR "diabetes mellitus" OR Diabetic OR "Diabetes type 2" OR "Type II diabetes" OR "Type 2 diabetes mellitus" OR "Non-insulin dependent diabetes" OR NIDDM OR "Diabetes mellitus non-insulin dependent" OR "Diabetes non-insulin dependent" OR "Non-insulin-dependent diabetes mellitus" OR "Diabetes risk" OR "Noninsulin-dependent diabetes mellitus" OR "Noninsulin dependent diabetes mellitus")	993,103
	3	TS= (Cohort* OR case-cohort OR "nested case-control" OR population-based OR incident* OR incidence* OR Prospective OR Longitudinal OR Observational OR Follow-Up OR "Relative risk" OR "Hazard ratio" OR "incidence rate ratio")	4,693,117
	4	#1 AND #2 AND #3	**154**
Embase	1	potato:ti,ab,kw OR 'white potato*':ti,ab,kw OR 'french fry':ti,ab,kw OR 'french fries':ti,ab,kw OR 'potato chip':ti,ab,kw OR 'potato chips':ti,ab,kw OR 'fried potato':ti,ab,kw OR chips:ti,ab,kw OR 'starchy vegetables':ti,ab,kw	49,435

	2	diabetes:ti,ab,kw OR 'diabetes mellitus':ti,ab,kw OR 'type 2 diabetes':ti,ab,kw OR diabetic:ti,ab,kw OR 'diabetes type 2':ti,ab,kw OR 'type ii diabetes':ti,ab,kw OR 'type 2 diabetes mellitus':ti,ab,kw OR 'non insulin dependent diabetes mellitus':ti,ab,kw OR niddm:ti,ab,kw OR 'diabetes mellitus non-insulin dependent':ti,ab,kw OR 'diabetes non-insulin dependent':ti,ab,kw OR 'non-insulin-dependent diabetes mellitus':ti,ab,kw OR 'diabetes risk':ti,ab,kw OR 'noninsulin-dependent diabetes mellitus':ti,ab,kw OR 'noninsulin dependent diabetes mellitus':ti,ab,kw	1,284,444
	3	cohort:ti,ab,kw OR 'case cohort':ti,ab,kw OR 'nested case-control':ti,ab,kw OR 'population based':ti,ab,kw OR incident*:ti,ab,kw OR incidence*:ti,ab,kw OR prospective:ti,ab,kw OR longitudinal:ti,ab,kw OR observational:ti,ab,kw OR 'follow up':ti,ab,kw OR 'relative risk':ti,ab,kw OR 'hazard ratio':ti,ab,kw OR 'incidence rate ratio':ti,ab,kw	5,821,362
	4	(#1 AND #2 AND #3)	**168**

[1] Search date:07/12/2024

Supplementary Table 8. Eligibility criteria by the PICOS statement

	**Inclusion criteria**	**Exclusion criteria**
**P** (population)	General healthy adult population	Populations with pre-existing diseases, studies on children, adolescents, or pregnant women
**I** (intervention/exposure)	Dietary intake of Potatoes including total potato, fried potato, boiled potato, mashed potato, non-fried potato	Supplements or as a component of other food groups
**C** (comparison)	Dose–response relation	Not reporting dosage across categories
**O** (outcome)	Type 2 diabetes incidence	Biomarkers of type 2 diabetes risk (e.g. fasting glucose, HbA1c, etc.)
**S** (study design)	Prospective observational studies	Retrospective studies, in vitro/animal studies, cross-sectional and case-control studies

Supplementary Table 9. Hazard ratios and 95% confidence intervals for T2D associated with potatoes intake (3 servings/week) in the NHS (n=72,712), NHS II (n=90,232), and HPFS (n=42,163); models with covariate-specific non-proportional hazards

**Model***	**Cases**	**Person-years**	**Total potato**	**Baked, boiled, or mashed potatoes**	**French fries**
**NHS**	9,625	1,881,139	1.04 (1.00 to 1.09)	1.02 (0.97 to 1.07)	1.27 (1.11 to 1.45)
**NHSII**	8698	2,308,071	1.03 (0.98 to 1.09)	1.00 (0.94 to 1.07)	1.11 (1.01 to 1.22)
**HPFS**	3976	986,291	1.05 (0.99 to 1.11)	1.01 (0.94 to 1.08)	1.21 (1.07 to 1.36)
**Pooled**	22,299	5,175,501	1.04 (1.01 to 1.07)	1.01(0.97 to 1.04)	1.18 (1.10 to 1.26)

* The model was stratified by age (months) and calendar time (two-year interval) and adjusted for total energy intake, race/ethnicity, smoking status, alcohol intake, physical activity, multivitamin use, menopausal status and hormone use (if NHS or NHS II), family history of type 2 diabetes, anti-hypertensive drug use, cholesterol-lowering drug use, history of hypertension, socioeconomic status, time-varying body mass index and dietary covariates intakes (including total red meat, poultry, fish, egg, total dairy, nuts and legumes, fruits, vegetables, sugar-sweetened beverages, whole grain, refined grain, and mutual adjustment for different potatoes (serving/d; quintiles). In addition, interaction terms between log-transformed age and the following covariates were introduced to this model to account for non-proportional hazards: smoking status, BMI, family history of T2D, hypertension at baseline (NHS); BMI, family history of T2D, hypertension at baseline, menopausal status and hormone use, anti-hypertensive drug use (NHSII); race, alcohol intake, BMI, hypertension at baseline, physical activity (HPFS).

Abbreviations: NHS, Nurses' Health Study; NHS II, Nurses' Health Study II; HPFS, Health Professionals Follow-up Study; BMI, body mass index

Supplementary Table 10. Associations between potato intakes and risk of diabetes in the NHS (n=72,712), NHS II (n=90,232), and HPFS (n=42,163), adjusting for baseline BMI

	**Frequency of potato consumption (Serving/week)**						
**Total potato**	**<1**	**1**	**2-4**	**5 -6**	**≥7**	P-trend	**HR per 3 servings/week (95% CI)**
Cases/Person-years	788/ 270,016	2,172/676,037	11,458/2,645,546	6,705/1,338,773	1,176/245,130		
Model 1	Ref.	1.05 (0.97 to 1.14)	1.29 (1.19 to 1.38)	1.57 (1.45 to 1.69)	1.85 (1.68 to 2.03)	<0.001	1.30 (1.27 to 1.33)
Model 2 (updated BMI)	Ref.	0.99 (0.91 to 1.08)	1.05 (0.98 to 1.13)	1.13 (1.05 to 1.22)	1.25 (1.14 to 1.38)	<0.001	1.10 (1.07 to 1.13)
Model 2 (baseline BMI)	Ref.	1.00 (0.93 to 1.09)	1.07 (1.00 to 1.16)	1.15 (1.06 to 1.24)	1.28 (1.16 to 1.41)	<0.001	1.10 (1.07 to 1.13)
Model 3	Ref.	0.97 (0.90 to 1.06)	1.00 (0.92 to 1.07)	1.03 (0.95 to 1.11)	1.12 (1.02 to 1.24)	<0.001	1.05 (1.02 to 1.08)
**Baked, boiled, or mashed potatoes**	**<1**		**1**	**2-4**	**≥5**		
Cases/Person-years	1,917/594,299		4,204/1,196,275	12,754/2,731,261	3,462/653,665		
Model 1	Ref.		1.05 (0.99 to 1.10)	1.16 (1.11 to 1.22)	1.25 (1.18 to 1.33)	<0.001	1.13 (1.10 to 1.16)
Model 2 (updated BMI)	Ref.		1.00 (0.95 to 1.06)	1.01 (0.96 to 1.07)	1.06 (1.00 to 1.13)	0.008	1.05 (1.02 to 1.08)
Model 2 (baseline BMI)	Ref.		1.01 (0.96 to 1.07)	1.02 (0.97 to 1.07)	1.05 (0.98 to 1.11)	0.0.08	1.03 (1.00 to 1.06)
Model 3	Ref.		0.97 (0.92 to 1.02)	0.96 (0.91 to 1.01)	0.99 (0.93 to 1.05)	0.62	1.01 (0.98 to 1.05)
**French fries**	**Almost never**	**1-3/month**	**1**	**2-4**	**≥5**		
Cases/Person-years	2,952/985,067	10,803/2,542,718	5,234/1,103,370	3,130/517,430	180/26,919		
Model 1	Ref.	1.40 (1.34, 1.46)	1.77 (1.69, 1.86)	2.26 (2.14, 2.40)	3.17 (2.71 to 3.71)	<0.001	2.18 (2.09 to 2.28)
Model 2 (updated BMI)	Ref.	1.14 (1.09, 1.19)	1.22 (1.16, 1.28)	1.29 (1.22, 1.37)	1.44 (1.23 to 1.69)	<0.001	1.35 (1.27 to 1.44)
Model 2 (baseline BMI)	Ref.	1.16 (1.11, 1.21)	1.27 (1.21, 1.33)	1.36 (1.29, 1.45)	1.57 (1.34 to 1.84)	<0.001	1.44 (1.36 to 1.52)
Model 3	Ref.	1.09 (1.04, 1.13)	1.12 (1.06, 1.18)	1.15 (1.09, 1.23)	1.27 (1.08 to 1.49)	<0.001	1.20 (1.12 to 1.28)
**Chips (potato/corn)**	**Almost never**	**1-3/month**	**1**	**2-4**	**≥5**		
Cases/Person-years	2,763/787,691	8,361/1,945,784	4,893/1,112,988	5,435/1,137,550	847/191,488		
Model 1	Ref.	1.12 (1.07 to 1.17)	1.22 (1.16 to 1.28)	1.23 (1.17 to 1.29)	1.23 (1.14 to 1.34)	<0.001	1.15 (1.11 to 1.19)
Model 2 (updated BMI)	Ref.	1.02 (0.97 to 1.06)	1.05 (1.00 to 1.10)	1.04 (0.98 to 1.09)	1.07 (0.99 to 1.16)	0.01	1.07 (1.03 to 1.11)
Model 2 (baseline BMI)	Ref.	1.03 (0.98 to 1.08)	1.07 (1.02 to 1.12)	1.05 (1.00 to 1.11)	1.08 (1.00 to 1.17)	0.005	1.08 (1.04 to 1.12)

Model 3	Ref.	0.98 (0.94 to 1.03)	0.98 (0.93 to 1.03)	0.95 (0.90 to 1.00)	0.97 (0.89 to 1.06)	0.50	1.02 (0.98 to 1.06)

* Dietary intakes were cumulative averages from the baseline FFQ to the start of each 4-year follow-up interval.
Model 1 was stratified by age (months), calendar time (two-year interval), and adjusted for total energy intake.
Model 2 was additionally adjusted for race/ethnicity (white, non-white), smoking status (never, past, current: 1-14 cigs/day, current: >15-24 cigs/day, current: >24 cigs/day), alcohol intake (non-alcohol drinker, 0-4.9 grams/day, 5-9.9 grams/day, 10-14.9 grams/day, 15-29.9 grams/day, >30 grams/day), physical activity (<3, 3-9, 9-18, 18-27, ≥ 27 METs-hr/week), multivitamin use (yes/no), menopausal status and hormone use (if NHS or NHS II), family history of type 2 diabetes (yes/no), anti-hypertensive drug use (yes/no), cholesterol-lowering drug use (yes/no), history of hypertension at baseline (yes/no), socioeconomic status, and body mass index (<21, 21-23, 23-25, 25-27, 27-30, 30-33, 33-35, 35-40, ≥40 kg/m^2). All covariates (except race, family history of diabetes, and baseline hypertension) were updated every 2 years.
Model 2 (baseline BMI) was adjusted baseline body mass index (<21, 21-23, 23-25, 25-27, 27-30, 30-33, 33-35, 35-40, ≥40 kg/m2) instead of updated BMI.
Model 3 was adjusted for the covariates in model 2 + cumulative average intake of total red meat (serving/d; quintiles), poultry (serving/d; quintiles), fish (serving/d; quintiles), egg (serving/d; quintiles), total dairy (serving/d; quintiles), nuts and legumes (serving/d; quintiles), fruits (serving/d; quintiles), vegetables (serving/d; quintiles), SSB (serving/d; quintiles), whole grains (serving /d; quintiles) and refined grains (serving /d; quintiles), and mutual adjustment for baked, boiled or mashed potatoes and French fries.
Abbreviations: NHS, Nurses' Health Study; NHS II, Nurses' Health Study II; HPFS, Health Professionals Follow-up Study; BMI, body mass index

Supplementary Table 11. Sensitivity analyses of risk of type 2 diabetes incidence for potato consumption in NHS, NHS II, and HPFS (n=205,107).[1]

	Frequency of potato consumption (Serving/week)					*P* for trend[2]	HR per 3 servings/week (95% CI)
**Total potato**	**<1**	**1**	**2-4**	**5 -6**	**≥7**		
Model adjusted for modified AHEI instead of individual foods[3]	Ref.	0.98 (0.90 to 1.07)	1.02 (0.95 to 1.10)	1.08 (1.00 to 1.16)	1.18 (1.07 to 1.30)	<0.001	1.07 (1.04 to 1.10)
Stop updating diet after intermediate outcomes[4]	Ref.	0.97 (0.89 to 1.05)	1.00 (0.93 to 1.07)	1.04 (0.96 to 1.12)	1.11 (1.01 to 1.21)	<0.001	1.05 (1.02 to 1.07)
Symptomatic diabetes[5]	Ref.	1.00 (0.87 to 1.15)	1.03 (0.90 to 1.16)	1.03 (0.90 to 1.17)	1.20 (1.02 to 1.39)	0.009	1.05 (1.00 to 1.09)
Excluding incident T2D cases in the first 10 years of follow-up	Ref.	0.98 (0.89 to 1.09)	1.00 (0.90 to 1.09)	1.03 (0.93 to 1.14)	1.11 (0.98 to 1.26)	0.006	1.04 (1.00 to 1.08)
**Baked, boiled, mashed potato**	**<1**		**1**	**2-4**	**≥5**		
Model adjusted for modified AHEI instead of individual foods[3]	Ref.		0.97 (0.92 to 1.03)	0.96 (0.92 to 1.02)	1.00 (0.94 to 1.06)	0.22	1.03 (0.99 to 1.06)
Stop updating diet after an intermediate outcome[4]	Ref.		0.98 (0.92 to 1.03)	0.96 (0.92 to 1.01)	1.00 (0.94 to 1.06)	0.57	1.01 (0.98 to 1.05)
Symptomatic diabetes[5]	Ref.		1.03 (0.94 to 1.13)	1.00 (0.92 to 1.09)	1.00 (0.90 to 1.10)	0.66	1.00 (0.95 to 1.06)
Excluding incident T2D cases in the first 10 years of follow-up	Ref.		0.96 (0.89 to 1.02)	0.95 (0.89 to 1.02)	0.99 (0.91 to 1.06)	0.48	1.01 (0.97 to 1.05)
**French fries**	**Almost never**	**1-3**	**1**	**2-4**	**≥5**		
Model adjusted for modified AHEI instead of individual foods[3]	Ref.	1.10 (1.06 to 1.15)	1.16 (1.10 to 1.22)	1.21 (1.14 to 1.29)	1.34 (1.14 to 1.57)	<0.001	1.27 (1.19 to 1.35)
Stop updating diet after an intermediate outcome[4]	Ref.	1.09 (1.04 to 1.14)	1.12 (1.06 to 1.18)	1.16 (1.09 to 1.23)	1.29 (1.10 to 1.51)	<0.001	1.18 (1.11 to 1.26)
Symptomatic diabetes[5]	Ref.	1.09 (1.01 to 1.17)	1.08 (0.99 to 1.17)	1.16 (1.06 to 1.28)	1.41 (1.13 to 1.77)	<0.001	1.21 (1.10 to 1.33)
Excluding incident T2D cases in the first 10 years of follow-up	Ref.	1.08 (1.02 to 1.13)	1.11 (1.04 to 1.18)	1.15 (1.06 to 1.23)	1.28 (1.04 to 1.56)	<0.001	1.21 (1.11 to 1.31)
Further adjustment for *trans-fat*	Ref.	1.10 (1.05 to 1.14)	1.14 (1.08 to 1.20)	1.20 (1.12 to 1.28)	1.33 (1.13 to 1.56)	<0.001	1.25 (1.17 to 1.34)
Further adjustment for confectionery intake	Ref.	1.12 (1.07 to 1.17)	1.18 (1.12 to 1.24)	1.22 (1.15 to 1.29)	1.30 (1.10 to 1.52)	<0.001	1.27 (1.19 to 1.35)
Adjustment for BMI (continuous) and BMI^2 instead of BMI (categorical)	Ref.	1.09 (1.04 to 1.13)	1.12 (1.06 to 1.18)	1.15 (1.08 to 1.23)	1.26 (1.07 to 1.48)	<0.001	1.19 (1.12 to 1.28)
Further adjustment for PRS for T2D[6]	Ref.	1.11 (1.02, 1.22)	1.16 (1.04 to 1.30)	1.29 (1.13 to 1.47)	1.47 (1.00 to 2.15)	<0.001	1.29 (1.12 to 1.49)

**Chips (potato/corn)**	**Almost never**	**1-3**	**1**	**2-4**	**≥5**		
Model adjusted for modified AHEI instead of individual foods[3]	Ref.	1.00 (0.95 to 1.04)	1.01 (0.96 to 1.06)	0.98 (0.93 to 1.03)	1.00 (0.93 to 1.09)	0.76	1.03 (1.00 to 1.07)
Stop updating diet after an intermediate outcome[4]	Ref.	0.99 (0.94 to 1.03)	0.98 (0.93 to 1.03)	0.96 (0.91 to 1.01)	0.97 (0.90 to 1.06)	0.54	1.01 (0.98 to 1.05)
Symptomatic diabetes[5]	Ref.	0.89 (0.83 to 0.96)	0.91 (0.84 to 0.98)	0.87 (0.80 to 0.95)	0.88 (0.77 to 1.00)	0.28	0.99 (0.94 to 1.06)
Excluding incident T2D cases in the first 10 years of follow-up	Ref.	1.02 (0.96 to 1.08)	1.02 (0.95 to 1.08)	0.98 (0.92 to 1.04)	1.00 (0.91 to 1.11)	0.49	1.01 (0.96 to 1.06

[1] Data are presented as hazard ratios (HR) and 95% confidence interval (CI) from Cox proportional hazards regression models.
[2] P-values for trend based on continuous potato variable derived from the median potato intake in each category of consumption.
[3] The model was stratified by age (months), calendar time (two-year interval), and adjusted for total energy intake, race/ethnicity (white adults, non-white adults), smoking status (never, past, current: 1-14 cigs/day, current: >15-24 cigs/day, current: >24 cigs/day), alcohol intake (non-alcohol drinker, 0-4.9 grams/day, 5-9.9 grams/day, 10-14.9 grams/day, 15-29.9 grams/day, >30 grams/day), physical activity (<3, 3-9, 9-18, 18-27, ≥ 27 METs-hr/week), multivitamin use, menopausal status and hormone use (if NHS or NHS II), family history of type 2 diabetes, anti-hypertensive drug use, cholesterol-lowering drug use, history of hypertension, socioeconomic status, time-varying body mass index (<21, 21-23, 23-25, 25-27, 27-30, 30-33, 33-35, 35-40, ≥40 kg/m^2), updated average of modified alternative healthy eating index (fifths), and mutual adjustment for different potatoes. All covariates (except race, family history of diabetes, baseline hypertension) were updated every 2 years.
[4] Cumulative average updates of potatoes and other dietary variables were stopped after self-report of angina, myocardial infraction, or coronary artery bypass graft procedure. Results are from multivariable model 3 which is stratified on age and adjusted for total energy intake, race/ethnicity (white adults, non-white adults), smoking status (never, past, current: 1-14 cigs/day, current: >15-24 cigs/day, current: >24 cigs/day), alcohol intake (non-alcohol drinker, 0-4.9 grams/day, 5-9.9 grams/day, 10-14.9 grams/day, 15-29.9 grams/day, >30 grams/day), physical activity (<3, 3-9, 9-18, 18-27, ≥ 27 METs-hr/week), multivitamin use, menopausal status and hormone use (if NHS or NHS II), family history of type 2 diabetes, anti-hypertensive drug use, cholesterol-lowering drug use, history of hypertension, socioeconomic status, body mass index (<21, 21-23, 23-25, 25-27, 27-30, 30-33, 33-35, 35-40, ≥40 kg/m2) and dietary covariates intakes (including total red meat, poultry, fish, egg, total dairy, nuts and legumes, fruits, vegetables, sugar-sweetened beverages, whole grain, and refined grain, and and mutual adjustment for different potatoes (serving/d; quintiles). All covariates (except race, family history of diabetes, baseline hypertension) were updated every 2 years.

[5]The outcome was restricted to symptomatic cases of diabetes at diagnosis (n=8,523), ascertained by the report of at least one symptom of diabetes in the supplementary questionnaire. Results are from multivariable model 3 (described above).

[6] The analysis included only participants with available genetic data (n=45,148, including 5,079 T2D cases), and the results were derived from multivariable Model 3.

Abbreviations: NHS, Nurses' Health Study; NHS II, Nurses' Health Study II; HPFS, Health Professionals Follow-up Study; BMI, body mass index; PRS, polygenic risk score; AHEI, Alternative healthy eating index.

Supplementary Table 12. Associations between 3-serving/week potato intake and risk of diabetes in the NHS, NHS II, and HPFS (n=205,107), stratified by key variables. [1 2]

			Total potato		Baked, boiled, mashed potato		French fries	
		Cases	HR (95% CI)	P-value	HR (95% CI)	P-value	HR (95% CI)	P-value
Age	< 65	14554	1.04 (1.01 to 1.08)	0.01	1.03 (0.99 to 1.07)	0.15	1.10 (1.03 to 1.19)	0.008
	>= 65	7789	1.02 (0.97 to 1.08)	0.40	1.00 (0.95 to 1.06)	0.99	1.20 (1.04 to 1.40)	0.01
	P-interaction		0.64		0.93		0.80	
mAHEI score	< median	13117	1.07 (1.03 to 1.10)	<0.001	1.03 (0.99 to 1.07)	0.21	1.21 (1.13 to 1.30)	<0.001
	>= median	9226	1.11 (1.06 to 1.17)	<0.001	1.04 (0.99 to 1.10)	0.12	1.59 (1.41 to 1.80)	<0.001
	P-interaction *		0.28		0.14		0.14	
Sex	Women	18329	1.07 (1.03 to 1.10)	<0.001	1.05 (1.01 to 1.09)	0.009	1.17 (1.08 to 1.26)	<0.001
	Men	3976	1.06 (1.00 to 1.12)	0.04	1.01 (0.94 to 1.08)	0.64	1.25 (1.10 to 1.41)	<0.001
	P-interaction		0.16		0.35		0.74	
Physical Activity (METs-hr/Week)	< median	13883	1.01 (0.98 to 1.05)	0.43	0.98 (0.94 to 1.03)	0.43	1.14 (1.05 to 1.24)	0.001
	>= median	8416	1.11 (1.06 to 1.16)	<0.001	1.07 (1.01 to 1.13)	0.01	1.37 (1.21 to 1.53)	<0.001
	P-interaction*		0.75		0.93		0.25	
BMI (kg/m^2)	< 25	2679	1.05 (0.97 to 1.13)	0.28	1.00 (0.91 to 1.09)	0.98	1.39 (1.13 to 1.73)	<0.001
	25-30	6862	1.01 (0.96 to 1.07)	0.63	0.97 (0.91 to 1.02)	0.25	1.29 (1.14 to 1.46)	<0.001
	>=30	12758	1.06 (1.02 to 1.10)	0.003	1.03 (0.98 to 1.07)	0.25	1.18 (1.09 to 1.28)	<0.001
	P-interaction*		<0.001		<0.001		<0.001	
Baseline hypertension	No	16502	1.04 (1.01 to 1.08)	0.02	1.01 (0.97 to 1.05)	0.69	1.19 (1.10 to 1.29)	<0.001
	Yes	5798	1.05 (1.00 to 1.11)	0.05	1.02 (0.96 to 1.08)	0.49	1.23 (1.08 to 1.40)	0.002
	P-interaction		0.37		0.51		0.35	
Race	White adults	20177	1.08 (1.05 to 1.11)	<0.001	1.05 (1.02 to 1.09)	0.004	1.22 (1.14 to 1.31)	<.0001
	Black adults	453	0.98 (0.76 to 1.27)	0.90	1.00 (0.72 to 1.38)	0.99	0.95 (0.60 to 1.50)	0.83
	Hispanic adults	367	1.10 (0.84 to 1.44)	0.49	1.05 (0.76 to 1.45)	0.78	1.30 (0.78 to 2.18)	0.31
	Asian adults	333	1.01 (0.73 to 1.38)	0.96	0.81 (0.52 to 1.27)	0.36	1.61 (0.89 to 2.94)	0.11
	Minorities [3]	1153	0.96 (0.83 to 1.12)	0.59	0.90 (0.74 to 1.09)	0.26	1.12 (0.84 to 1.50)	0.43
	P-interaction [4]		0.16		0.03		0.52	
	P-interaction [5]		0.01		0.01		0.23	
Smoking	Never	11771	1.05 (1.01 to 1.09)	0.01	1.00 (0.96 to 1.05)	0.90	1.26 (1.15 to 1.38)	<0.001

	Past	8544	1.05 (1.00 to 1.10)	0.05	1.03 (0.98 to 1.09)	0.28	1.16 (1.04 to 1.31)	0.01
	Current	1984	1.00 (0.91 to 1.09)	0.94	0.98 (0.88 to 1.09)	0.71	1.07 (0.87 to 1.32)	0.50
	P-interaction		0.08		0.21		0.08	

Abbreviations: NHS, Nurses' Health Study; NHS II, Nurses' Health Study II; HPFS, Health Professionals Follow-up Study; BMI, body mass index

[1] Dietary intake are cumulative averages from the baseline FFQ to the start of each 4-year follow-up interval.

[2] The model was stratified by age (months) and calendar time (two-year interval), and adjusted for total energy intake, race/ethnicity (white adults, non-white adults), smoking status (never, past, current: 1-14 cigs/day, current: >15-24 cigs/day, current: >24 cigs/day), alcohol intake (non-alcohol drinker, 0-4.9 grams/day, 5-9.9 grams/day, 10-14.9 grams/day, 15-29.9 grams/day, >30 grams/day), physical activity (<3, 3-9, 9-18, 18-27, ≥ 27 METs-hr/week), multivitamin use, menopausal status and hormone use (if NHS or NHS II), family history of type 2 diabetes, anti-hypertensive drug use, cholesterol-lowering drug use, history of hypertension, socioeconomic status, time-varying body mass index (<21, 21-23, 23-25, 25-27, 27-30, 30-33, 33-35, 35-40, ≥40 kg/m^2) and dietary covariates intakes (including total red meat, poultry, fish, egg, total dairy, nuts and legumes, fruits, vegetables, sugar-sweetened beverages, whole grain, refined grain, and mutual adjustment for different potatoes (serving/d; quintiles).

[3] Minorities included a combination of Black, Hispanic, and Asian adults. Participants with missing race/ethnicity were excluded.

[4] Interaction testing the null hypothesis that the associations between potato intakes and risk of diabetes do not differ among White, Black, Hispanic, and Asian adults.

[5] Interaction testing the null hypothesis that the associations between potato intakes and risk of diabetes do not differ between White adults and minorities.

* The P-interaction value was obtained from a model considering the continuous form of the variable, rather than its categorical subgroups.

Abbreviations: NHS, Nurses' Health Study; NHS II, Nurses' Health Study II; HPFS, Health Professionals Follow-up Study; BMI, body mass index; AHEI, Alternative healthy eating index.

**Supplementary Table 13.** Associations between potato intakes and risk of diabetes in the NHS (n=72,712), NHS II (n=90,232), and HPFS (n=42,163) using different types of dietary assessments [1 2]								
	**Potato intakes (servings/week)**							
**Total potato**	**<1**	**1**	**2-4**	**5 -6**	**≥7**	**P-trend**	**HR per 3 servings/week (95% CI)**	**P-value**
Cumulative average	Ref.	0.97 (0.90, 1.06)	1.00 (0.92, 1.07)	1.03 (0.95, 1.11)	1.12 (1.02, 1.24)	<0.001	1.05 (1.02, 1.08)	0.001
	788	2172	11458	6705	1176			
Baseline only	Ref.	1.00 (0.94, 1.05)	1.02 (0.97, 1.07)	1.06 (1.00, 1.12)	1.12 (1.05, 1.19)	<0.001	1.05 (1.02, 1.07)	<0.001
	2043	3613	7186	6462	2995			
Cumulative average without the most recent assessments	Ref.	0.94 (0.87, 1.02)	0.98 (0.92, 1.05)	1.02 (0.95, 1.10)	1.07 (0.98, 1.17)	0.001	1.05 (1.02, 1.07)	<0.001
	970	2314	10655	6894	1466			
Most recent three	Ref.	0.96 (0.90, 1.02)	0.96 (0.91, 1.02)	0.98 (0.92, 1.04)	1.06 (0.98, 1.15)	0.03	1.03 (1.00, 1.06)	0.05
	1943	2864	10355	5939	1198			
Simple update	Ref.	0.99 (0.95, 1.04)	1.01 (0.97, 1.06)	1.03 (0.98, 1.08)	1.08 (1.02, 1.15)	0.005	1.03 (1.01, 1.05)	0.01
	4525	4017	6736	5081	1940			
**Baked, boiled, mashed potato**	**<1**		**1**	**2-4**	**≥5**			
Cumulative average	Ref.		0.97 (0.92, 1.03)	0.96 (0.91, 1.02)	0.99 (0.93, 1.05)	0.62	1.01 (0.98, 1.05)	0.39
	1917		4204	12754	3462			
Baseline only	Ref.		1.00 (0.96, 1.04)	1.01 (0.97, 1.05)	1.08 (1.02, 1.14)	0.01	1.03 (1.01, 1.05)	0.01
	4429		7022	8682	2166			
Cumulative average without the most recent assessments	Ref.		0.97 (0.92, 1.02)	0.96 (0.91, 1.00)	1.00 (0.94, 1.06)	0.38	1.02 (0.99, 1.05)	0.30
	2260		4598	12037	3404			
Most recent three	Ref.		0.98 (0.93, 1.03)	0.96 (0.92, 1.00)	0.95 (0.90, 1.01)	0.48	1.00 (0.97, 1.03)	0.83
	3,464		4,643	11,337	2,855			
Simple update	Ref.		1.00 (0.96, 1.04)	0.99 (0.95, 1.03)	1.01 (0.95, 1.07)	0.85	1.01 (0.98, 1.04)	0.46
	6717		6365	7585	1632			
**French fries**	**Almost never**	**1-3**	**1**	**2-4**	**≥5**			
Cumulative average	Ref.	1.09 (1.04, 1.14)	1.12 (1.07, 1.18)	1.16 (1.09, 1.23)	1.28 (1.09, 1.50)	<0.001	1.20 (1.12, 1.28)	<0.001
	2952	10803	5234	3130	180			
Baseline only	Ref.	1.05 (1.02, 1.09)	1.07 (1.02, 1.11)	1.11 (1.05, 1.18)	1.36 (1.18, 1.57)	<0.001	1.11 (1.06, 1.16)	<0.001
	6377	8530	5242	1935	215			
Cumulative average without the most recent assessments	Ref.	1.06 (1.02, 1.11)	1.10 (1.05, 1.15)	1.13 (1.07, 1.20)	1.21 (1.04, 1.41)	<0.001	1.18 (1.11, 1.25)	<0.001
	3569	10235	5242	3053	200			
Most recent three	Ref.	1.06 (1.02, 1.10)	1.09 (1.04, 1.14)	1.12 (1.06, 1.19)	1.29 (1.10, 1.51)	<0.001	1.18 (1.10, 1.25)	<0.001
	5,211	10,173	4,215	2,522	178			
Simple update	Ref.	1.05 (1.02, 1.09)	1.11 (1.06, 1.16)	1.11 (1.04, 1.19)	1.23 (1.02, 1.49)	<0.001	1.13 (1.07, 1.19)	<0.001
	9752	7555	3718	1163	111			
**Chips (potato/corn)**	**Almost never**	**1-3**	**1**	**2-4**	**≥5**			
Cumulative average	Ref.	0.98 (0.94, 1.03)	0.98 (0.93, 1.03)	0.95 (0.90, 1.00)	0.97 (0.89, 1.06)	0.50	1.02 (0.98, 1.06)	0.44
	2763	8361	4893	5435	847			

Baseline only	Ref.	0.97 (0.94, 1.01)	0.97 (0.93, 1.01)	0.94 (0.90, 0.99)	0.91 (0.84, 0.99)	0.03	0.98 (0.95, 1.00)	0.09
	6454	6835	5147	3144	719			
Cumulative average without the most recent assessments	Ref.	0.97 (0.93, 1.02)	0.97 (0.93, 1.02)	0.95 (0.90, 1.00)	0.97 (0.90, 1.05)	0.38	1.00 (0.97, 1.04)	0.81
	3432	8078	4805	5108	876			
Most recent three	Ref.	0.98 (0.94, 1.02)	0.98 (0.93, 1.02)	0.97 (0.93, 1.02)	1.00 (0.93, 1.08)	0.73	1.03 (1.00, 1.07)	0.08
	4398	8372	3897	4807	825			
Simple update	Ref.	1.00 (0.96, 1.03)	1.00 (0.96, 1.05)	0.99 (0.94, 1.04)	1.13 (1.03, 1.23)	0.09	1.04 (1.02, 1.07)	0.003
	8451	6594	4131	2504	619			

The model stratified by age (months) and calendar time (two-year interval), and adjusted for total energy intake, race/ethnicity (white adults, non-white adults), smoking status (never, past, current: 1-14 cigs/day, current: >15-24 cigs/day, current: >24 cigs/day), alcohol intake (non-alcohol drinker, 0-4.9 grams/day, 5-9.9 grams/day, 10-14.9 grams/day, 15-29.9 grams/day, >30 grams/day), physical activity (<3, 3-9, 9-18, 18-27, ≥ 27 METs-hr/week), multivitamin use, menopausal status and hormone use (if NHS or NHS II), family history of type 2 diabetes, anti-hypertensive drug use, cholesterol-lowering drug use, history of hypertension, socioeconomic status, time-varying body mass index (<21, 21-23, 23-25, 25-27, 27-30, 30-33, 33-35, 35-40, ≥40 kg/m^2) and dietary covariates intakes (including total red meat, poultry, fish, egg, total dairy, nuts and legumes, fruits, vegetables, sugar-sweetened beverages, whole grain, and refined grain, and mutual adjustment for different potatoes (serving/d; quintiles).

Abbreviations: NHS, Nurses' Health Study; NHS II, Nurses' Health Study II; HPFS, Health Professionals Follow-up Study

Supplementary Table 14. Associations between every 3-serving/week increment in potato intakes and risk of diabetes in the NHS (n=72,712), NHS II (n=90,232), and HPFS (n=42,163) by latency period. [1 2]

	**0-4 yrs**		**4-8 yrs**		**8-12 yrs**		**12-16 yrs**		**16-20 yrs**		**20-24 yrs**		**24-28 yrs**	
	**HR (95% CI)**	**P-value**	**HR (95% CI)**	**P-value**	**HR (95% CI)**	**P-value**	**HR (95% CI)**	**P-value**	**HR (95% CI)**	**P-value**	**HR (95% CI)**	**P-value**	**HR (95% CI)**	**P-value**
**Cases**	22,299		20,594		18,342		15,163		11,090		6,749		3,190	
**Total potato**	1.03 (1.01 to 1.05)	0.01	1.02 (1.00 to 1.04)	0.08	1.04 (1.01 to 1.06)	0.002	1.05 (1.03 to 1.08)	<0.001	1.05 (1.02 to 1.08)	0.002	1.04 (1.00 to 1.08)	0.03	1.02 (0.96 to 1.08)	0.55
**Baked, boiled, mashed potato**	1.01 (0.98 to 1.03)	0.54	0.99 (0.97 to 1.02)	0.63	1.02 (0.99 to 1.04)	0.19	1.04 (1.01 to 1.07)	0.004	1.03 (1.00 to 1.06)	0.07	1.02 (0.98 to 1.07)	0.33	0.99 (0.93 to 1.06)	0.72
**French fries**	1.13 (1.07 to 1.19)	<0.001	1.15 (1.09 to 1.21)	<0.001	1.13 (1.07 to 1.19)	<0.001	1.12 (1.0 to 1.18)	<0.001	1.12 (1.04 to 1.19)	0.001	1.13 (1.04 to 1.23)	0.004	1.13 (1.00 to 1.28)	0.05

Abbreviations: NHS, Nurses' Health Study; NHS II, Nurses' Health Study II; HPFS, Health Professionals Follow-up Study

[2] The model was stratified by age (months) and calendar time (two-year interval), and adjusted for total energy intake, race/ethnicity (white adults, non-white adults), smoking status (never, past, current: 1-14 cigs/day, current: >15-24 cigs/day, current: >24 cigs/day), alcohol intake (non-alcohol drinker, 0-4.9 grams/day, 5-9.9 grams/day, 10-14.9 grams/day, 15-29.9 grams/day, >30 grams/day), physical activity (<3, 3-9, 9-18, 18-27, ≥ 27 METs-hr/week), multivitamin use, menopausal status and hormone use (if NHS or NHS II), family history of type 2 diabetes, anti-hypertensive drug use, cholesterol-lowering drug use, history of hypertension, socioeconomic status, time-varying body mass index (<21, 21-23, 23-25, 25-27, 27-30, 30-33, 33-35, 35-40, ≥40 kg/m^2) and dietary covariates intakes (including total red meat, poultry, fish, egg, total dairy, nuts and legumes, fruits, vegetables, sugar-sweetened beverages, whole grain, refined grain, and mutual adjustment for different potatoes (serving/d; quintiles).

Supplementary Table 15. Associations between every 3 servings/week potatoes intake and risk of diabetes (NHS, NHSII, HPFS) in different time periods.						
**Model 3**	**Cases**	**Person-years**	**Total potato**	**Baked, boiled, or mashed potatoes**	**French fries**	**Chips**
**Main analysis**	22,299	5,175,501	1.05 (1.02 to 1.08)	1.01 (0.98 to 1.05)	1.20 (1.12 to 1.28)	1.02 (0.98 to 1.06)
**1986-1998**	5,242	1,828,641	1.03 (0.98 to 1.07)	0.99 (0.94 to 1.05)	1.20 (1.07 to 1.33)	1.08 (1.02 to 1.15)
**1998-2010***	8,859	1,567,837	1.05 (1.01 to 1.09)	1.02 (0.98 to 1.07)	1.15 (1.06 to 1.25)	1.02 (0.98 to 1.07)
**2010-onward***	2,503	724,118	1.04 (0.97 to 1.11)	1.02 (0.94 to 1.11)	1.10 (0.96 to 1.25)	0.99 (0.91 to 1.08)

Abbreviations: NHS, Nurses' Health Study; NHS II, Nurses' Health Study II; HPFS, Health Professionals Follow-up Study

[2] The model was stratified by age (months) and calendar time (two-year interval), and adjusted for total energy intake, race/ethnicity (white adults, non-white adults), smoking status (never, past, current: 1-14 cigs/day, current: >15-24 cigs/day, current: >24 cigs/day), alcohol intake (non-alcohol drinker, 0-4.9 grams/day, 5-9.9 grams/day, 10-14.9 grams/day, 15-29.9 grams/day, >30 grams/day), physical activity (<3, 3-9, 9-18, 18-27, ≥ 27 METs-hr/week), multivitamin use, menopausal status and hormone use (if NHS or NHS II), family history of type 2 diabetes, anti-hypertensive drug use, cholesterol-lowering drug use, history of hypertension, socioeconomic status, time-varying body mass index (<21, 21-23, 23-25, 25-27, 27-30, 30-33, 33-35, 35-40, ≥40 kg/m^2) and dietary covariates intakes (including total red meat, poultry, fish, egg, total dairy, nuts and legumes, fruits, vegetables, sugar-sweetened beverages, whole grain, refined grain, and mutual adjustment for different potatoes (serving/d; quintiles).

*Dietary intake is the cumulative average intake from 1986 for NHS, HPFS and 1991 for NHSII

**Supplementary Table 16.** Associations between substituting 3-serving/week of other foods for 3-serving/week of potatoes intakes and the risk of type 2 diabetes in the NHS, NHS II, and HPFS (n=205,107)		
	**Model 3[2]**	
	**HR (95% CI)**	**P-value**
**Cases/ Person-years**	22,299/ 5,175,501	
**Total potato**		
Whole grains	0.92 (0.89 to 0.95)	<0.001
Refined grains	0.97 (0.95 to 1.00)	0.10
Non-starchy vegetables	0.95 (0.93 to 0.98)	0.01
Starchy vegetables	1.01 (0.96 to 1.07)	0.74
Legumes	0.94 (0.87 to 1.01)	0.08
White rice	1.15 (1.10 to 1.20)	<0.001
Brown rice	0.98 (0.90 to 1.06)	0.63
**Baked, boiled, mashed potato**		
Whole grains	0.96 (0.92 to 0.99)	0.01
Refined grains	1.01 (0.98 to 1.05)	0.46
Non-starchy vegetables	0.99 (0.95 to 1.02)	0.45
Starchy vegetables	1.04 (0.99 to 1.11)	0.15
Legumes	0.97 (0.90 to 1.05)	0.43
White rice	1.19 (1.14 to 1.25)	<0.001
Brown rice	1.02 (0.94 to 1.11)	0.65
**French fries**		
Whole grains	0.81 (0.75 to 0.86)	<0.001
Refined grains	0.85 (0.79 to 0.91)	<0.001
Non-starchy vegetables	0.83 (0.78 to 0.89)	<0.001
Starchy vegetables	0.88 (0.81 to 0.95)	0.001
Legumes	0.81 (0.73 to 0.89)	<0.001

White rice	0.99 (0.93 to 1.07)	0.88
Brown rice	0.84 (0.76 to 0.93)	<0.001

Abbreviations: NHS, Nurses' Health Study; NHS II, Nurses' Health Study II; HPFS, Health Professionals Follow-up Study

[1] Dietary intakes were cumulative averages from the baseline FFQ to the start of each 4-year follow-up interval.

[2] The model was stratified by age (months) and calendar time (two-year interval), and adjusted for total energy intake, race/ethnicity (white adults, non-white adults), smoking status (never, past, current: 1-14 cigs/day, current: >15-24 cigs/day, current: >24 cigs/day), alcohol intake (non-alcohol drinker, 0-4.9 grams/day, 5-9.9 grams/day, 10-14.9 grams/day, 15-29.9 grams/day, >30 grams/day), physical activity (<3, 3-9, 9-18, 18-27, ≥ 27 METs-hr/week), multivitamin use, menopausal status and hormone use (in NHS or NHS II), family history of type 2 diabetes, anti-hypertensive drug use, cholesterol-lowering drug use, history of hypertension, socioeconomic status, time-varying body mass index (<21, 21-23, 23-25, 25-27, 27-30, 30-33, 33-35, 35-40, ≥40 kg/m^2) and dietary covariates intakes (including total red meat, poultry, fish, egg, total dairy, nuts and legumes, fruits, vegetables, sugar-sweetened beverages, whole grain, and refined grain, and mutual adjustment for different potatoes), excluding the foods subject for substitutions. The models for brown rice and white rice did not include adjustments for whole grain and refined grain intakes, respectively.

**Supplementary Table 17.** Associations between substituting 3-serving/week of white rice for 3-serving/week of potatoes intakes and the risk of type 2 diabetes in the NHS, NHS II, and HPFS, limiting the analysis to white participants only		
	**Model 3**[2]	
	**HR (95% CI)**	**P-value**
**Substitute white rice for**		
Total potato	1.10 (1.03 to 1.17)	<0.001
Baked, boiled, mashed potato	1.13 (1.06 to 1.21)	<0.001
French fries	0.95 (0.87 to 1.04)	0.28

Abbreviations: NHS, Nurses' Health Study; NHS II, Nurses' Health Study II; HPFS, Health Professionals Follow-up Study

[1] Dietary intake were cumulative averages from the baseline FFQ to the start of each 4-year follow-up interval.

[2] The model was stratified by age (months) and calendar time (two-year interval), and adjusted for total energy intake, smoking status (never, past, current: 1-14 cigs/day, current: >15-24 cigs/day, current: >24 cigs/day), alcohol intake (non-alcohol drinker, 0-4.9 grams/day, 5-9.9 grams/day, 10-14.9 grams/day, 15-29.9 grams/day, >30 grams/day), physical activity (<3, 3-9, 9-18, 18-27, ≥ 27 METs-hr/week), multivitamin use, menopausal status and hormone use (in NHS or NHS II), family history of type 2 diabetes, anti-hypertensive drug use, cholesterol-lowering drug use, history of hypertension, socioeconomic status, time-varying body mass index (<21, 21-23, 23-25, 25-27, 27-30, 30-33, 33-35, 35-40, ≥40 kg/m^2) and dietary covariates intakes (including total red meat, poultry, fish, egg, total dairy, nuts and legumes, fruits, vegetables, sugar-sweetened beverages, whole grain, and refined grain, and mutual adjustment for different potatoes), excluding the foods subject for substitutions.

**Supplementary Table 18.** Associations between potatoes intake and risk of diabetes in the NHS, NHS II, and HPFS (Cohort-specific results)							
	**Frequency of potato consumption (Serving/week)**						
**NHS (n=72,712)**							
**Total potato**	**<1**	**1**	**2-4**	**5 -6**	**≥7**	**P-trend***	**HR per 3 servings/week (95% CI)**
Cases/Person-years	319/94,434	907/223,522	4,896 /949,658	3,041/527,229	462/86,297		
Model 1	Ref.	1.13 (0.99 to 1.28)	1.29 (1.15 to 1.45)	1.47 (1.30 to 1.66)	1.55 (1.34 to 1.79)	<0.001	1.18 (1.14 to 1.23)
Model 2	Ref.	1.07 (0.94 to 1.22)	1.12 (0.99 to 1.25)	1.22 (1.08 to 1.37)	1.27 (1.09 to 1.47)	<0.001	1.10 (1.05 to 1.14)
Model 3	Ref.	1.06 (0.93 to 1.20)	1.06 (0.94 to 1.20)	1.12 (0.99 to 1.26)	1.14 (0.98 to 1.33)	0.02	1.05 (1.00 to 1.09)
**Baked, boiled, or mashed potatoes**	**<1**		**1**	**2-4**	**≥5**		
Cases/Person-years	651/161,007		1,477/343,268	5,595/1,048,246	1,902/328,618		
Model 1	Ref.		1.00 (0.91 to 1.10)	1.07 (0.98 to 1.17)	1.11 (1.01 to 1.22)	0.003	1.07 (1.02 to 1.12)
Model 2	Ref.		0.97 (0.89 to 1.07)	0.98 (0.90 to 1.07)	1.03 (0.93 to 1.13)	0.13	1.05 (1.01 to 1.10)
Model 3	Ref.		0.94 (0.86 to 1.03)	0.92 (0.85 to 1.01)	0.96 (0.87 to 1.05)	0.89	1.02 (0.97 to 1.07)
**French fries**	**Almost never**	**1-3/ month**	**1**	**2-4**	**≥5**		
Cases/Person-years	1,761/490,403	5,325/993,334	1,832/305,521	691/89,297	16/2,585		
Model 1	Ref.	1.41 (1.33 to 1.49)	1.72 (1.60 to 1.84)	2.14 (1.95 to 2.35)	1.96 (1.20 to 3.21)	<0.001	2.82 (2.54 to 3.14)
Model 2	Ref.	1.18 (1.11 to 1.25)	1.25 (1.16 to 1.34)	1.36 (1.24 to 1.50)	0.98 (0.60 to 1.61)	<0.001	1.53 (1.36 to 1.73)
Model 3	Ref.	1.12 (1.06 to 1.19)	1.14 (1.06 to 1.23)	1.22 (1.10 to 1.34)	0.89 (0.54 to 1.46)	0.006	1.29 (1.13 to 1.47)
**Chips (potato/corn)**	**Almost never**	**1-3/ month**	**1**	**2-4**	**≥5**		
Cases/Person-years	1,452/360,400	3,921/751,316	1,980/367,259	1,961/346,412	311/55,752		
Model 1	Ref.	1.17 (1.10 to 1.24)	1.25 (1.16 to 1.34)	1.25 (1.16 to 1.34)	1.33 (1.17 to 1.50)	<0.001	1.14 (1.08 to 1.20)
Model 2	Ref.	1.09 (1.02 to 1.16)	1.12 (1.04 to 1.20)	1.11 (1.03 to 1.20)	1.22 (1.08 to 1.39)	0.006	1.10 (1.03 to 1.16)
Model 3	Ref.	1.05 (0.98 to 1.12)	1.05 (0.97 to 1.13)	1.02 (0.95 to 1.10)	1.10 (0.97 to 1.25)	0.62	1.03 (0.96 to 1.10)
**NHS II (n=90,232)**							
**Total potato**	**<1**	**1**	**2-4**	**5 -6**	**≥7**		
Cases/Person-years	306/128,794	882/339,088	4,711/1,233,950	2,398/519,632	401/86,607		
Model 1	Ref.	1.03 (0.91 to 1.18)	1.40 (1.25 to 1.58)	1.91 (1.69 to 2.15)	2.68 (2.30 to 3.13)	<0.001	1.52 (1.46 to 1.58)

Model 2	Ref.	0.96 (0.85 to 1.10)	1.03 (0.92 to 1.16)	1.09 (0.97 to 1.24)	1.28 (1.10 to 1.50)	<0.001	1.10 (1.05 to 1.14)
Model 3	Ref.	0.94 (0.82 to 1.07)	0.97 (0.85 to 1.09)	0.98 (0.86 to 1.12)	1.13 (0.96 to 1.33)	0.03	1.04 (0.99 to 1.09)
**Baked, boiled, or mashed potatoes**	**<1/wk**		**1/wk**	**2-4/wk**	**≥5/wk**		
Cases/Person-years	865/324,962		1919/640,870	5028/1,165,240	886/176,999		
Model 1	Ref.		1.09 (1.01 to 1.18)	1.32 (1.23 to 1.43)	1.55 (1.40 to 1.71)	<0.001	1.31 (1.24 to 1.38)
Model 2	Ref.		1.03 (0.95 to 1.12)	1.07 (0.99 to 1.16)	1.10 (0.99 to 1.21)	0.05	1.06 (1.00 to 1.12)
Model 3	Ref.		1.00 (0.92 to 1.08)	1.01 (0.93 to 1.09)	1.02 (0.92 to 1.13)	0.66	1.00 (0.94 to 1.07)
**French fries**	**Almost never**	**1-3/ month**	**1/wk**	**2-4/wk**	**≥5/wk**		
Cases/Person-years	624/304,167	3827/1,121,574	2468/584,906	1685/284,244	94/13,181		
Model 1	Ref.	1.45 (1.33 to 1.58)	1.99 (1.82 to 2.18)	2.69 (2.45 to 2.96)	4.07 (3.27 to 5.07)	<0.001	2.23 (2.12 to 2.35)
Model 2	Ref.	1.07 (0.98 to 1.16)	1.14 (1.04 to 1.25)	1.17 (1.06 to 1.29)	1.30 (1.04 to 1.62)	0.001	1.25 (1.15 to 1.37)
Model 3	Ref.	1.02 (0.93 to 1.11)	1.04 (0.95 to 1.15)	1.05 (0.95 to 1.16)	1.15 (0.92 to 1.45)	0.14	1.13 (1.03 to 1.25)
**Chips (potato/corn)**	**Almost never**	**1-3/ month**	**1/wk**	**2-4/wk**	**≥5/wk**		
Cases/Person-years	662/246790	3014/851789	2096/550401	2543/570265	383/88826		
Model 1	Ref.	1.04 (0.95 to 1.13)	1.17 (1.07 to 1.28)	1.25 (1.14 to 1.36)	1.30 (1.15 to 1.48)	<0.001	1.24 (1.18 to 1.31)
Model 2	Ref.	0.88 (0.81 to 0.96)	0.92 (0.84 to 1.01)	0.91 (0.83 to 1.00)	1.01 (0.88 to 1.15)	0.14	1.10 (1.04 to 1.16)
Model 3	Ref.	0.85 (0.78 to 0.93)	0.86 (0.78 to 0.94)	0.84 (0.77 to 0.92)	0.92 (0.81 to 1.05)	0.97	1.06 (1.00 to 1.12)
**HPFS (n=42,163)**							
**Total potato**	**<1**	**1**	**2-4**	**5 -6**	**≥7**		
Cases/Person-years	163/46,788	383/113,431	1851/461,938	1266/291,912	313/ 72,226		
Model 1	Ref.	0.95 (0.79 to 1.14)	1.08 (0.92 to 1.27)	1.22 (1.03 to 1.44)	1.38 (1.13 to 1.68)	<0.001	1.15 (1.09 to 1.21)
Model 2	Ref.	0.89 (0.74 to 1.08)	0.97 (0.82 to 1.14)	1.04 (0.88 to 1.24)	1.19 (0.97 to 1.45)	<0.001	1.10 (1.04 to 1.16)
Model 3	Ref.	0.88 (0.73 to 1.06)	0.93 (0.78 to 1.09)	0.95 (0.80 to 1.14)	1.07 (0.87 to 1.31)	0.0	1.06 (1.00 to 1.12)
**Baked, boiled, or mashed potatoes**	**<1**		**1**	**2-4**	**≥5**		
Cases/Person-years	398/ 108,330		799/ 212,137	2111/ 517,775	668/ 148,048		
Model 1	Ref.		1.02 (0.90 to 1.15)	1.00 (0.90 to 1.12)	1.07 (0.94 to 1.23)	0.27	1.01 (0.94 to 1.07)

Model 2	Ref.		0.99 (0.88 to 1.12)	0.95 (0.85 to 1.07)	1.06 (0.92 to 1.21)	0.31	1.03 (0.96 to 1.10)
Model 3	Ref.		0.96 (0.85 to 1.08)	0.91 (0.81 to 1.02)	1.00 (0.87 to 1.14)	0.71	1.01 (0.94 to 1.08)
**French fries**	**Almost never**	**1-3/ month**	**1**	**2-4**	**≥5**		
Cases/Person-years	567/190,497	1,651/427,810	934/212,943	754/143,889	70/11,153		
Model 1	Ref.	1.30 (1.18 to 1.43)	1.61 (1.44 to 1.79)	1.91 (1.70 to 2.15)	2.57 (1.99 to 3.31)	<0.001	1.71 (1.57 to 1.88)
Model 2	Ref.	1.11 (1.01 to 1.23)	1.28 (1.14 to 1.42)	1.36 (1.21 to 1.53)	1.84 (1.42 to 2.38)	<0.001	1.39 (1.24 to 1.54)
Model 3	Ref.	1.06 (0.96 to 1.18)	1.18 (1.05 to 1.32)	1.22 (1.08 to 1.39)	1.60 (1.23 to 2.08)	<0.001	1.25 (1.10 to 1.41)
**Chips (potato/corn)**	**Almost never**	**1-3/ month**	**1**	**2-4**	**≥5**		
Cases/Person-years	649/180,501	1426/342,679	817/195,328	931/220,873	153/46,910		
Model 1	Ref.	1.13 (1.02 to 1.24)	1.21 (1.09 to 1.35)	1.17 (1.05 to 1.30)	0.94 (0.79 to 1.13)	0.75	0.97 (0.89 to 1.05)
Model 2	Ref.	1.04 (0.95 to 1.15)	1.09 (0.98 to 1.22)	1.07 (0.96 to 1.20)	0.92 (0.77 to 1.11)	0.59	0.97 (0.90 to 1.06)
Model 3	Ref.	1.01 (0.92 to 1.12)	1.01 (0.91 to 1.14)	0.97 (0.86 to 1.09)	0.83 (0.68 to 1.00)	0.03	0.91 (0.83 to 1.00)

*P values for trend were based on a continuous potato variable derived from the median potato intake in each category of consumption.
Model 1 was stratified by age (months) and calendar time (two-year interval), and adjusted for total energy intake.
Model 2 was additionally adjusted for race/ethnicity (white, non-white), smoking status (never, past, current: 1-14 cigs/day, current: >15-24 cigs/day, current: >24 cigs/day), alcohol intake (non-alcohol drinker, 0-4.9 grams/day, 5-9.9 grams/day, 10-14.9 grams/day, 15-29.9 grams/day, >30 grams/day), physical activity (<3, 3-9, 9-18, 18-27, ≥ 27 METs-hr/week), multivitamin use (yes/no), menopausal status and hormone use (if NHS or NHS II), family history of type 2 diabetes (yes/no), anti-hypertensive drug use (yes/no), cholesterol-lowering drug use (yes/no), history of hypertension at baseline (yes/no), socioeconomic status, and time-varying body mass index (<21, 21-23, 23-25, 25-27, 27-30, 30-33, 33-35, 35-40, ≥40 kg/m2). All covariates (except race, family history of diabetes, and baseline hypertension) were updated every 2 years.
Model 3 was adjusted for the covariates in model 2 + cumulative average intake of total red meat (serving/d; quintiles), poultry (serving/d; quintiles), fish (serving/d; quintiles), egg (serving/d; quintiles), total dairy (serving/d; quintiles), nuts and legumes (serving/d; quintiles), fruits (serving/d; quintiles), vegetables (serving/d; quintiles), sugar-sweetened beverages (serving/d; quintiles), whole grains (serving /d; quintiles) and refined grains (serving /d; quintiles), and mutual adjustment for different potatoes (serving/d; quintiles).
Abbreviations: NHS, Nurses' Health Study; NHS II, Nurses' Health Study II; HPFS, Health Professionals Follow-up Study

Supplementary Table 19. Associations between 3-serving/week potato intake and risk of diabetes in the NHS, NHS II, and HPFS, stratified by physical activity, BMI, hypertension, and smoking status (Cohort-specific results) [1 2.]

			Total potato		Baked, boiled, mashed potato		French fries	
		**Cases**	**HR (95% CI)**	**P-value**	**HR (95% CI)**	**P-value**	**HR (95% CI)**	**P-value**
**NHS (n=72,712)**								
Age	< 65	4635	1.06 (1.00 to 1.12)	0.06	1.04 (0.98 to 1.11)	0.24	1.20 (1.03 to 1.41)	0.02
	>= 65	4990	1.02 (0.95 to 1.09)	0.64	1.00 (0.93 to 1.08)	0.96	1.22 (0.96 to 1.55)	0.12
	P-interaction		0.23		0.55		0.09	
aHEI score	< median	5541	1.08 (1.02 to 1.13)	0.006	1.05 (0.99 to 1.11)	0.09	1.29 (1.11 to 1.49)	<0.001
	>= median	4084	1.07 (0.99 to 1.16)	0.07	1.00 (0.93 to 1.09)	0.92	2.12 (1.64 to 2.74)	<0.001
	P-interaction		0.08		0.03		0.27	
Physical Activity (METs-hr/Week)	< median	5737	0.99 (0.94 to 1.05)	0.82	0.98 (0.92 to 1.04)	0.43	1.15 (0.98 to 1.37)	0.10
	>= median	3888	1.14 (1.06 to 1.22)	<0.001	1.10 (1.02 to 1.18)	0.01	1.61 (1.29 to 2.01)	<0.001
	P-interaction		0.83		0.70		0.39	
BMI (kg/m^2)	< 25	1411	1.02 (0.91 to 1.15)	0.71	1.02 (0.90 to 1.14)	0.80	1.08 (0.71 to 1.66)	0.72
	25-30	3179	1.02 (0.94 to 1.10)	0.69	0.98 (0.90 to 1.06)	0.55	1.56 (1.21 to 2.00)	<0.001
	>=30	5035	1.05 (0.98 to 1.11)	0.14	1.02 (0.95 to 1.09)	0.56	1.26 (1.06 to 1.50)	<0.001
	P-interaction		0.006		0.05		0.02	
Baseline hypertension	No	6406	1.05 (1.00 to 1.12)	0.07	1.02 (0.96 to 1.08)	0.60	1.40 (1.19 to 1.65)	<0.001
	Yes	3220	1.03 (0.95 to 1.10)	0.48	1.02 (0.95 to 1.10)	0.59	1.08 (0.85 to 1.36)	0.55
	P-interaction		0.91		0.76		0.29	
Smoking	Never	4290	1.05 (0.98 to 1.12)	0.18	1.01 (0.94 to 1.08)	0.83	1.45 (1.19 to 1.77)	<0.001
	Past	4335	1.07 (1.00 to 1.15)	0.05	1.05 (0.97 to 1.13)	0.21	1.31 (1.06 to 1.60)	0.01
	Current	1000	0.93 (0.82 to 1.06)	0.28	0.94 (0.82 to 1.08)	0.42	0.83 (0.55 to 1.24)	0.37
	P-interaction		0.31		0.51		0.09	
**NHS II (n=90,232)**								
Age	< 65	8092	1.02 (0.97 to 1.08)	0.34	1.02 (0.95 to 1.08)	0.63	1.05 (0.95 to 1.16)	0.30
	>= 65	606	1.16 (0.91 to 1.48)	0.23	1.09 (0.81 to 1.45)	0.57	1.38 (0.82 to 2.31)	0.22
	P-interaction		0.34		0.49		0.35	
AHEI score	< median	5254	1.08 (1.02 to 1.14)	0.01	1.02 (0.95 to 1.10)	0.51	1.18 (1.07 to 1.31)	0.001
	>= median	3444	1.08 (0.99 to 1.18)	0.08	1.05 (0.94 to 1.17)	0.37	1.23 (1.00 to 1.52)	0.05

	P-interaction		0.77		0.99		0.23	
Physical Activity (METs-hr/Week)	< median	5807	1.03 (0.97 to 1.10)	0.27	0.99 (0.92 to 1.07)	0.88	1.13 (1.01 to 1.27)	0.03
	>= median	2891	1.04 (0.95 to 1.14)	0.35	1.02 (0.91 to 1.14)	0.74	1.14 (0.94 to 1.38)	0.19
	P-interaction		0.43		0.38		0.57	
BMI (kg/m^2)	< 25	587	1.12 (0.92 to 1.37)	0.24	1.02 (0.80 to 1.30)	0.87	1.55 (1.03 to 2.34)	0.04
	25-30	1763	0.97 (0.86 to 1.10)	0.66	0.93 (0.80 to 1.07)	0.32	1.10 (0.86 to 1.41)	0.46
	>=30	6348	1.07 (1.01 to 1.13)	0.02	1.02 (0.95 to 1.10)	0.54	1.18 (1.06 to 1.31)	0.002
	P-interaction		<0.001		<0.001		0.02	
Baseline hypertension	No	7241	1.05 (0.99 to 1.11)	0.08	1.02 (0.95 to 1.09)	0.62	1.14 (1.02 to 1.27)	0.01
	Yes	1457	0.99 (0.88 to 1.12)	0.91	0.95 (0.82 to 1.10)	0.48	1.09 (0.87 to 1.37)	0.45
	P-interaction		0.79		0.75		0.95	
Smoking	Never	5503	1.05 (0.98 to 1.11)	0.15	1.01 (0.93 to 1.09)	0.86	1.15 (1.02 to 1.30)	0.02
	Past	2419	1.01 (0.91 to 1.11)	0.89	0.98 (0.87 to 1.11)	0.77	1.08 (0.89 to 1.32)	0.44
	Current	776	1.08 (0.92 to 1.26)	0.35	1.05 (0.86 to 1.28)	0.65	1.17 (0.89 to 1.56)	0.26
	P-interaction		0.06		0.17		0.11	
**HPFS (n=42,163)**								
Age	< 65	1822	1.07 (0.99 to 1.17)	0.09	1.05 (0.95 to 1.16)	0.34	1.13 (0.98 to 1.32)	0.10
	>= 65	2154	1.02 (0.93 to 1.11)	0.71	0.99 (0.90 to 1.09)	0.82	1.17 (0.95 to 1.44)	0.14
	P-interaction		0.76		0.66		0.60	
aHEI score	< median	2284	1.03 (0.96 to 1.11)	0.45	0.97 (0.89 to 1.06)	0.51	1.20 (1.04 to 1.38)	0.01
	>= median	1692	1.19 (1.09 to 1.30)	<0.001	1.11 (1.00 to 1.23)	0.05	1.68 (1.39 to 2.03)	<0.001
	P-interaction		0.80		0.95		0.83	
Physical Activity (METs-hr/Week)	< median	2339	1.02 (0.95 to 1.10)	0.61	0.98 (0.90 to 1.08)	0.73	1.15 (0.98 to 1.34)	0.09
	>= median	1637	1.12 (1.02 to 1.23)	0.01	1.05 (0.94 to 1.17)	0.37	1.44 (1.18 to 1.74)	<0.001
	P-interaction		0.99		0.79		0.49	
BMI (kg/m^2)	< 25	681	1.04 (0.91 to 1.20)	0.53	0.96 (0.82 to 1.13)	0.61	1.50 (1.10 to 2.04)	0.009
	25-30	1920	1.03 (0.95 to 1.12)	0.50	0.97 (0.88 to 1.07)	0.57	1.28 (1.07 to 1.53)	0.007
	>=30	1375	1.07 (0.97 to 1.19)	0.18	1.07 (0.94 to 1.21)	0.30	1.10 (0.90 to 1.35)	0.36
	P-interaction		0.01		0.04		0.25	
Baseline hypertension	No	2855	1.01 (0.94 to 1.09)	0.78	0.98 (0.90 to 1.06)	0.61	1.13 (0.98 to 1.32)	0.09
	Yes	1121	1.17 (1.05 to 1.29)	0.003	1.08 (0.95 to 1.22)	0.25	1.55 (1.24 to 1.94)	<0.001

	P-interaction		0.04		0.28		0.01	
Smoking	Never	1978	1.07 (0.98 to 1.16)	0.14	0.98 (0.89 to 1.09)	0.77	1.35 (1.14 to 1.60)	<0.001
	Past	1790	1.04 (0.96 to 1.14)	0.34	1.03 (0.93 to 1.14)	0.57	1.12 (0.92 to 1.37)	0.27
	Current	208	1.04 (0.84 to 1.29)	0.73	1.00 (0.77 to 1.31)	0.98	1.17 (0.75 to 1.84)	0.49
	P-interaction		0.72		0.61		0.50	

Abbreviations: NHS, Nurses' Health Study; NHS II, Nurses' Health Study II; HPFS, Health Professionals Follow-up Study; BMI, body mass index

[1] Dietary intake were cumulative averages from the baseline FFQ to the start of each 4-year follow-up interval.

[2] The model was stratified by age (months) and calendar time (two-year interval), and adjusted for total energy intake, race/ethnicity (white adults, non-white adults), smoking status (never, past, current: 1-14 cigs/day, current: >15-24 cigs/day, current: >24 cigs/day), alcohol intake (non-alcohol drinker, 0-4.9 grams/day, 5-9.9 grams/day, 10-14.9 grams/day, 15-29.9 grams/day, >30 grams/day), physical activity (<3, 3-9, 9-18, 18-27, ≥ 27 METs-hr/week), multivitamin use, menopausal status and hormone use (if NHS or NHS II), family history of type 2 diabetes, anti-hypertensive drug use, cholesterol-lowering drug use, history of hypertension, socioeconomic status, time-varying body mass index (<21, 21-23, 23-25, 25-27, 27-30, 30-33, 33-35, 35-40, ≥40 kg/m^2) and dietary covariates intakes (including total red meat, poultry, fish, egg, total dairy, nuts and legumes, fruits, vegetables, sugar-sweetened beverages, whole grain, and refined grain, and mutual adjustment for different potatoes.

Supplementary Table 20. Associations between every 3-serving/week increment in potato intakes and risk of diabetes in the NHS, NHS II, and HPFS by latency period (cohort-specific results) [1,2]

	**0-4 yrs**		**4-8 yrs**		**8-12 yrs**		**12-16 yrs**		**16-20 yrs**		**20-24 yrs**		**24-28 yrs**	
	**HR (95% CI)**	**P-value**	**HR (95% CI)**	**P-value**	**HR (95% CI)**	**P-value**	**HR (95% CI)**	**P-value**	**HR (95% CI)**	**P-value**	**HR (95% CI)**	**P-value**	**HR (95% CI)**	**P-value**
**NHS (n=72,712)**														
**Cases**	9,625		8,837		7,919		6,671		5,077		3,251		1,562	
**Total potato**	1.03 (1.00 to 1.07)	0.05	1.01 (0.98 to 1.05)	0.46	1.01 (0.97 to 1.05)	0.56	1.05 (1.01 to 1.09)	0.01	1.04 (1.00 to 1.08)	0.05	1.02 (0.96 to 1.07)	0.53	0.96 (0.88 to 1.05)	0.38
**Baked, boiled, mashed potato**	1.01 (0.98 to 1.05)	0.45	1.00 (0.96 to 1.04)	0.99	0.99 (0.96 to 1.03)	0.76	1.04 (0.99 to 1.08)	0.06	1.03 (0.98 to 1.07)	0.24	1.00 (0.94 to 1.06)	0.95	0.94 (0.86 to 1.03)	0.17
**French fries**	1.24 (1.11 to 1.38)	<0.001	1.14 (1.02 to 1.28)	0.02	1.18 (1.05 to 1.32)	0.003	1.17 (1.04 to 1.32)	0.008	1.19 (1.04 to 1.35)	0.009	1.16 (0.99 to 1.36)	0.08	1.15 (0.91 to 1.44)	0.23
**NHS II (n=90,232)**														
**Cases**	8,698		8,274		7,474		6,163		4,420		2,612		1,191	
**Total potato**	1.03 (0.99, 1.06)	0.18	1.03 (0.99 to 1.07)	0.07	1.05 (1.01 to 1.09)	0.009	1.06 (1.01 to 1.10)	0.008	1.06 (1.01 to 1.12)	0.009	1.09 (1.02 to 1.16)	0.006	1.10 (1.01 to 1.21)	0.04
**Baked, boiled, mashed potato**	1.00 (0.96, 1.05)	0.79	0.99 (0.95 to 1.04)	0.86	1.03 (0.99 to 1.08)	0.16	1.05 (1.00 to 1.11)	0.03	1.05 (0.99 to 1.11)	0.08	1.07 (0.99 to 1.16)	0.06	1.09 (0.97 to 1.22)	0.13
**French fries**	1.07 (0.99, 1.16)	0.09	1.14 (1.06 to 1.23)	<0.001	1.11 (1.03 to 1.19)	0.008	1.06 (0.97 to 1.15)	0.16	1.10 (1.00 to 1.21)	0.05	1.14 (1.01 to 1.28)	0.03	1.13 (0.94 to 1.35)	0.20
**HPFS (n=42,163)**														
**Cases**	3.976		3,483		2,949		2,329		1,593		922		437	
**Total potato**	1.02 (0.98, 1.07)	0.33	1.01 (0.96 to 1.06)	0.64	1.06 (1.01 to 1.12)	0.01	1.05 (0.99 to 1.11)	0.07	1.01 (0.94 to 1.09)	0.76	1.00 (0.91 to 1.10)	0.96	0.99 (0.85 to 1.15)	0.90
**Baked, boiled, mashed potato**	0.99 (0.94, 1.05)	0.91	0.97 (0.92 to 1.03)	0.39	1.05 (0.99 to 1.11)	0.11	1.02 (0.95 to 1.09)	0.56	0.99 (0.91 to 1.08)	0.85	0.98 (0.87 to 1.10)	0.69	0.93 (0.77 to 1.12)	0.46
**French fries**	1.12 (1.02, 1.27)	0.02	1.15 (1.04 to 1.28)	0.01	1.12 (1.00 to 1.25)	0.04	1.17 (1.04 to 1.31)	0.01	1.07 (0.92 to 1.24)	0.32	1.08 (0.91 to 1.29)	0.38	1.11 (0.86 to 1.43)	0.42

Abbreviations: NHS, Nurses' Health Study; NHS II, Nurses' Health Study II; HPFS, Health Professionals Follow-up Study

[2] The model was stratified by age (months) and calendar time (two-year interval), and adjusted for total energy intake, race/ethnicity (white adults, non-white adults), smoking status (never, past, current: 1-14 cigs/day, current: >15-24 cigs/day, current: >24 cigs/day), alcohol intake (non-alcohol drinker, 0-4.9 grams/day, 5-9.9 grams/day, 10-14.9 grams/day, 15-29.9 grams/day, >30 grams/day), physical activity (<3, 3-9, 9-18, 18-27, ≥ 27 METs-hr/week), multivitamin use, menopausal status and hormone use (if NHS or NHS II), family history of type 2 diabetes, anti-hypertensive drug use, cholesterol-lowering drug use, history of hypertension, socioeconomic status, time-varying body mass index (<21, 21-23, 23-25, 25-27, 27-30, 30-33, 33-35, 35-40, ≥40 kg/m^2) and dietary covariates intakes (including total red meat, poultry, fish, egg, total dairy, nuts and legumes, fruits, vegetables, sugar-sweetened beverages, whole grain, and refined grain, and mutual adjustment for different potatoes.

Supplementary Table 21. Associations between substituting 3-serving/week of other foods for three-serving/week of potatoes intakes and the risk of type 2 diabetes in the NHS, NHS II, and HPFS (Cohort-specific results)

	**NHS (n=72,712)**		**NHS II (n=90,232)**		**HPFS (n=42,163)**	
	**HR (95% CI)**	**P-value**	**HR (95% CI)**	**HR (95% CI)**	**P-value**	**P-value**
**Cases/ Person-years**	9,625/ 1,881,139		8,698/ 2,308,071		3,976/ 986,291	
**Total potato**						
Whole grains	0.91 (0.87 to 0.96)	<0.001	0.93 (0.88 to 0.98)	0.01	0.92 (0.86 to 0.98)	0.008
Refined grains	0.97 (0.93 to 1.02)	0.16	0.98 (0.93 to 1.03)	0.47	0.97 (0.91 to 1.03)	0.27
Non-starchy vegetables	0.94 (0.90 to 0.99)	0.01	0.95 (0.90 to 1.00)	0.07	0.96 (0.90 to 1.02)	0.18
Starchy vegetables	0.97 (0.89 to 1.06)	0.52	1.02 (0.93 to 1.12)	0.63	1.04 (0.93 to 1.16)	0.44
Legumes	0.96 (0.85 to 1.09)	0.55	0.93 (0.83 to 1.05)	0.26	0.88 (0.76 to 1.02)	0.09
White rice	1.21 (1.13 to 1.31)	<0.001	1.16 (1.09 to 1.25)	<0.001	1.07 (0.98 to 1.16)	0.11
Brown rice	0.99 (0.86 to 1.14)	0.87	1.00 (0.87 to 1.14)	0.99	0.95 (0.81 to 1.11)	0.53
**Baked, boiled, mashed potato**						
Whole grains	0.94 (0.89 to 0.99)	0.01	0.97 (0.91 to 1.04)	0.36	0.96 (0.89 to 1.04)	0.34
Refined grains	0.99 (0.95 to 1.05)	0.70	1.02 (0.96 to 1.09)	0.53	1.02 (0.95 to 1.09)	0.67
Non-starchy vegetables	0.97 (0.92 to 1.02)	0.20	0.99 (0.93 to 1.06)	0.82	1.01 (0.94 to 1.08)	0.80
Starchy vegetables	0.99 (0.91 to 1.09)	0.93	1.07 (0.97 to 1.18)	0.20	1.10 (0.98 to 1.23)	0.11
Legumes	0.99 (0.87 to 1.12)	0.87	0.97 (0.86 to 1.10)	0.63	0.92 (0.79 to 1.07)	0.27
White rice	1.25 (1.16 to 1.35)	<0.001	1.21 (1.12 to 1.30)	<0.001	1.12 (1.02 to 1.22)	0.01
Brown rice	1.01 (0.88 to 1.17)	0.85	1.04 (0.90 to 1.19)	0.61	1.00 (0.85 to 1.17)	0.99
**French fries**						
Whole grains	0.74 (0.64 to 0.84)	<0.001	0.84 (0.76 to 0.94)	0.001	0.78 (0.69 to 0.88)	<0.001
Refined grains	0.78 (0.68 to 0.89)	<0.001	0.89 (0.80 to 0.98)	0.02	0.82 (0.73 to 0.93)	0.002
Non-starchy vegetables	0.76 (0.66 to 0.87)	<0.001	0.87 (0.78 to 0.96)	0.004	0.82 (0.72 to 0.92)	0.001
Starchy vegetables	0.78 (0.67 to 0.91)	0.002	0.93 (0.82 to 1.05)	0.26	0.90 (0.76 to 1.04)	0.15

Legumes	0.77 (0.65 to 0.93)	0.006	0.85 (0.73 to 0.98)	0.02	0.75 (0.62 to 0.90)	0.002
White rice	0.97 (0.84 to 1.13)	0.73	1.06 (0.95 to 1.18)	0.27	0.92 (0.80 to 1.05)	0.21
Brown rice	0.79 (0.65 to 0.96)	0.02	0.91 (0.77 to 1.07)	0.26	0.81 (0.66 to 0.97)	0.02

Abbreviations: NHS, Nurses' Health Study; NHS II, Nurses' Health Study II; HPFS, Health Professionals Follow-up Study

1 Dietary intake were cumulative averages from the baseline FFQ to the start of each 4-year follow-up interval.

2 The model was stratified by age (months) and calendar time (two-year interval), and adjusted for total energy intake, race/ethnicity (white adults, non-white adults), smoking status (never, past, current: 1-14 cigs/day, current: >15-24 cigs/day, current: >24 cigs/day), alcohol intake (non-alcohol drinker, 0-4.9 grams/day, 5-9.9 grams/day, 10-14.9 grams/day, 15-29.9 grams/day, >30 grams/day), physical activity (<3, 3-9, 9-18, 18-27, ≥ 27 METs-hr/week), multivitamin use, menopausal status and hormone use (in NHS or NHS II), family history of type 2 diabetes, anti-hypertensive drug use, cholesterol-lowering drug use, history of hypertension, socioeconomic status, time-varying body mass index (<21, 21-23, 23-25, 25-27, 27-30, 30-33, 33-35, 35-40, ≥40 kg/m2) and dietary covariates intakes (including total red meat, poultry, fish, egg, total dairy, nuts and legumes, fruits, vegetables, sugar-sweetened beverages, whole grain, and refined grain, and mutual adjustment for different potatoes (serving/d; quintiles), excluding the foods subject for substitutions.

Supplementary Table 22. List of excluded studies and exclusion reason	
**Exclusion reason**	**Reference number**
Not relevant exposure	1-12
Not relevant data	13-16
Conference abstract	17-19
Duplicated within the same cohort	20-24
Review article	25

Reference List:

1. Fung, T.T., et al., *Dietary patterns, meat intake, and the risk of type 2 diabetes in women.* Arch Intern Med, 2004. 164(20): p. 2235-40.
2. Montonen, J., et al., *Dietary patterns and the incidence of type 2 diabetes.* Am J Epidemiol, 2005. 161(3): p. 219-27.
3. Liese, A.D., et al., *Food intake patterns associated with incident type 2 diabetes: the Insulin Resistance Atherosclerosis Study.* Diabetes Care, 2009. 32(2): p. 263-8.
4. Odegaard, A.O., et al., *Risk of incident type 2 diabetes according to frequency of Western-style fast food intake in an eastern country.* Diabetes, 2011. 60: p. A377.
5. Langsetmo, L., et al., *Dietary patterns in men and women aged 25 years and older: Relationship with body mass index, 25-hydroxyvitamin d levels, fasting glucose, and risk of diabetes mellitus.* Journal of Bone and Mineral Research, 2012. 27.
6. Morimoto, A., et al., *Effects of healthy dietary pattern and other lifestyle factors on incidence of diabetes in a rural Japanese population.* Asia Pac J Clin Nutr, 2012. 21(4): p. 601-8.
7. Mursu, J., et al., *Intake of fruit, berries, and vegetables and risk of type 2 diabetes in Finnish men: the Kuopio Ischaemic Heart Disease Risk Factor Study.* Am J Clin Nutr, 2014. 99(2): p. 328-33.
8. Pastorino, S., et al., *A high-fat, high-glycaemic index, low-fibre dietary pattern is prospectively associated with type 2 diabetes in a British birth cohort.* Br J Nutr, 2016. 115(9): p. 1632-42.
9. Becerra-Tomás, N., et al., *Legume consumption is inversely associated with type 2 diabetes incidence in adults: A prospective assessment from the PREDIMED study.* Clin Nutr, 2018. 37(3): p. 906-913.
10. Partula, V., et al., *Associations between consumption of dietary fibers and the risk of cardiovascular diseases, cancers, type 2 diabetes, and mortality in the prospective NutriNet-Santé cohort.* Am J Clin Nutr, 2020. 112(1): p. 195-207.
11. Huang, M., et al., *Pasta meal intake in relation to risks of type 2 diabetes and atherosclerotic cardiovascular disease in postmenopausal women : findings from the Women's Health Initiative.* BMJ Nutr Prev Health, 2021. 4(1): p. 195-205.

12. Yu, Z., et al., *Association of fruit and vegetable color with incident diabetes and cardiometabolic risk biomarkers in the United States Hispanic/Latino population.* Nutr Diabetes, 2022. 12(1): p. 18.
13. Feskens, E.J., et al., *Dietary factors determining diabetes and impaired glucose tolerance. A 20-year follow-up of the Finnish and Dutch cohorts of the Seven Countries Study.* Diabetes Care, 1995. 18(8): p. 1104-12.
14. Cruijsen, E., Indyk, I. M., Simon, A. W., Busstra, M. C., & Geleijnse, J. M. (2022). Potato consumption and risk of cardiovascular mortality and type 2 diabetes after myocardial infarction: A prospective analysis in the alpha omega cohort. Frontiers in Nutrition, 8, 813851.
15. Takahashi, K., et al., *Effects of total and green vegetable intakes on glycated hemoglobin A1c and triglycerides in elderly patients with type 2 diabetes mellitus: The Japanese Elderly Intervention Trial.* Geriatrics & Gerontology International, 2012. 12: p. 50-58.
16. Åsli, L.A., et al., *What characterises women who eat potatoes? A cross-sectional study among 74,208 women in the Norwegian Women and Cancer cohort.* Food Nutr Res, 2015. 59: p. 25703.
17. Pastorino, S., et al., *A high fat, high gi, low fibre dietary pattern is associated with increased type 2 diabetes risk in british adults.* Annals of Nutrition and Metabolism, 2013. 63: p. 976.
18. Cruijsen, E., et al., *Potato Consumption And Risk Of Diabetes And Cardiovascular Mortality In The Alpha Omega Cohort.* Circulation, 2021. 143.
19. Duan, M.F., et al., *Lifestyle behaviour patterns and incident type 2 diabetes in the Dutch Lifelines cohort study.* Diabetologia, 2022. 65: p. S167.
20. van Dam, R.M., et al., *Dietary patterns and risk for type 2 diabetes mellitus in U.S. men.* Ann Intern Med, 2002. 136(3): p. 201-9.
21. Halton, T.L., et al., *Potato and french fry consumption and risk of type 2 diabetes in women.* Am J Clin Nutr, 2006. 83(2): p. 284-90.
22. Satija, A., et al., *A prospective study of the association between plant-based dietary patterns and incident type 2 diabetes in women.* Circulation, 2015. 131.
23. Muraki, I., et al., *Potato Consumption and Risk of Type 2 Diabetes: Results From Three Prospective Cohort Studies.* Diabetes Care, 2016. 39(3): p. 376-84.
24. Satija, A., et al., *Plant-Based Dietary Patterns and Incidence of Type 2 Diabetes in US Men and Women: Results from Three Prospective Cohort Studies.* PLoS Med, 2016. 13(6): p. e1002039.
25. Ooi, C.P. and S.C. Loke, *Sweet potato for type 2 diabetes mellitus.* Cochrane Database Syst Rev, 2012(2): p. Cd009128.

**Supplementary Table 23.** Characteristics of studies included in the meta-analysis.[1]												
**Author, year**	**Study Population**	**Country**	**Study size, Age range,**	**Follow-up period (years)**	**Dietary assessment**	**Number of Cases**	**Ascertainment of Cases**	**Statistical Model**	**Exposure**	**Categories of Exposure**	**Relative Risks (95% CI)**	**Adjustment for confounders**
1. Hodge et al., 2004	Melbourne Collaborative Cohort Study (MCCS)	Australia	31,641 participants, 27–75 years Men and women	4	Self-administered FFQ, 121 items at baseline	365	Self-reported and confirmed by a doctor	Multivariable logistic regression	Potato	Increase of one time/wk<br><2.0 times/wk<br>2.0-3.9<br>4.0-6.4<br>≥6.5	0.99 (0.94 to 1.04)<br>1.00<br>0.84 (0.63 to 1.12)<br>0.82 (0.60 to 1.12)<br>0.98 (0.70 to 1.37)	age, sex, country of birth, physical activity, family history of diabetes, alcohol intake, education level, weight change in the last 5 years, and energy intake, BMI, WHR
2. Liu et al., 2004	Women's Health Study (WHS)	USA	38,018 participants Age ≥45 years Women	8.8	Validated semiquantitative FFQ, 131 items at baseline	1,614	Self-reported /ADA criteria	Cox proportional hazards	Potatoes	0.13 serv/d<br>0.28<br>0.43<br>0.56<br>0.93	1.00<br>1.03 (0.87 to 1.22)<br>0.97 (0.79 to 1.19)<br>0.96 (0.81 to 1.13)<br>1.02 (0.86 to 1.22)	age, smoking, total calories, alcohol use, BMI, exercise, history of hypertension, history of high cholesterol, and family history of diabetes
3. Montonenet al., 2005	Finnish Mobile Clinic Health Examination Survey (FMCHES)	Finland	4,304 participants, age 40-69, men and women	23	Dietary history interview, > 100 food items at baseline	383	Report from Social Insurance Institution's nationwide register	Cox proportional hazards	Potato	<132 g/d<br>132-196<br>197-283<br>>283	1.00<br>1.09 (0.82 to 1.46)<br>1.27 (0.94 to 1.72)<br>1.42 (1.02 to 1.98)	Age, sex, BMI, energy intake, smoking, family, and geographic area
4. von Ruestenet al. 2013	EPIC-Potsdam	Germany	23,531 participants, Age 35–65, men and women	8	self-administered FFQ, 148 food items at baseline	837	Self-report, validation by treating physician	Cox proportional hazards	Non-fried Potatoes<br><br>Fried potato	100 g/d increase<br><br>100 g/d increase	1.03 (0.87 to 1.21)<br><br>1.16 (0.70 to 1.94)	Age, sex, smoking status, pack-years of smoking, alcohol consumption, leisure-time physical activity, BMI, waist-to-hip ratio, prevalent hypertension at baseline, history of high blood lipid levels at baseline, education, vitamin supplementation, non-consumption of the respective food group

												and total energy intake, and intakes of 44 other foods.
5. Chen et al. 2018	Singapore Chinese Health Study (SCHS)	Singapore	45,411 participants, age 45-74 men and women	10.9	Validated semiquantitative FFQ, 165 items at baseline	5,207	Self-reported, validated by linkage with a nationwide hospital-based discharge database and supplemntary questionnaire	Cox proportional hazards	Potatoes	0.023 g/d<br>1.802<br>3.604<br>5.876<br>11.517	1.00<br>1.02 (0.94 to 1.11)<br>0.97 (0.89 to 1.06)<br>1.02 (0.94 to 1.11)<br>0.95 (0.87 to 1.04)	Age, sex, dialect group, year of the baseline interview, energy intake, physical activity, education, smoking, alcohol, soft drink, coffee, energy-adjusted intakes of red meat, poultry, fish, nuts and seeds, soya products, wholegrains, BMI, history of hypertension
6. Farhadnejad et al. 2018	Tehran Lipid and Glucose Study (TLGS)	Iran	1,981 participants, age 18-75, men and women	6	Validated FFQ, 168 items at baseline	132	Fasting plasma glucose levels (ADA criteria)	Multivariable logistic regression	Total potato<br><br><br><br>Boiled potato<br><br><br><br>Fried potato	7.30 g/d<br>16.05<br>29.22<br>55.50<br>2.42 g/d<br>10.38<br>20.76<br>36.3<br>1.30 g/d<br>4.66<br>10.33<br>25.71	1.00<br>0.60 (0.34 to 1.01)<br>0.75 (0.45 to 1.26)<br>0.46 (0.25 to 0.84)<br>1.00<br>0.65 (0.39 to 1.08)<br>0.74 (0.43 to 1.28)<br>0.47 (0.26 to 0.85)<br>1.00<br>0.82 (0.50 to 1.35)<br>0.60 (0.35 to 1.03)<br>0.50 (0.25 to 1.07)	Age, sex, BMI, physical activity, smoking, family history of diabetes, hypertension, serum triglycerides, high-density lipoprotein cholesterol, daily intakes of energy, saturated fat (g/d) and food groups intake (g/d), including fruit, whole grains, vegetables, nuts and legumes.
7. Yiannakou et al., 2022	Framingham Offspring Study cohort	USA	2,523 participants, ≥30 years, men and women	16	3-d food records	538	Fasting glucose concentration ≥126 mg/dL, current use of glucose-lowering medications, the use of insulin injection, or self-	Cox proportional hazards	Total potato<br><br><br><br>Fried potato<br><br><br>Non-fried potato	<1 (cup-eq per week)<br>1–<2<br>2–<4<br>≥4<br><1 (cup-eq per week)<br>1–<2<br>≥2<br><1 (cup-eq per week)<br>1–<2<br>≥2<br><br>Potato chips: One cup-equivalent of potato	1.00<br>0.83 (0.69 to 1.00)<br>0.87 (0.73 to 1.03)<br>0.97 (0.81 to 1.15)<br>1.00<br>1.10 (0.94 to 1.30)<br>0.95 (0.80 to 1.12)<br>1.00<br>0.89 (0.76 to 1.05)<br>0.99 (0.86 to 1.13)	sex, age, education, cigarette smoking, updated waist-to-height ratio and hip circumference, fruit and non-starchy vegetables, and red meat intakes. Models for fried potato intake were also adjusted for non-fried potatoes; those for non-fried

							reported physician's diagnosis			chips is defined as 2 ounces or 56.7 grams.		potatoes were adjusted for fried potatoes
8. Pokharel et al. 2023	Danish Diet, Cancer and Health cohort	Denmark	54,793 participants, age 50-64, Men and women	16.3	192-item food frequency questionnaire (FFQ) at baseline	7,695	National Diabetes Register criteria	Cox proportional hazards	Non-fried potato<br><br><br><br><br><br>Boiled potato<br><br><br><br><br>Mashed potato<br><br><br><br><br>Roasted potatoes<br><br><br><br><br><br>Fried potato	52 g/d<br>87<br>125<br>163<br>256<br>21 g/d<br>54<br>96<br>125<br>225<br>6 g/d<br>8<br>12<br>16<br>39<br>2 g/d<br>5<br>12<br>16<br>27<br>0<br>2<br>2.5<br>4<br>8	1.00<br>1.01 (0.96 to 1.06)<br>1.03 (0.97 to 1.09)<br>1.05 (0.99 to 1.11)<br>1.04 (0.98 to 1.12)<br>1.00<br>1.01 (0.96 to 1.07)<br>1.05 (0.99 to 1.11)<br>1.06 (1.00 to 1.13)<br>1.03 (0.96 to 1.10)<br>1.00<br>1.00 (0.99 to 1.02)<br>1.02 (0.99 to 1.04)<br>1.03 (0.99 to 1.07)<br>1.07 (1.00 to 1.14)<br>1.00<br>1.00 (0.97 to 1.03)<br>0.99 (0.93 to 1.04)<br>0.98 (0.93 to 1.03)<br>0.97 (0.90 to 1.03)<br>1.00<br>1.07 (1.00 to 1.16)<br>1.08 (1.00 to 1.17)<br>1.08 (1.00 to 1.17)<br>1.08 (0.99 to 1.18)	sex, age, physical activity, smoking status, alcohol status, education level, hormone replacement therapy, intake (g/day) of whole grains, refined grains, red meat, processed meat, poultry, fish, dairy, vegetable oil, animal fats, sugar and confectionery, soft drinks, fruit, tea, and coffee and intake of other exposures, except the exposure of interest.
9. Wan et al. 2023	UK Biobank	United Kingdom	174,665 participants Age 37–73, Men and women	11.4	24-hour dietary assessment method near 200 items, 5 times	4,059	hospital inpatient records	Cox proportional hazards	Total potato<br><br><br><br><br>Boiled potato<br><br><br><br><br>Mashed potato<br><br><br><br><br>Fried potato	1 SD increment<br>0 servings/d<br>0-1<br>1-2<br>≥2<br>1 SD increment<br>0 servings/d<br>0-1<br>1-2<br>≥2<br>1 SD increment<br>0 servings/d<br>0-1<br>1-2<br>≥2<br>1 SD increment<br>0 servings/d	1.07 (1.04 to 1.10)<br>1.00<br>0.99 (0.91 to 1.08)<br>1.09 (1.00 to 1.19)<br>1.30 (1.15 to 1.47)<br>1.02 (0.99 to 1.05)<br>1.00<br>1.02 (0.94 to 1.10)<br>1.04 (0.94 to 1.16)<br>1.10 (0.87 to 1.40)<br>1.05 (1.02 to 1.08)<br>1.00<br>1.01 (0.92 to 1.10)<br>1.20 (1.03 to 1.39)<br>1.74 (1.09 to 2.77)<br>1.02 (0.99 to 1.05)<br>1.00	Model 3: age, sex, race, BMI, education, Townsend deprivation index, household income, smoking, alcohol consumption, physical activity, history of hypertension, history of high cholesterol, family history of diabetes, vitamin supplement use, mineral supplement use, aspirin use, and lipid-lowering medication use, total

									Baked, boiled, or mashed	0-1<br>1-2<br>≥2	1.03 (0.95 to 1.11)<br>1.03 (0.93 to 1.14)<br>1.05 (0.83 to 1.33)	energy intake, for vegetable intake, fruit intake, whole grains intake, processed red meat intake, fish intake, poultry intake, sugar sweetened beverages intake, total energy intake, and remaining different processed potatoes (boiled/baked potato, mashed potato, and fried potato)
10. Pokarel et al, 2024	AusDiab Cohort	Australia	5,107	12	74-item Food Frequency Questionnaire (FFQ), at baseline	342	FPG level of ≥7.0 mmol/L (≥126 mg/dL), 2-hour PLG level of ≥11.1 mmol/L (≥200 mg/dL), or treatment with insulin or oral hypoglycemic agents	Cox proportional hazards	Total potato<br><br><br><br><br><br><br>Potato fries/chips	2–10 g/day<br>19-28<br>38-51<br>68-102<br><br><br><br>1-4 g/day<br>7-10<br>14-21<br>31-58	1.00<br>0.90 (0.77 to 1.05)<br>0.84 (0.65 to 1.08)<br>0.85 (0.65 to 1.13)<br><br><br><br>1.00<br>0.97 (0.87 to 1.10)<br>0.94 (0.73 to 1.21)<br>0.88 (0.63 to 1.23)	Model 3: age and sex, age, sex, physical activity level, education level, Socio-Economic Indexes for Areas, income, smoking status, parental history of diabetes, alcohol intake (g/day), intakes (g/day) of wholegrains, refined grains, red meat, processed meat, poultry, eggs, fish, dairy, fat, fruits, nuts, miscellaneous and discretionary foods, body mass index and self-reported prevalence of hypertension, high cholesterol and cardiovascular disease
11. Mousavi et al, (current)	Nurses' Health Study (NHS)	USA	72,712 participants, Age 30–55, women	27	Semi-quantitative FFQ, every 4 years.	9625	Self-report and validation with supplementary questionnaire	Cox proportional hazards	Total potato	3-serving/ week<br><br><1 serving/week<br>1<br>2-4<br>5-6<br>≥7	1.05 (1.00 to 1.09)<br><br>1.00<br>1.06 (0.93 to 1.20)<br>1.06 (0.95 to 1.20)<br>1.12 (0.99 to 1.27)<br>1.14 (0.98 to 1.33)<br><br>1.02 (0.97 to 1.07)	age, total energy intake, race/ethnicity, smoking status, alcohol intake, physical activity, multivitamin use, menopausal status and hormone use, family history of type 2

									Baked, boiled, or mashed<br><br><br><br><br><br>French fries	3-serving/ week<br><br><1 serving/week<br>1<br>2-4<br>≥5<br><br>3-serving/ week<br><br>Almost never<br>1-3/month<br>2-4 serving/week<br>5-6<br>≥7	1.00<br>0.94 (0.86 to 1.04)<br>0.93 (0.85 to 1.01)<br>0.96 (0.87 to 1.06)<br><br>1.29 (1.13 to 1.47)<br><br>1.00<br>1.12 (1.06 to 1.19)<br>1.14 (1.06 to 1.23)<br>1.21 (1.10 to 1.34)<br>0.89 (0.54 to 1.46)	diabetes, anti-hypertensive drug use, cholesterol-lowering drug use, history of hypertension at baseline, socioeconomic status, and body mass index, total red meat, poultry, fish, egg, total dairy, nuts and legumes, fruits, vegetables, SSB, whole grains, and refined grains, baked, boiled or mashed potatoes (for French fries), and French fries (for baked, boiled, or mashed potatoes).
12. Mousavi et al, (current)	Nurses' Health Study (NHS) II	USA	90,232 participants, Age 25–42, women	25.6	Semi-quantitative FFQ, every 4 years.	8698	Self-report and validation with suppleme ntary questionn aire	Cox proportional hazards	Total potato<br><br><br><br><br><br><br>Baked, boiled, or mashed<br><br><br><br><br>French fries	3-serving/ week<br><br><1 serving/week<br>1<br>2-4<br>5-6<br>≥7<br><br>3-serving/ week<br><br><1 serving/week<br>1<br>2-4<br>≥5<br><br>3-serving/ week<br><br>Almost never<br>1-3/month<br>2-4 serving/week<br>5-6<br>≥7	1.04 (0.99 to 1.09)<br><br>1.00<br>0.94 (0.82 to 1.07)<br>0.97 (0.85 to 1.09)<br>0.98 (0.86 to 1.12)<br>1.13 (0.96 to 1.33)<br><br>1.00 (0.94 to 1.07)<br><br>1.00<br>1.00 (0.92 to 1.08)<br>1.01 (0.93 to 1.09)<br>1.02 (0.92 to 1.13)<br><br>1.13 (1.03 to 1.25)<br><br>1.00<br>1.02 (0.93 to 1.11)<br>1.04 (0.95 to 1.15)<br>1.05 (0.95 to 1.16)<br>1.15 (0.92 to 1.45)	age, total energy intake, race/ethnicity, smoking status, alcohol intake, physical activity, multivitamin use, menopausal status and hormone use, family history of type 2 diabetes, anti-hypertensive drug use, cholesterol-lowering drug use, history of hypertension at baseline, socioeconomic status, and body mass index, total red meat, poultry, fish, egg, total dairy, nuts and legumes, fruits, vegetables, SSB, whole grains, and refined grains, baked, boiled or mashed potatoes (for French fries), and

												French fries (for baked, boiled, or mashed potatoes).
13. Mousavi et al, (current)	Health Professionals' Follow-up Study (HPFS)	USA	42,163, participants, Age 40–75, men	27	Semi-quantitative FFQ, every 4 years.	3976	Self-report and validation with supplementary questionnaire	Cox proportional hazards	Total potato<br><br><br><br><br><br><br><br>Baked, boiled, or mashed<br><br><br><br><br>French fries	3-serving/ week<br><br><1 serving/week<br>1<br>2-4<br>5-6<br>≥7<br><br>3-serving/ week<br><br><1 serving/week<br>1<br>2-4<br>≥5<br><br>3-serving/ week<br><br>Almost never<br>1-3/month<br>2-4 serving/week<br>5-6<br>≥7	1.06 (1.00 to 1.12)<br><br>1.00<br>0.91 (0.75 to 1.09)<br>0.97 (0.83 to 1.15)<br>1.01 (0.85 to 1.20)<br>1.10 (0.90 to 1.35)<br><br>1.01 (0.94 to 1.08)<br><br>1.00<br>0.96 (0.85 to 1.08)<br>0.91 (0.81 to 1.02)<br>1.00 (0.87 to 1.14)<br><br>1.25 (1.10 to 1.41)<br><br>1.00<br>1.06 (0.96 to 1.18)<br>1.18 (1.05 to 1.32)<br>1.22 (1.08 to 1.39)<br>1.60 (1.23 to 2.08)	age, total energy intake, race/ethnicity, smoking status, alcohol intake, physical activity, multivitamin use, family history of type 2 diabetes, anti-hypertensive drug use, cholesterol-lowering drug use, history of hypertension at baseline, socioeconomic status, and body mass index, total red meat, poultry, fish, egg, total dairy, nuts and legumes, fruits, vegetables, SSB, whole grains, and refined grains, baked, boiled or mashed potatoes (for French fries), and French fries (for baked, boiled, or mashed potatoes).

[1] BMI: body mass index; FFQ: food frequency questionnaire; T2D: type 2 diabetes.

Supplementary table 24. List of confounders among studies included in the meta-analysis.

	Hodge et al., 2004	Liu et al., 2004	Montonenet al., 2005	von Ruestenet al., 2013	Chen et al., 2018	Farhadnejad et al., 2018	Yiannakou et al., 2022	Pokharel et al., 2023	Wan et al., 2023	Pokharel et al., 2024	Mousavi et al., current
**Primary confounders**											
Age	X	X	X	X	X	X	X	X	X	X	X
Sex	X	NA	X	X	X	X	X	X	X	X	NA
Body mass index	X	X	X	X	X	X	X	X	X	X	X
Smoking status	X	X	X	X	X	X			X	X	X
Physical activity	X	X		X	X	X		X	X	X	X
Alcohol intake	X			X	X			X	X	X	X
Total energy intake	X		X	X	X	X			X		X
**Secondary confounders**											
Red meat and/or processed meat intake				X	X		X	X	X	X	X
Refined grains				X				X		X	X
Whole grains				X	X	X		X	X	X	X
Milk/Dairy product intake				X				X		X	X
Fruits intake				X		X	X		X	X	X
Vegetables intake				X		X	X		X		X
Poultry intake				X	X			X	X	X	X
Fish intake				X	X			X	X	X	X
Egg intake				X						X	X
Sugar-sweetened beverages intake				X	X			X	X		X
Nuts and legumes intake				X	X	X				X	X
**Other confounders**											
Ethnicity	X								X		X
Family history of diabetes	X	X				X			X		X
History of hypertension		X		X	X	X			X	X	X

Anti-hypertensive drug use											X
Cholesterol-lowering drug use									X	X	X
Menopause/menopausal status											X
Hormone therapy								X			X
Socioeconomic status									X	X	X
Multivitamin use				X					X		X
Aspirin use									X		
Education level	X			X	X		X	X	X	X	
Weight change in the last 5 years	X										
Waist-to-hip ratio	X			X			X				
History of high cholesterol		X		X		X			X		
Residential location/geographic area			X								
Hip circumference							X				
Waist circumference											
Hormone therapy											
Income										X	
Intakes of:											
Coffee				X	X			X			
Saturated fat						X					
Vegetable oil								X			
Animal fats								X			
Pasta, rice				X							
Soy					X						
Desserts				X							
Sweet bun/biscuit				X							
Fat (butter, oil, etc.)				X							
Chips				X							
Pizza				X							
Cakes, cookies				X							
Water				X							
Tea				X							
Sauce				X							
Fruit juice				X							
Low-energy soft drinks				X							

Supplementary Table 25. Assessment of risk of bias with the Newcastle-Ottawa Scale.[1]

	Selection				Comparability		Outcome			
**Author, year**	**Representativeness of the exposed cohort**	**Selection of the non-exposed cohort**	**Ascertainment of exposure**	**Outcome of interest was not present at baseline**	**Control for primary confounders**	**Control for secondary confounders**	**Assessment of outcome**	**Duration of follow-up**	**Adequacy of follow-up**	**Total**
Hodge et al., 2004	1	1	0	1	1	0	1	0	1	6
Liu et al., 2004	0	1	0	1	0	0	0	0	0	2
Montonenet al., 2005	1	1	0	1	0	0	1	1	0	5
von Ruestenet al., 2013	1	1	0	1	1	1	1	0	1	7
Chen et al., 2018	1	1	0	1	1	1	1	1	1	8
Farhadnejad et al., 2018	1	1	0	1	0	0	1	0	0	4
Yiannakou et al., 2022	0	1	1	1	0	1	1	1	1	7
Pokharel et al., 2023	1	1	0	1	0	1	1	1	1	7
Wan et al., 2023	1	1	1	1	1	1	1	1	1	9
Pokharel et al., 2024	1	1	0	1	0	1	1	1	0	6
Mousavi et al., current	0	1	1	1	1	1	1	1	1	8

[1] Representativeness of the exposed cohort: 1 point awarded if community-based population.
Selection of the non-exposed cohort: 1 point allocated if drawn form the same community as the exposed cohort.
Ascertainment of exposure: 1 point awarded if diet assessed at baseline and reassessed at least one time during follow-up.
Outcome of interest does not present at start of the study: 1 point awarded if individuals with prevalent diabetes at baseline were excluded.
Control for primary confounders: 1 point awarded if adjustment for age, sex, body mass index (BMI), smoking status, physical activity, alcohol intake, and total energy intake.
Control for secondary confounders: 1 point awarded if adjustment for meat intake, and intakes of ≥1 additional food associated with potato consumption; Assessment of outcome: 1 point awarded if self-reported cases were confirmed using additional measures (e.g. fasting blood glucose, supplemental questionnaires, physician's diagnosis, etc.).
Duration of follow-up; 1 point awarded if follow-up ≥ 10 years.
Adequacy of follow-up: 1 point awarded if loss to follow-up <20%.

Supplementary table 26. ROBINS-E judgement for each domain and overall.

Study	Bias due to confounding[1]	Bias arising from measurement of exposure [2]	Bias due to selection of participants into the study	Bias due to post-exposure interventions	Bias due to missing data	Bias arising from measurement of the outcome	Bias due to selective reporting of the results	Overall judgement
Hodge et al., 2004	Some concerns	Some concerns	Some concerns	Some concerns	Some concerns	Low	Low	Some risk of bias
Liu et al., 2004	High	Some concerns	Some concerns	Some concerns	Some concerns	Low	Low	High
Montonenet al., 2005	High	Some concerns	Some concerns	Some concerns	Some concerns	Low	Low	High
Von Ruestenet al., 2013	Some concerns	Some concerns	Some concerns	Some concerns	Low	Low	Low	Some risk of bias
Chen et al., 2018	Some concerns	Some concerns	Low	Some concerns	Low	Low	Low	Some risk of bias
Farhadnejad et al., 2018	Some concerns	Some concerns	Some concerns	Some concerns	Some concerns	Low	Low	Some risk of bias
Yiannakou et al., 2022	High	Some concerns	Some concerns	Low	Low	Low	Low	High
Pokharel et al., 2023	High	Some concerns	Some concerns	Some concerns	Low	Low	Low	High
Wan et al., 2023	Some concerns	Some concerns	Low	Low	Low	Low	Low	Some risk of bias
Pokharel et al., 2024	High	Some concerns	Some concerns	Some concerns	Some concerns	Low	Low	High
Mousavi et al., (current)	Some concerns	Some concerns	Low	Low	Low	Low	Low	Some risk of bias

[1] Due to the inherent residual confounding in observational studies and the possibility of measurement errors in dietary assessments, the risk of bias in both confounding and exposure assessments inevitably exists, making it unattainable to achieve a low risk of bias in these domains.

[2] All dietary assessment methods are subject to measurement error; therefore, no study was given a low risk of bias.

Supplementary table 27. Pre-specified subgroup meta-analyses of the association between every 3-serving/week total potato intake and risk of type 2 diabetes.[1]					
**Stratification**	**Categories**	**Risk estimates, n**	**Pooled Hazard Ratio (95% CI)**	**I^2 (%)**	***P* interaction[2]**
All studies		11	1.03 (1.02 to 1.05)	57.3	
Mean age of participants	<55 years	6	1.043(1.00 to 1.07)	58.7	0.91
	≥55 years	5	1.03 (1.02 to 1.05)	64.1	
Geographic location	USA	5	1.04 (1.01 to 1.07)	0.0	**0.009**
	Europe	2	1.04 (1.02 to 1.07)	24.2	
	Asia	4	0.96 (0.93 to 1.00)	54.1	
Follow-up duration	<15 years	6	1.02 (1.00 to 1.04)	73.5	0.09
	≥15 years	5	1.05 (1.02 to 1.08)	0.0	
Number of participants	<40,000	6	1.02 (0.97 to 1.07)	59.0	0.54
	≥40,000	5	1.03 (1.02 to 1.05)	63.1	
Number of cases	<2,000	6	1.02 (0.97 to 1.07)	59.1	0.54
	>2,000	5	1.03 (1.02 to 1.05)	63.1	
Gender	Men	1	1.06 (1.00 to 1.13)	-	0.24
	Women	3	1.04 (1.01 to 1.07)	0.0	
	Both	7	1.03 (1.01 to 1.05)	71.4	
Adjustment for all primary confounders	No	5	1.02 (0.97 to 1.07)	66.1	0.72
	Yes	6	1.03 (1.02 to 1.05)	56.5	
Adjustment for at least one secondary confounders	No	3	1.03 (0.98 to 1.09)	34.4	0.83
	Yes	8	1.03 (1.02 to 1.05)	65.5	
Risk of bias	<7	6	1.02 (0.97 to 1.07)	59.1	0.55
	≥7	5	1.03 (1.02 to 1.05)	63.1	
Dietary assessment method	Food recall and record	2	1.04 (1.02 to 1.06)	0.0	0.28
	FFQ	9	1.02 (1.00 to 1.05)	63.5	
Dietary assessment	Baseline only	6	0.99 (0.96 to 1.03)	65.2	**0.004**
	Repeated	5	1.04 (1.03 to 1.06)	0.0	

[1] Pooled hazard ratio are from a fixed-effects and I-squared refers to the proportion of heterogeneity between studies.
[2] Calculated using meta-regression.

**Supplementary table 28.** Pre-specified subgroup meta-analyses of the association between every 3-serving/week fried potato intake and risk of type 2 diabetes.[1]					
**Stratification**	**Categories**	**Risk estimates, n**	**Pooled Hazard Ratio (95% CI)**	**I^2 (%)**	***P* interaction[2]**
All studies		9	1.16 (1.09 to 1.23)	50.9	
Mean age of participants	<55 years	5	1.10 (1.00 to 1.19)	44.4	0.08
	≥55 years	4	1.22 (1.12 to 1.32)	49.9	
Geographic location	USA	4	1.18 (1.11 to 1.26)	45.2	**0.03**
	Europe	3	1.06 (0.91 to 1.23)	4.5	
	Asia	2	0.69 (0.41 to 1.17)	68.2	
Follow-up duration	<15 years	4	1.00 (0.86 to 1.16)	43.8	**0.03**
	≥15 years	5	1.18 (1.11 to 1.26)	38.2	
Number of participants	<40,000	4	0.97 (0.81 to 1.17)	43.2	0.06
	≥40,000	5	1.18 (1.11 to 1.25)	45.3	
Number of cases	<2,000	4	0.97 (0.81 to 1.17)	43.2	0.06
	>2,000	5	1.18 (1.11 to 1.25)	45.3	
Gender	Men	1	1.23 (1.09 to 1.39)	-	**0.02**
	Women	2	1.18 (1.09 to 1.28)	60.4	
	Both	6	1.01 (0.89 to 1.15)	33.9	
Adjustment for all primary confounders	No	4	0.98 (0.79 to 1.21)	58.3	0.11
	Yes	5	1.17 (1.10 to 1.24)	37.9	
Risk of bias	<7	3	0.92 (0.73 to 1.15)	55.4	**0.04**
	≥7	6	1.17 (1.10 to 1.25)	33.3	
Dietary assessment method	Food recall and record	2	1.00 (0.86 to 1.15)	0.0	**0.03**
	FFQ	7	1.19 (1.12 to 1.27)	48.0	
Dietary assessment	Baseline only	4	1.05 (0.81 to 1.35)	59.6	0.43
	Repeated	5	1.16 (1.09 to 1.23)	51.5	

[1] Pooled hazard ratio are from a fixed-effects and I-squared refers to the proportion of heterogeneity between studies.
[2] Calculated using meta-regression.

Supplementary table 29. Pre-specified subgroup meta-analyses of the association between every 3-serving/week non-fried potato intake and risk of type 2 diabetes.[1]

**Stratification**	**Categories**	**Risk estimates, n**	**Pooled Hazard Ratio (95% CI)**	**I^2 (%)**	***P* interaction[2]**
All studies		6	1.01 (0.99 to 1.03)	0.0	
Mean age of participants	<55 years	3	1.00 (0.95 to 1.06)	0.0	0.80
	≥55 years	3	1.01 (0.99 to 1.03)	0.0	
Geographic location	USA	4	1.01 (0.98 to1.05)	0.0	0.95
	Europe	2	1.01 (0.99 to 1.03)	0.0	
Follow-up duration	<15 years	1	1.02 (0.91 to 1.14)	0.0	0.86
	≥15 years	2	1.01 (0.99 to 1.03)	0.0	
Number of participants	<40,000	2	1.01 (0.92 to 1.11)	0.0	0.95
	≥40,000	4	1.01 (0.99 to 1.03)	0.0	
Number of cases	<2,000	2	1.01 (0.92 to 1.11)	0.0	0.95
	>2,000	4	1.01 (0.99 to 1.03)	0.0	
Gender	Men	1	1.02 (0.95 to 1.09)	-	0.95
	Women	2	1.01 (0.97 to 1.05)	0.0	
	Both	3	1.01 (0.99 to 1.03)	0.0	
Adjustment for all primary confounders	No	2	1.01 (0.99 to 1.03)	0.0	0.88
	Yes	4	1.01 (0.98 to 1.05)	0.0	
Risk of bias	<7	1	0.99 (0.81 to 1.21)	-	0.84
	≥7	5	1.01 (0.99 to 1.03)	0.0	
Dietary assessment method	Food recall and record	1	0.99 (0.81 to 1.21)	0.0	0.86
	FFQ	5	1.01 (0.99 to 1.03)	0.0	
Dietary assessment	Baseline only	2	1.01 (0.99 to 1.03)	0.0	0.84
	Repeated	4	1.01 (0.98 to 1.05)	0.0	

1 Pooled hazard ratio are from a fixed-effects and I-squared refers to the proportion of heterogeneity between studies.
2 Calculated using meta-regression.

**Supplementary Table 30.** Summary hazard ratios from nonlinear dose-response analysis of potato consumption and risk of T2D				
	Total potato	Fried potato	Non-fried potato	Boiled potato
Serving/week	HR (95% CI)	HR (95% CI)	HR (95% CI)	HR (95% CI)
0	1.00	1.00	1.00	1.00
1	1.00 (0.98 to 1.01)	1.07 (1.04 to 1.10)	0.99 (0.97 to 1.02)	1.01 (0.99 to 1.04)
2	1.00 (0.96 to 1.03)	1.11 (1.06 to 1.15)	0.99 (0.95 to 1.04)	1.03 (0.98 to 1.07)
3	1.00 (0.96 to 1.04)	1.12 (1.07 to 1.17)	0.99 (0.94 to 1.05)	1.04 (0.98 to 1.09)
4	1.01 (0.96 to 1.06)	1.12 (1.07 to 1.17)	1.00 (0.94 to 1.05)	1.04 (0.98 to 1.10)
5	1.03 (0.97 to 1.08)	1.11 (1.06 to 1.16)	1.00 (0.95 to 1.06)	1.04 (0.99 to 1.10)
6	1.04 (0.99 to 1.10)	1.10 (1.05 to 1.15)	1.01 (0.95 to 1.07)	1.04 (0.99 to 1.10)
7	1.06 (1.01 to 1.12)	1.09 (1.03 to 1.15)	1.01 (0.96 to 1.08)	1.05 (0.99 to 1.10)
8	1.09 (1.03 to 1.14)	1.08 (1.02 to 1.15)	1.02 (0.96 to 1.09)	1.05 (1.00 to 1.10)
9	1.11 (1.06 to 1.17)	1.08 (1.00 to 1.15)	1.03 (0.96 to 1.10)	1.05 (1.00 to 1.11)
10	1.13 (1.07 to 1.19)	1.07 (0.99 to 1.16)	1.03 (0.97 to 1.11)	1.05 (1.00 to 1.11)
11	1.16 (1.09 to 1.22)	1.06 (0.97 to 1.16)	1.04 (0.97 to 1.12)	1.05 (0.99 to 1.12)
12	1.18 (1.11 to 1.25)	1.05 (0.96 to 1.16)	1.05 (0.97 to 1.13)	1.06 (0.99 to 1.13)
13	1.21 (1.13 to 1.29)	1.05 (0.94 to 1.17)	-	1.06 (0.99 to 1.13)
14	1.23 (1.15 to 1.32)	1.04 (0.93 to 1.17)	-	1.06 (0.99 to 1.14)
15	1.26 (1.17 to 1.36)	1.03 (0.91 to 1.17)	-	1.06 (0.98 to 1.15)
16	1.28 (1.18 to 1.39)	1.03 (0.90 to 1.18)	-	1.07 (0.98 to 1.16)
17	1.31 (1.20 to 1.43)	1.02 (0.88 to 1.18)	-	1.07 (0.98 to 1.17)
18	1.34 (1.22 to 1.47)	1.01 (0.87 to 1.19)	-	1.07 (0.97 to 1.18)
P-nonlinearity	0.03	<0.001	0.16	0.41
P dose-response	<0.001	0.02	0.18	0.11

Supplementary Table 31. GRADE evidence table for the association of potatoes intake with risk of T2D.

Certainty assessment							No of participants / cases		Effect	Certainty	Importance
No of estimates	Study design	Risk of bias	Inconsistency	Indirectness	Imprecision	Other considerations	Participants	Cases	Summary hazard ratio (95% confidence interval)		
**Total potato**											
11	observational studies	serious [a]	not serious [b]	not serious	Serious [c]	dose-response gradient [d]	508,790	34,945	1.03 (1.02 to 1.05)	⊕⊕⊕◯ MODERATE	Critical
**Fried potato**											
9	observational studies	serious [e]	not serious [f]	not serious	not serious	dose-response gradient [g]	467,740	35,908	1.16 (1.09 to 1.23)	⊕⊕⊕⊕ HIGH	Critical
**Non-fried potato**											
6	observational studies	serious [h]	not serious	not serious	serious [i]	none	285,987	31,375	1.01 (0.99 to 1.03)	⊕⊕◯◯ LOW	Critical
**Boiled potato**											
3	observational studies	serious [j]	not serious [k]	serious [l]	serious [m]	none	231,439	11,886	1.01 (0.99 to 1.03)	⊕◯◯◯ VERY LOW	Critical
**Mashed potato**											
2	observational studies	serious [n]	not serious	serious [o]	Serious [p]	dose-response gradient [q]	229,458	11,754	1.06 (1.02 to 1.09)	⊕⊕◯◯ LOW	Critical

Explanations

a. Downgraded since three studies with low weighting (30%) judged as serious risk of bias based on ROBINS-I was included in the meta-analysis and residual confounding cannot be ruled out.
b. Serious inconsistency since I^2 = 54.5%, P=0.02; however, subgroup analysis by geographic location partly explained the heterogeneity, while maintaining the same direction and significance of the association. Not downgraded.
c. Serious imprecision as the upper bound of the 95% CI (HR, 1.05) includes the minimally important difference (MID) of 5% while the lower bound (HR, 1.02) crosses to the MID. Downgraded
d. Upgrade for a dose response gradient as a non-linear dose response was observed ($P_{\text{non-linearity}}$ =0.002)
e. Downgraded since two studies with low weighting (25%) judged as serious risk of bias based on ROBINS-I was included in the meta-analysis and residual confounding cannot be ruled out.
f. Serious inconsistency since I^2 = 50.6%, P = 0.05; however, sensitivity analysis removing Farhadnejad et al., 2018 explained most of the heterogeneity (I^2 = 33.3%, P = 0.18), while maintaining the same direction and significance of the association. Not downgraded.
g. Upgrade for a dose response gradient as a non-linear dose response was observed ($P_{\text{non-linearity}}$ <0.001)
h. Downgraded since two studies with low weighting (33%) judged as serious risk of bias based on ROBINS-I was included in the meta-analysis and residual confounding cannot be ruled out.
i. Downgrade for serious imprecision as both the lower (HR, 0.99) and upper (HR, 1.03) bound of the 95% CI cross the minimally important difference (MID) of the 5%
j. Residual confounding cannot be ruled out. Downgraded
k. Serious inconsistency since I^2 = 62.5%, P=0.02; however, sensitivity analysis removing Farhadnejad et al., 2018 explained all of the heterogeneity (I^2 = 0.0%, P = 0.99), while maintaining the same direction and significance of the association. Not downgraded.

l. Downgrade for serious indirectness as evidence is based on a predominately (>99%) European population and may not be generalizable to different populations.
m. Downgrade for serious imprecision as both the lower bound (HR, 0.99) and upper bound (HR, 1.03) of the 95% CI cross the minimally important difference (MID) of the 5%
n. Residual confounding cannot be ruled out. Downgraded
o. Downgrade for serious indirectness as all the evidence is based in Europe and may not be generalizable to different populations.
p. Downgrade for serious imprecision as the upper bound of the 95% CI (HR, 1.09) includes the minimally important difference (MID) of 5% while the lower bound (HR, 1.02) cross the MID.
q. Upgrade for a dose response gradient as a linear dose response was observed ($P_{linearity}$ =0.002)

**Supplementary Table 32.** NutriGrade Scoring system of summary evidence for the association of potato intake with incident of T2D											
**Exposure**	**Outcome**	**Risk of bias[1] (max of 2 points)**	**Precision[2] (max of 1 points)**	**Heterogeneity[3] (max of 1 points)**	**Directness[4] (max of 1 points)**	**Publication bias[5] (max of 1 points)**	**Funding bias[6] (max of 1 points)**	**Effect size[7] (max of 2 points)**	**Dose-response[8] (max of 1 points)**	**Overall**	**Certainty***
Total potato	T2D	1	1	0.6	1	1	1	0	1	6.6	Moderate
Fried potato	T2D	2	1	0.3	1	1	1	0	1	7.3	Moderate
Non-fried potato	T2D	2	1	0.4	1	1	1	0	0	6.4	Moderate
Boiled potato	T2D	1	1	0	1	1	1	0	0	5	Low
Mashed potato	T2D	2	1	0	1	1	1	0	1	7	Moderate

[1] Newcastle Ottawa Scale (mean): ≥7 (2 points); 4-6.9 (1 point), 0-3.9 (0 point).
[2] >5000 cases and the 95%CI excludes the null value (1 point), ≥500 cases, but 95% CI overlaps the null value (i.e., CI includes RR of 1.0), and 95% CI excludes important benefit (RR of <0.8) or harm (RR of <1.2) (1 point).
[3] 0 to 1 point based on I^2.
[4] No important differences in the population or hard clinical outcomes (1 point), important differences in the population (0 point).
[5] No evidence for publication bias with test or plot (≥10 studies) (1 point) or to align with GRADE criteria, awarding 1 point in cases where publication bias could not be determined (< 10 studies)
[6] Funded by academic institutions or research institutions (1 point), funded by private institutions, foundations, or nongovernmental organizations (0.5 point), Industry funding or conflict of interest (0 point).
[7] No effect (RR: 0.80–1.20) when comparing the highest vs. lowest category (0 point), moderate effect size (RR: <0.80–0.50 and >1.20–2, and corresponding test is statistically significant) when comparing the highest vs. lowest category (1 point), large effect size (RR: <0.50 and >2.00, and corresponding test is statistically significant) when comparing the highest vs. lowest category (2 point).
[8] No dose-response analysis or dose-response analysis with corresponding statistical test no significant (0 point), significant linear or nonlinear dose-response relationship (1 point).
* 0-3.99: very low meta-evidence; 4-5.99: low meta-evidence; 6-7.99: moderate meta-evidence; ≥8: high meta-evidence

Supplementary Figure 1. Flowchart of study participants

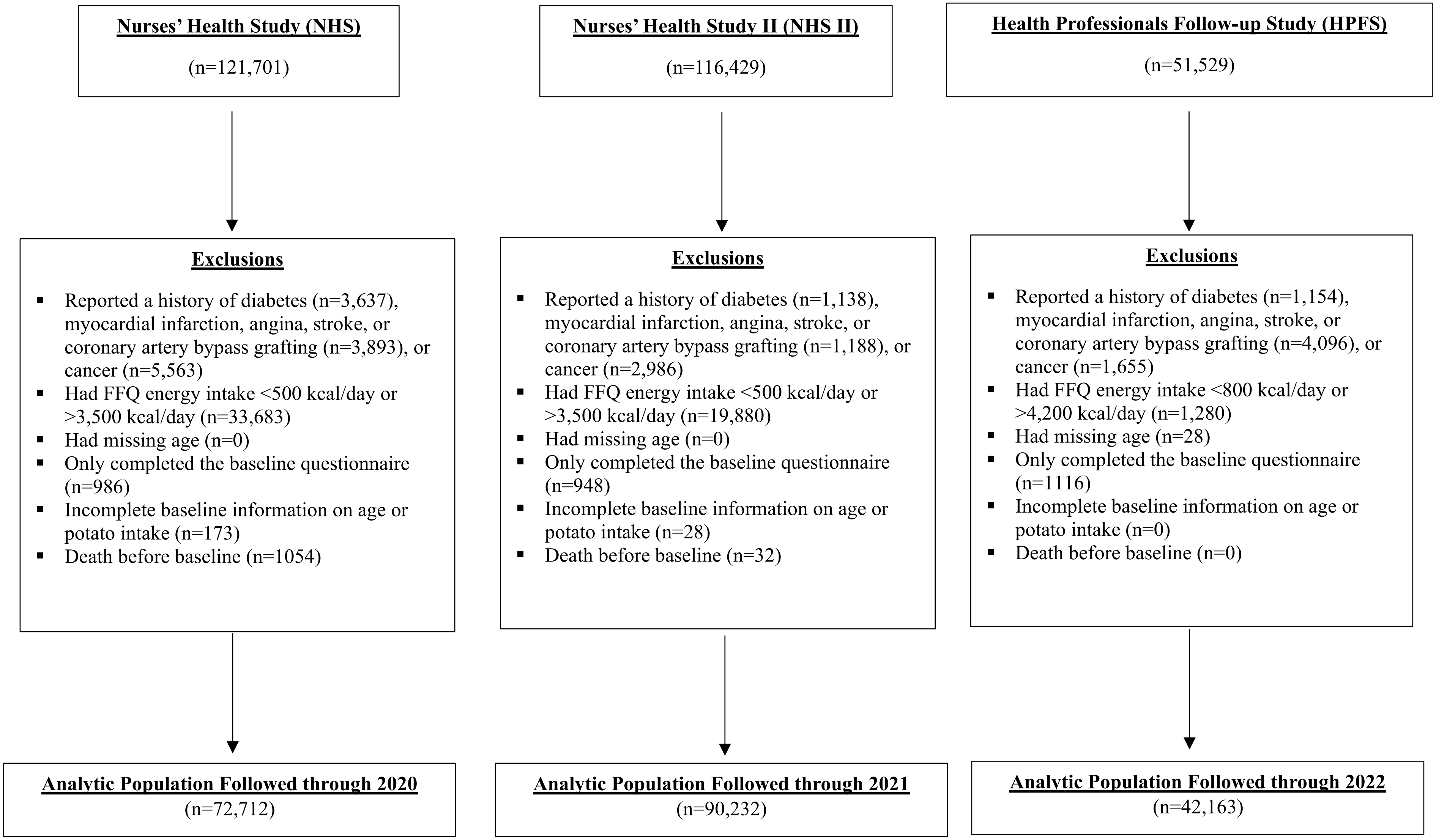

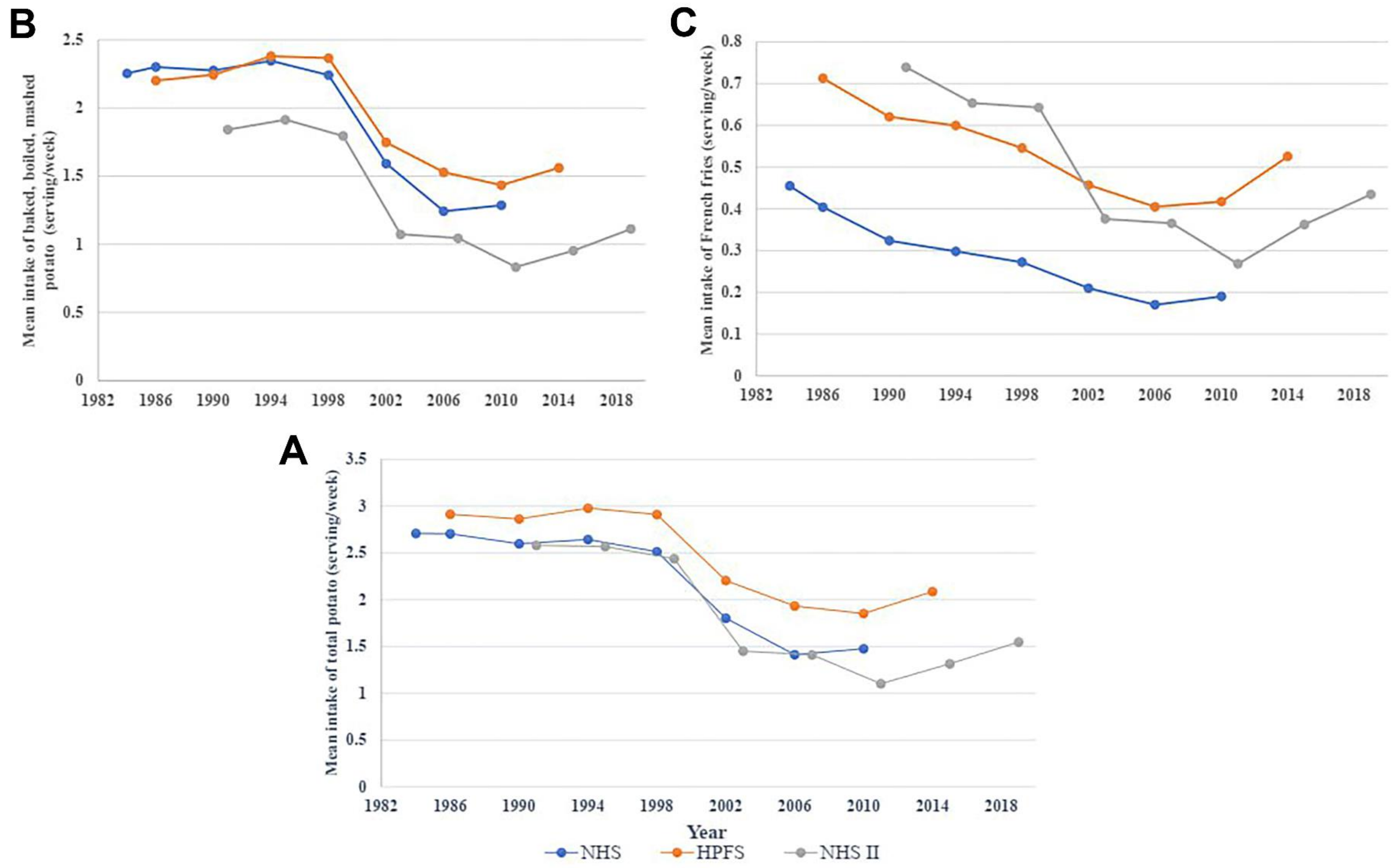

Supplementary Figure 2. Trends in mean A) total potato, B) baked, boiled, mashed potato, C) French fries consumption in the NHS, NHS II, and HPFS over time. (Mean potatoes intake calculated for each questionnaire returned.)

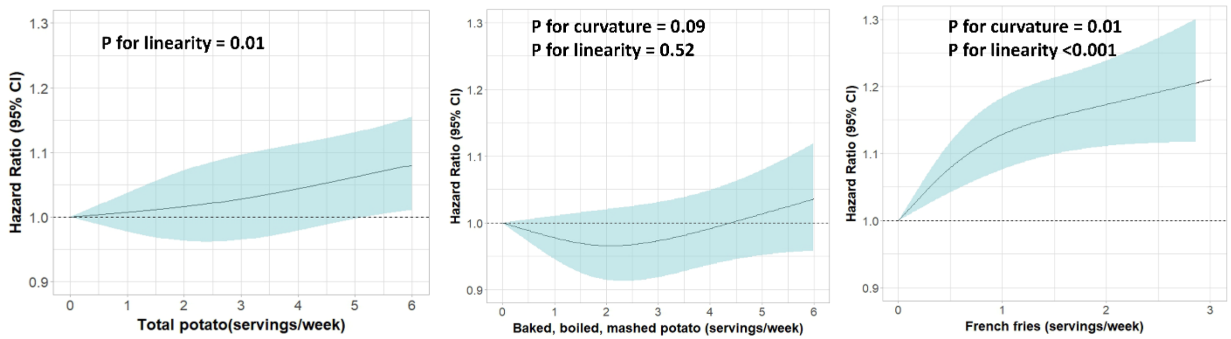

Supplementary Figure 3. Dose–response relationships between total potato intake, baked/boiled/mashed potatoes, and French fries and the incidence of type 2 diabetes. Data was pooled from three prospective cohorts (NHS, NHS II, and HPFS; total n = 205,107). Associations were estimated using restricted cubic spline models with three knots placed at the 10th, 50th, and 90th percentiles of intake, including interaction terms between cohort and covariates.

The model was stratified by age (months), cohort and calendar time (two-year interval) and adjusted for total energy intake, race/ethnicity (white adults, non-white adults), smoking status (never, past, current: 1-14 cigs/day, current: >15-24 cigs/day, current: >24 cigs/day), alcohol intake (non-alcohol drinker, 0-4.9 grams/day, 5-9.9 grams/day, 10-14.9 grams/day, 15-29.9 grams/day, >30 grams/day), physical activity (<3, 3-9, 9-18, 18-27, ≥ 27 METs-hr/week), multivitamin use, menopausal status and hormone use (in NHS or NHS II), family history of type 2 diabetes, anti-hypertensive drug use, cholesterol-lowering drug use, history of hypertension, socioeconomic status, time-varying body mass index (<21, 21-23, 23-25, 25-27, 27-30, 30-33, 33-35, 35-40, ≥40 kg/m2) and dietary covariates intakes (including total red meat, poultry, fish, egg, total dairy, nuts and legumes, fruits, vegetables, sugar-sweetened beverages, whole grain, and refined grain, and mutual adjustment for baked, boiled or mashed potatoes and French fries.

Abbreviations: NHS=Nurses' Health Study; NHSII=Nurses' Health Study II; HPFS=Health Professionals Follow-up Study; MET-h=metabolic equivalent tasks per hour.

A

B

C

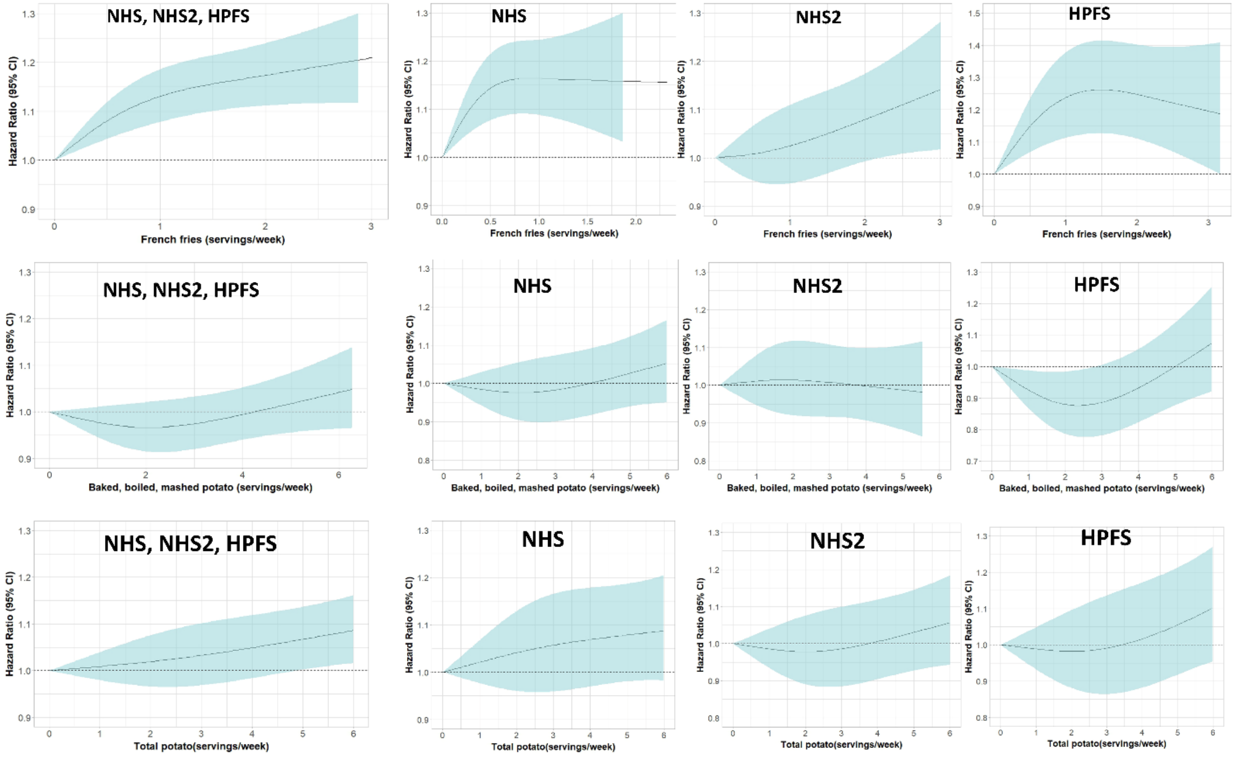

Supplementary Figure 4. Cohort-specific dose–response relationships between (A) total potato intake, (B) baked/boiled/mashed potatoes, and (C) French fries and the incidence of type 2 diabetes in the NHS, NHS II, and HPFS. Associations were estimated using restricted cubic spline models with three knots placed at the 10th, 50th, and 90th percentiles of intake.

The model was stratified by age (months), and calendar time (two-year interval) and adjusted for total energy intake, race/ethnicity (white adults, non-white adults), smoking status (never, past, current: 1-14 cigs/day, current: >15-24 cigs/day, current: >24 cigs/day), alcohol intake (non-alcohol drinker, 0-4.9 grams/day, 5-9.9 grams/day, 10-14.9 grams/day, 15-29.9 grams/day, >30 grams/day), physical activity (<3, 3-9, 9-18, 18-27, ≥ 27 METs-hr/week), multivitamin use, menopausal status and hormone use (in NHS or NHS II), family history of type 2 diabetes, anti-hypertensive drug use, cholesterol-lowering drug use, history of hypertension, socioeconomic status, time-varying body mass index (<21, 21-23, 23-25, 25-27, 27-30, 30-33, 33-35, 35-40, ≥40 kg/m2) and dietary covariates intakes (including total red meat, poultry, fish, egg, total dairy, nuts and legumes, fruits, vegetables, sugar-sweetened beverages, whole grain, and refined grain, and mutual adjustment for baked, boiled or mashed potatoes and French fries.

Abbreviations: NHS=Nurses' Health Study; NHSII=Nurses' Health Study II; HPFS=Health Professionals Follow-up Study; MET-h=metabolic equivalent tasks per hour.

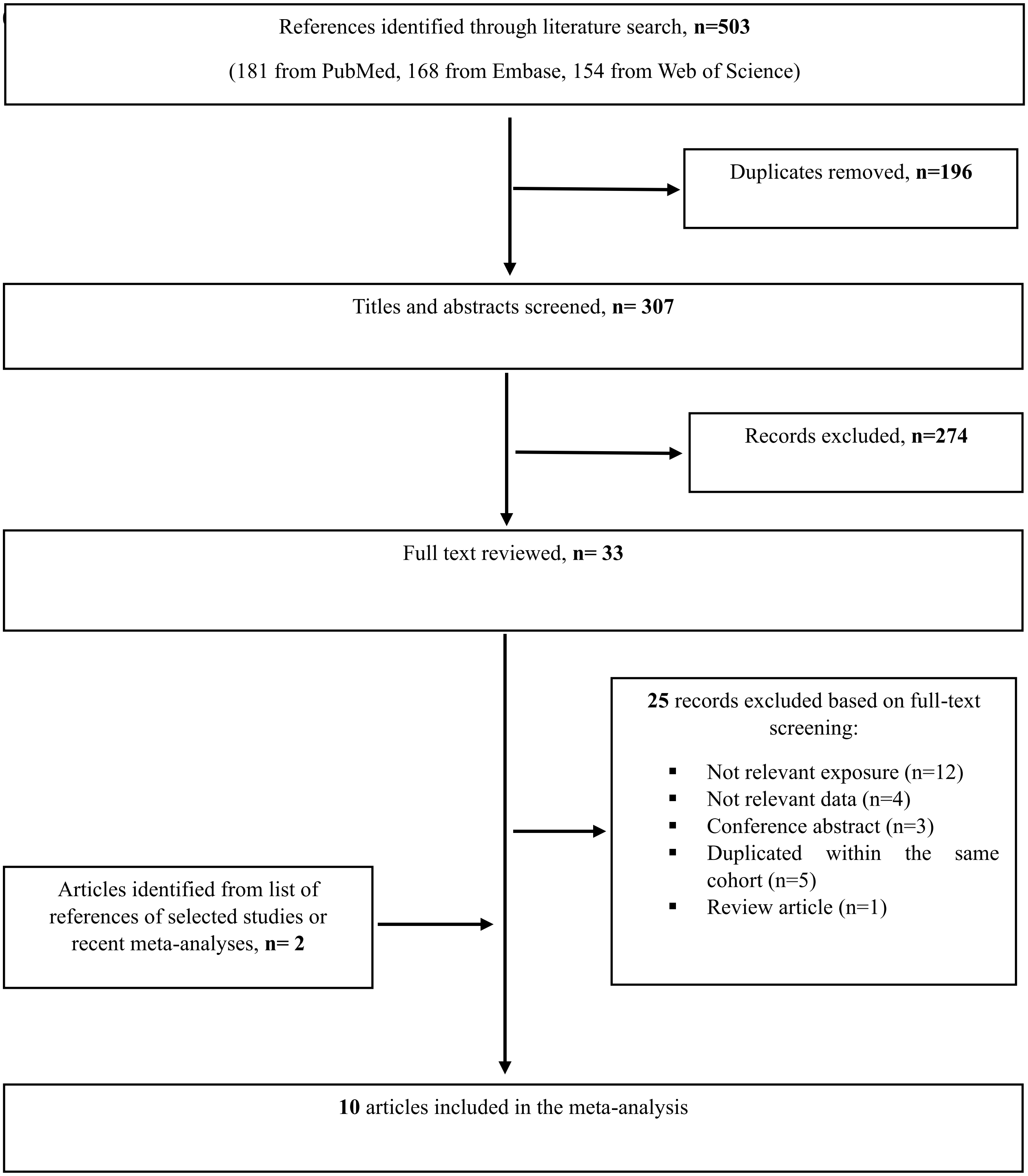

Supplementary Figure 5. Meta-analysis search strategy and study selection

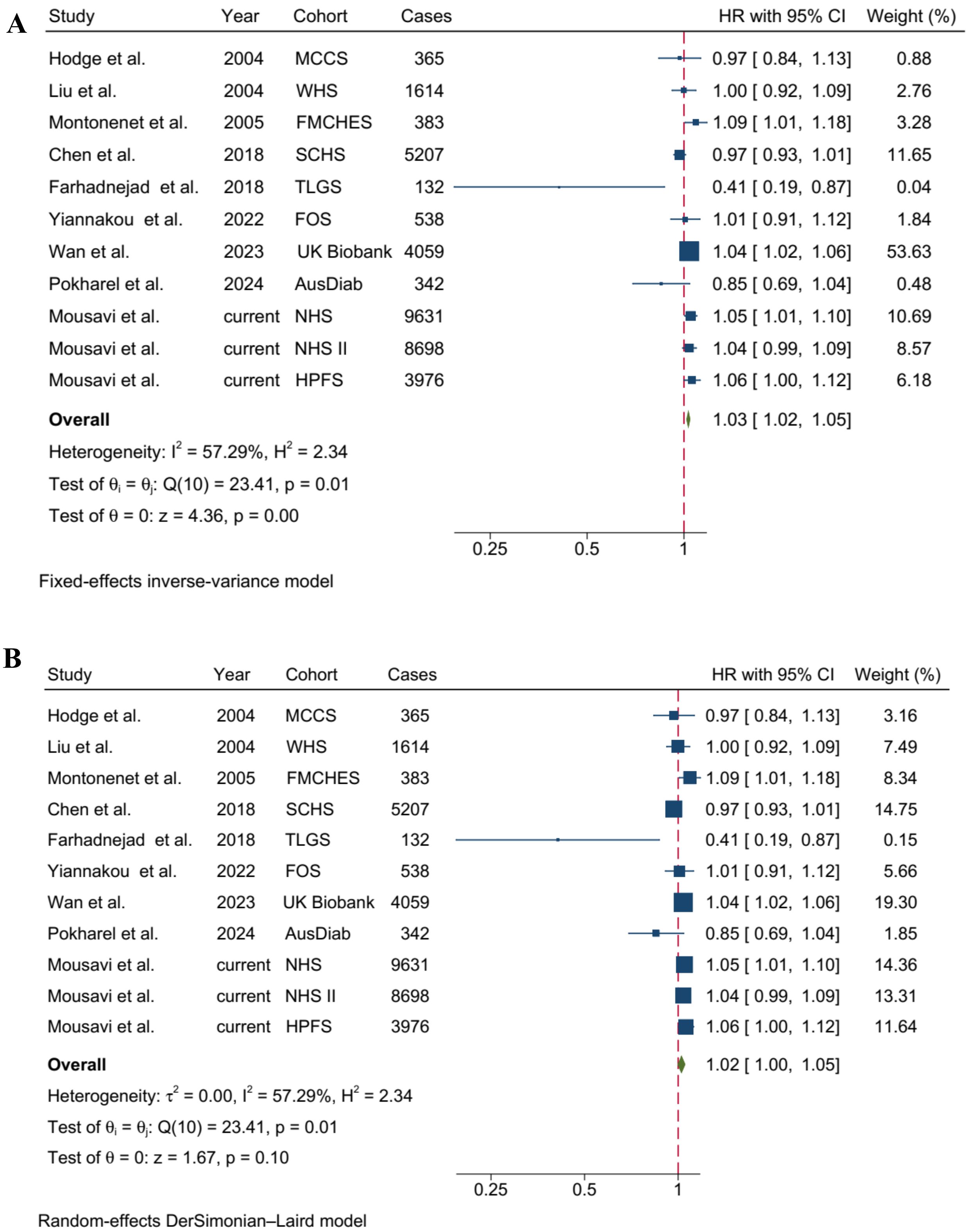

Supplementary Figure 6. Forest plot indicating summary hazard ratio of the association between 3-serving/week total potato intake and T2D: A) fixed-effects model, B: random-effects model.

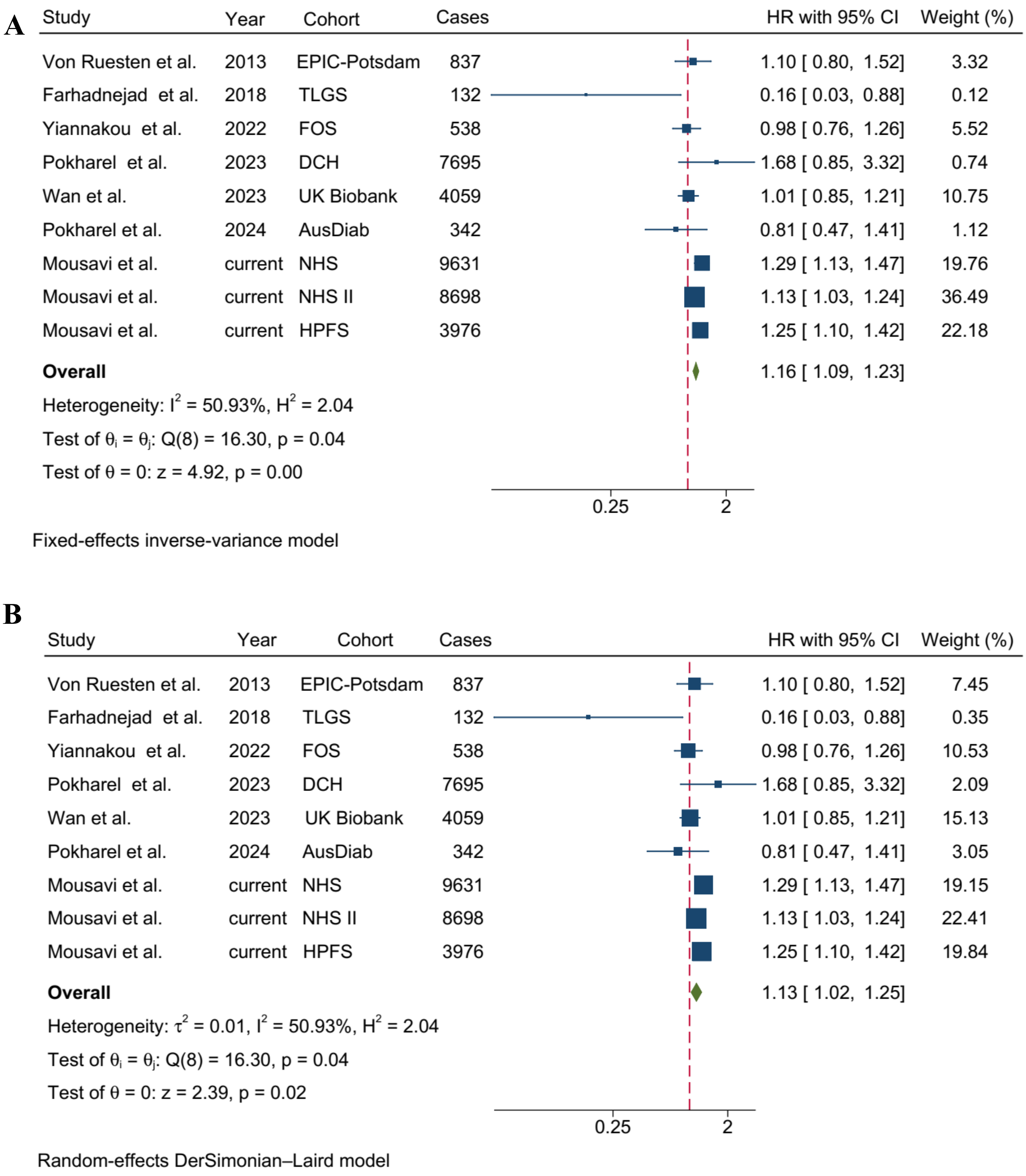

Supplementary Figure 7. Forest plot indicating summary hazard ratio of the association between 3-serving/week **fried potato** intake and T2D: A) fixed-effects model, B: random-effects model.

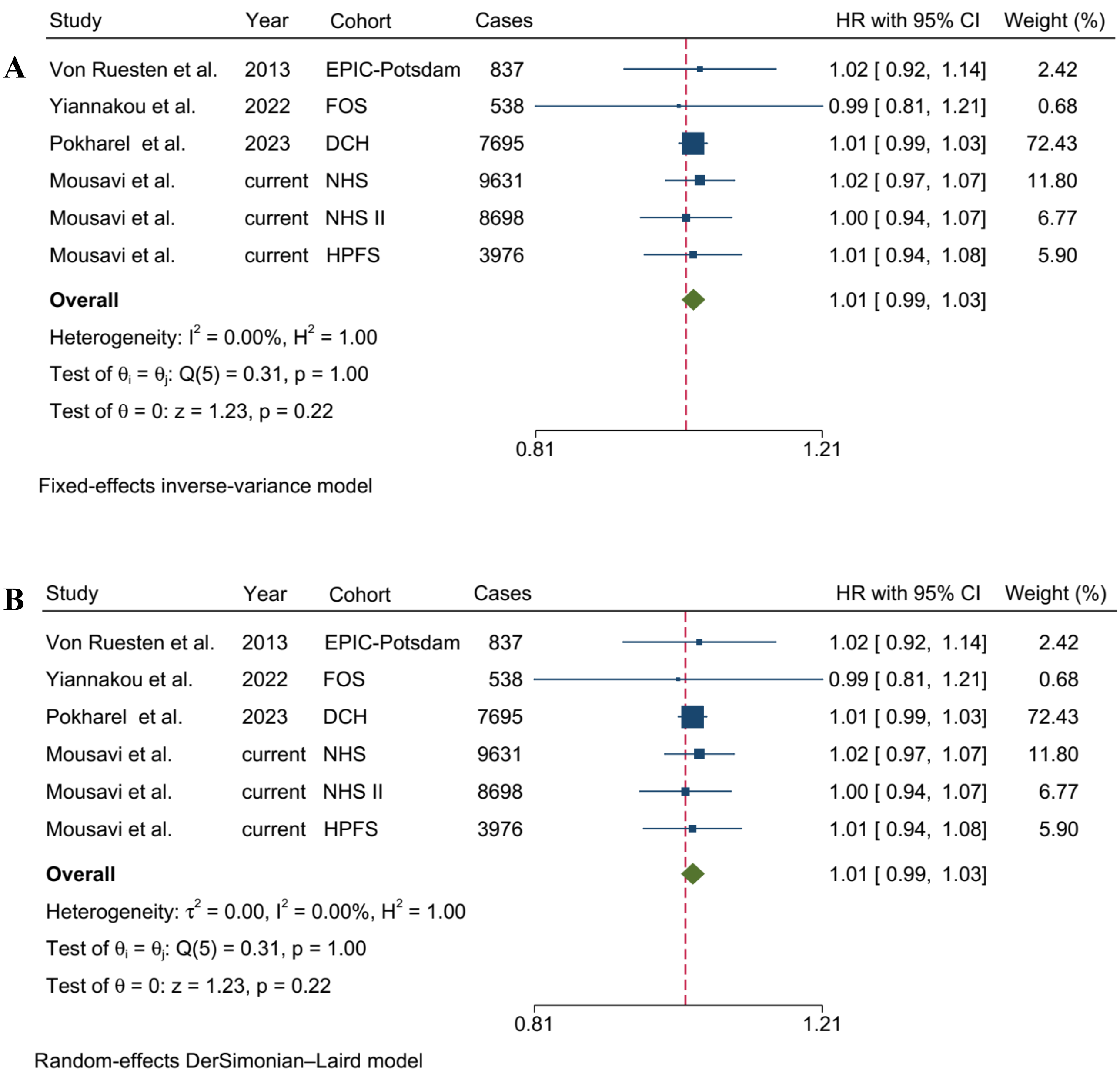

Supplementary Figure 8. Forest plot indicating summary hazard ratio of the association between 3-serving/week **non-fried potato** intake and T2D: A) fixed-effects model, B: random-effects model.

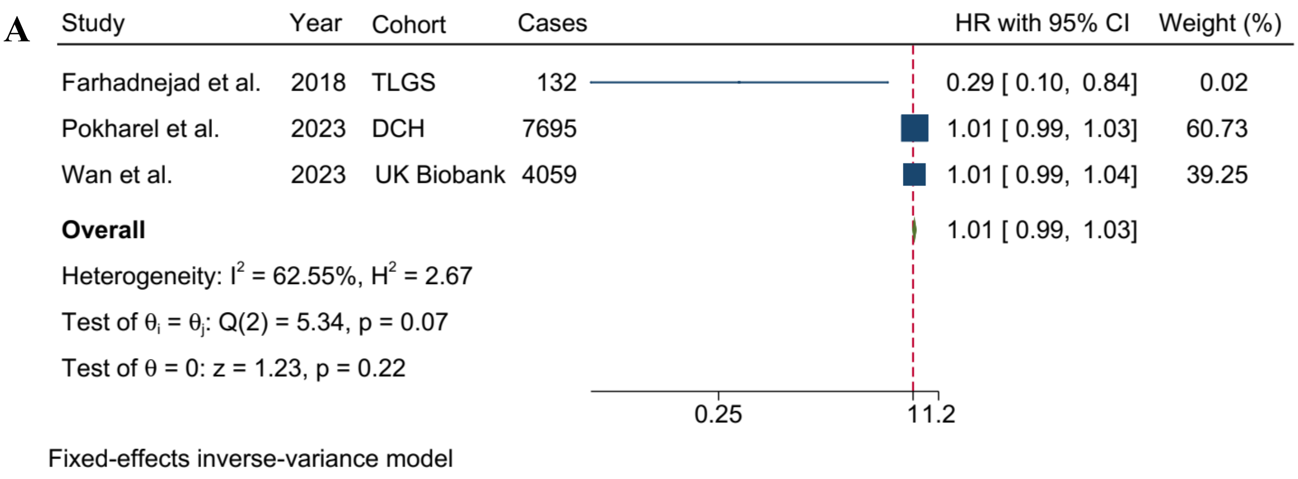

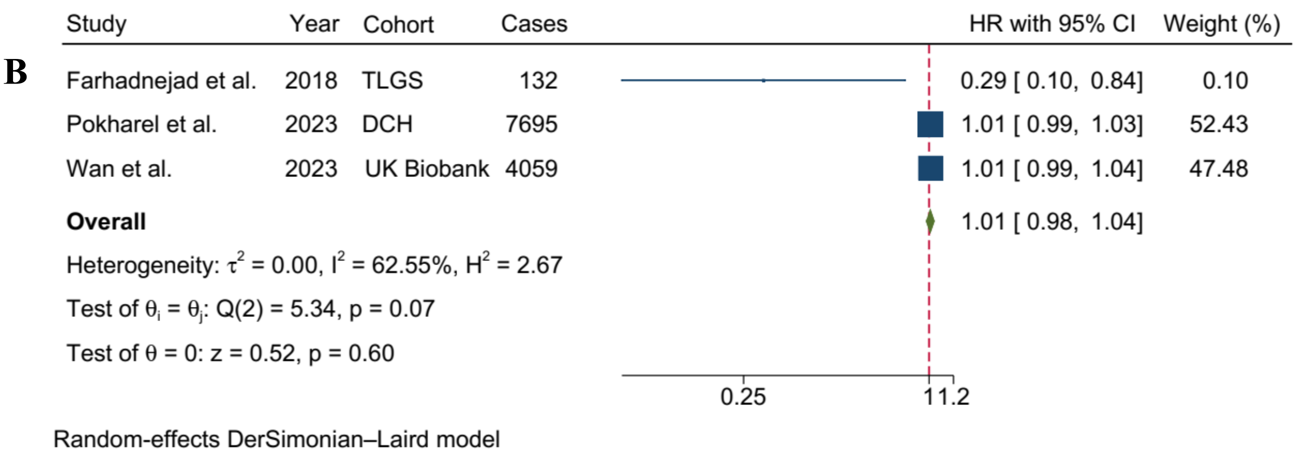

Supplementary Figure 9. Forest plot indicating summary hazard ratio of the association between 3-serving/week **boiled potato** intake and T2D: A) fixed-effects model, B: random-effects model.

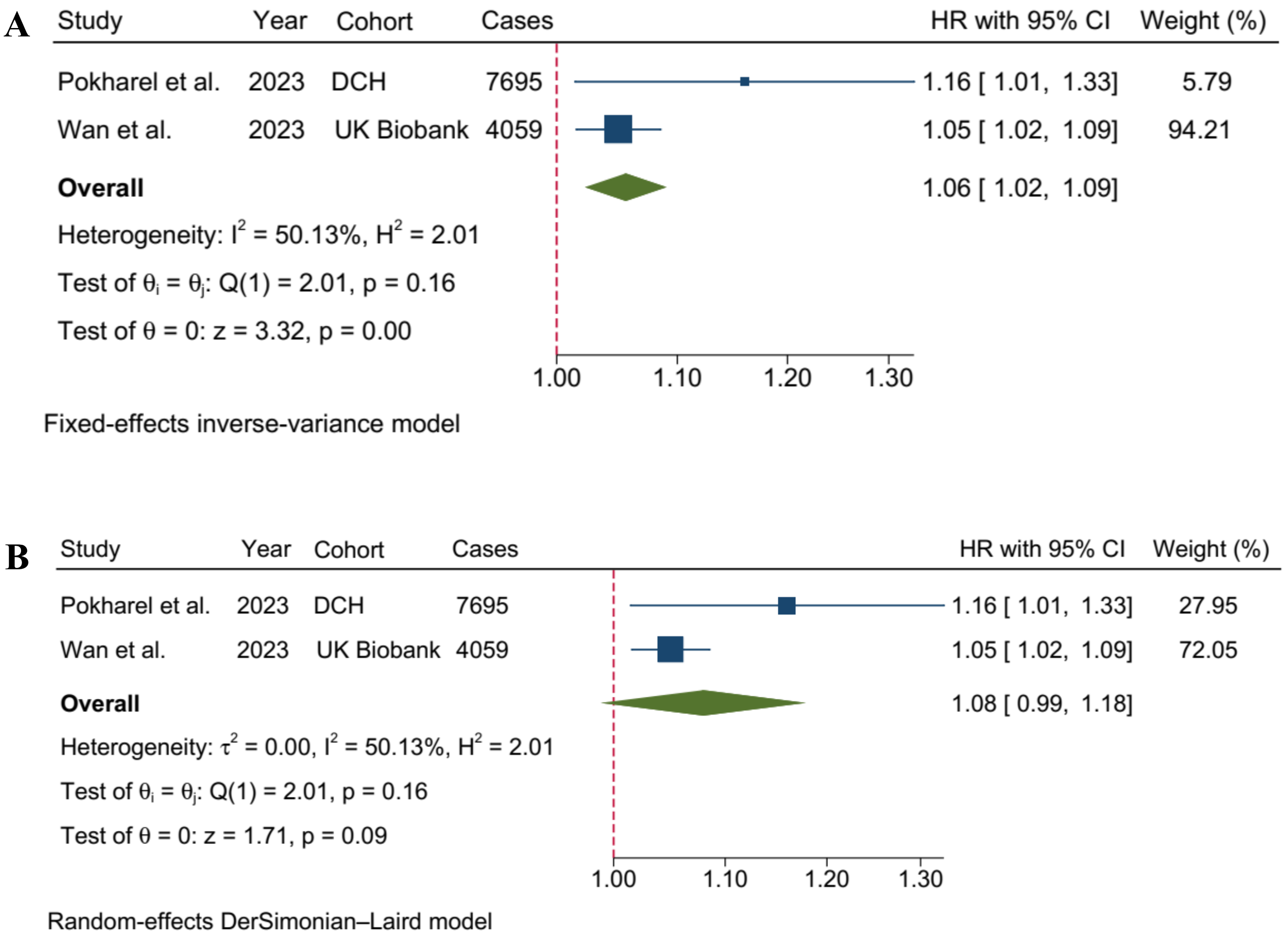

Supplementary Figure 10. Forest plot indicating summary hazard ratio of the association between 3-serving/week **mashed potato** intake and T2D: A) fixed-effects model, B: random-effects model

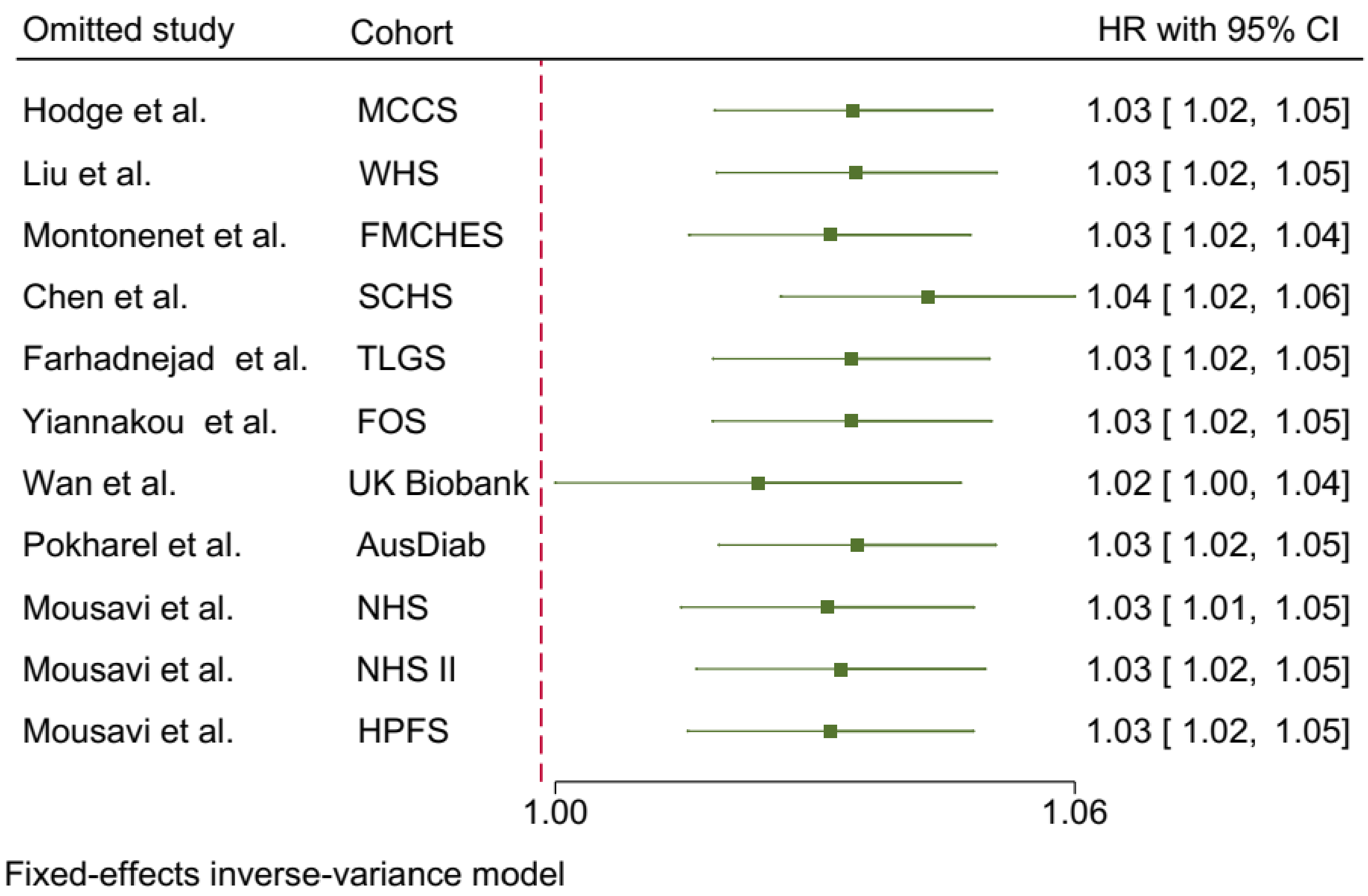

Supplementary Figure 11. Leave-one-out sensitivity analysis of the association between 3-serving/week **total potato** consumption and risk of T2D

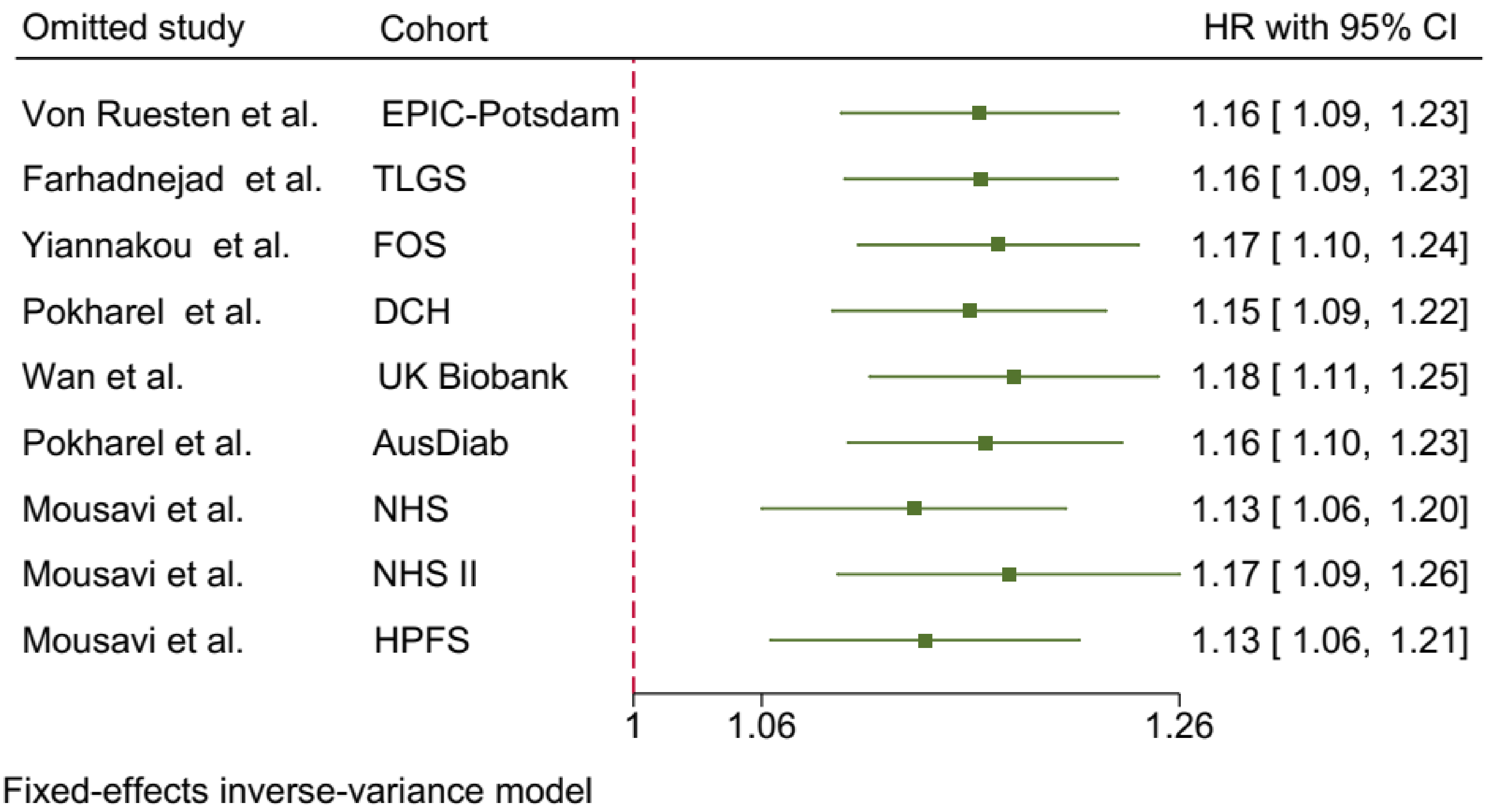

Supplementary Figure 12. Leave-one-out sensitivity analysis of the association between 3-serving/week **fried potato** consumption and risk of T2D

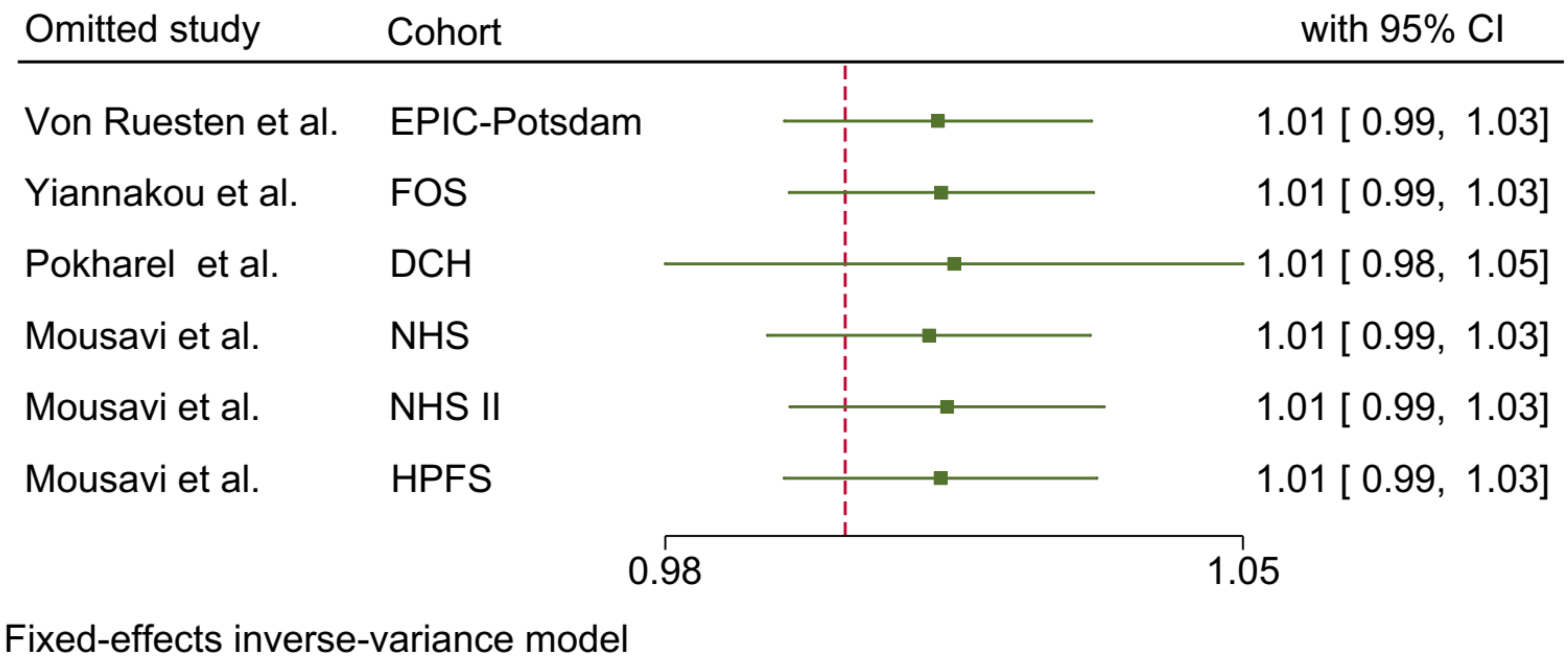

Supplementary Figure 13. Leave-one-out sensitivity analysis of the association between 3-serving/week **non-fried potato** consumption and risk of T2D.

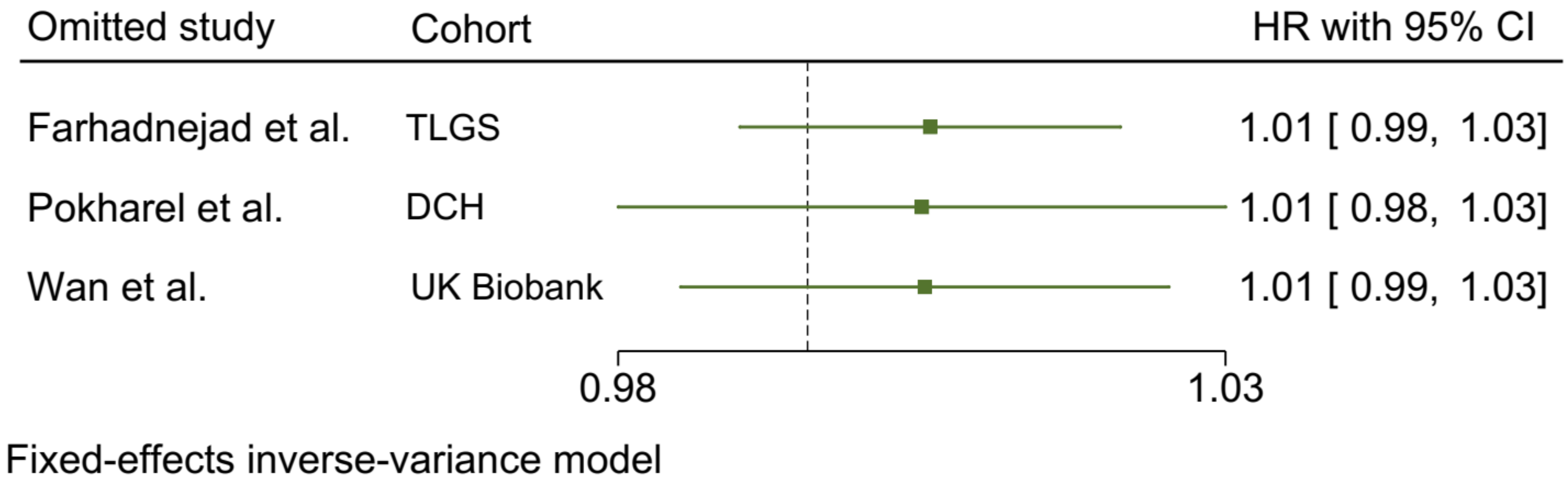

Supplementary Figure 14. Leave-one-out sensitivity analysis of the association between 3-serving/week **boiled potato** consumption and risk of T2D.

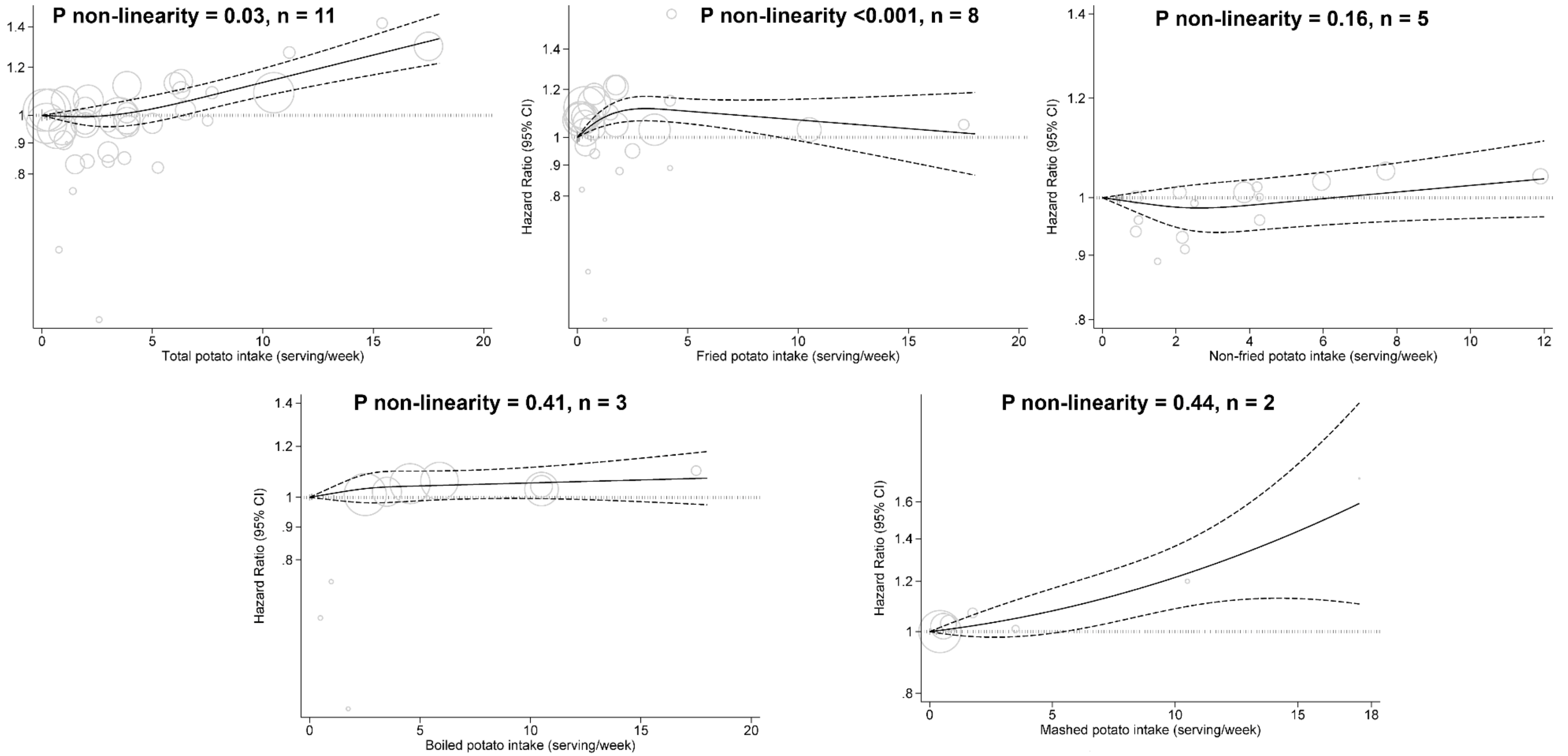

Supplementary Figure 15. Nonlinear dose-response association between potato consumption and risk of T2D. The solid line represents nonlinear dose-response, and dotted lines represent 95% CI. Circles represent relative risk point estimates for potato consumption categories from each study, with circle size proportional to the inverse of SE. Each study's baseline potatoes intake categories are indicated by small vertical black lines.

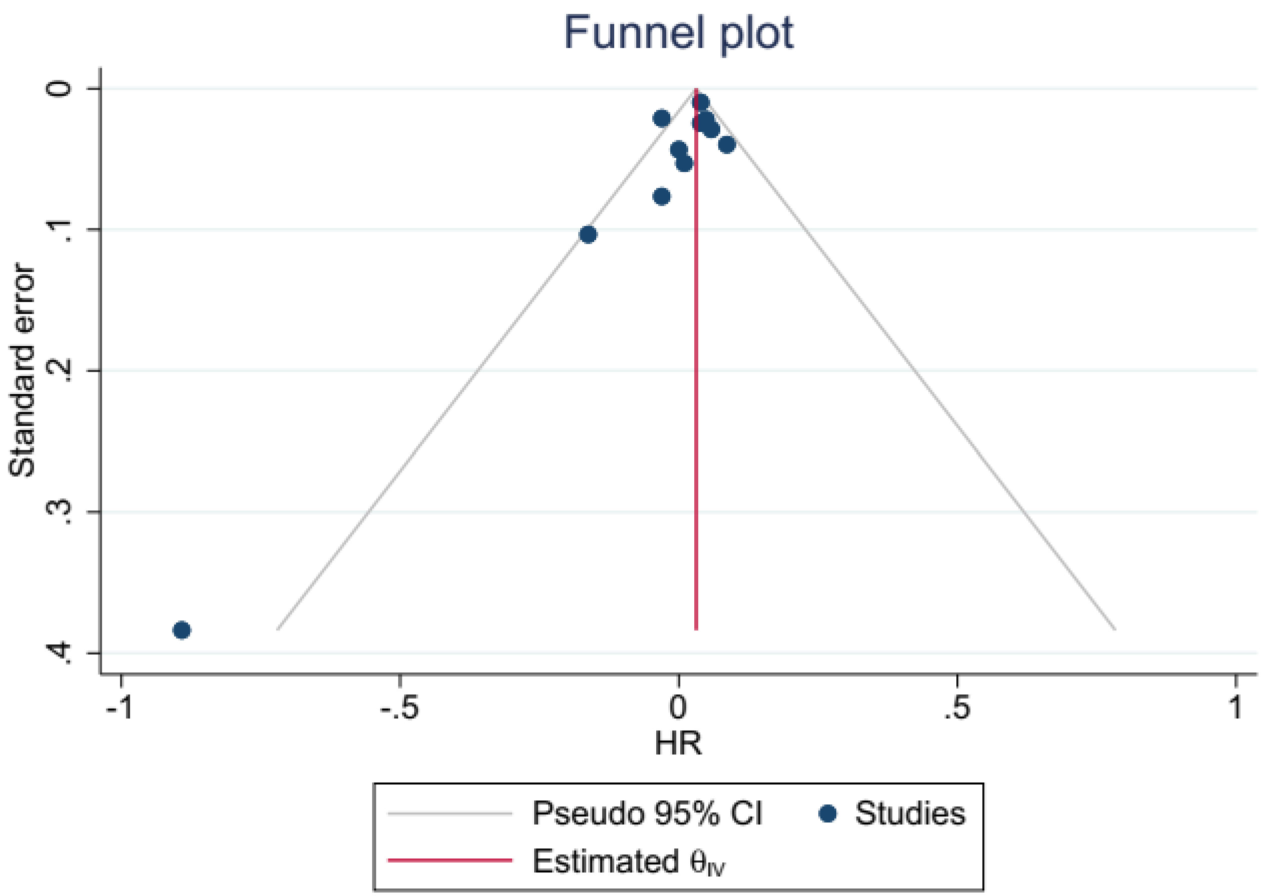

Supplementary Figure 16. Funnel plot for assessment of bias due to small studies for the association between 3-serving/week total potato consumption and risk of T2D.

P Egger's test = 0.13

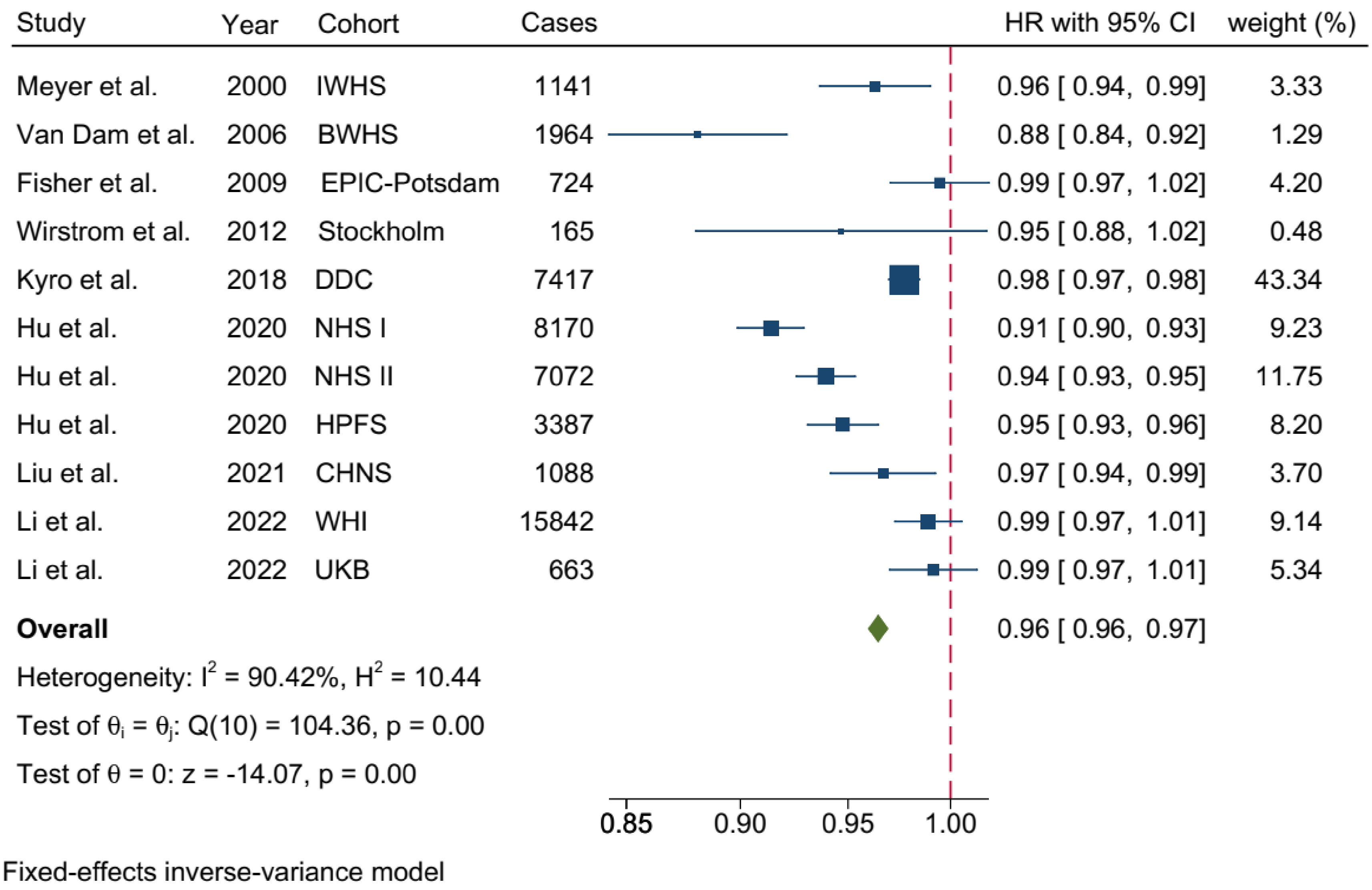

Supplementary Figure 17. Summary Hazard ratio of T2D incidence per 3 servings/week increase in whole grains consumption, using fixed effects model.

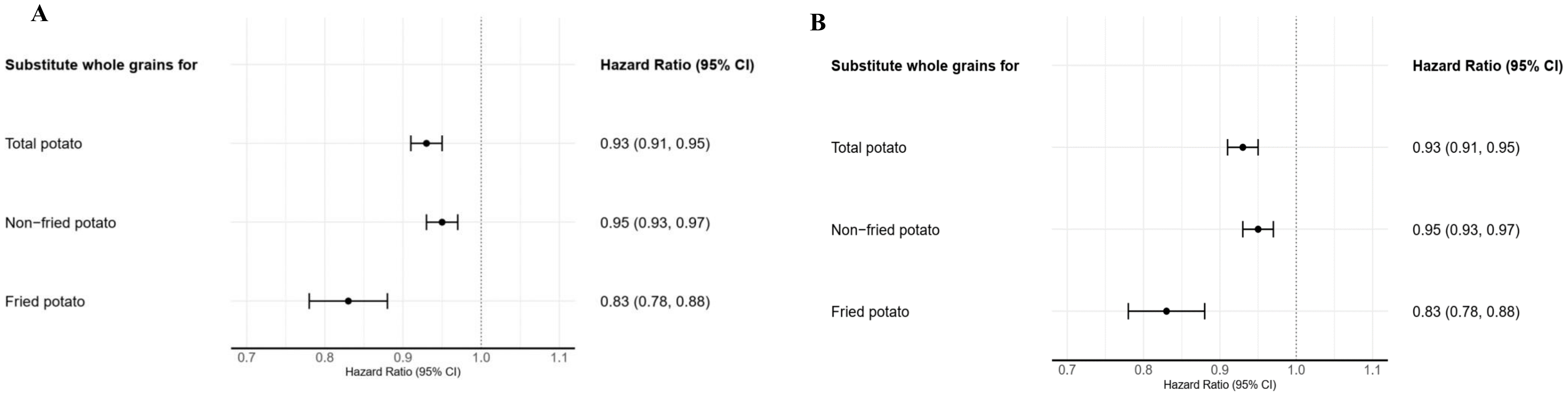

Supplementary Figure 18. The effect of replacing 3-serving/week of whole grain for different forms of potatoes on T2D incidence, using pooled data from meta-analyses of whole grains and potatoes, considering the correlations between whole grains and potatoes: A) 0.0; and B) 0.20.

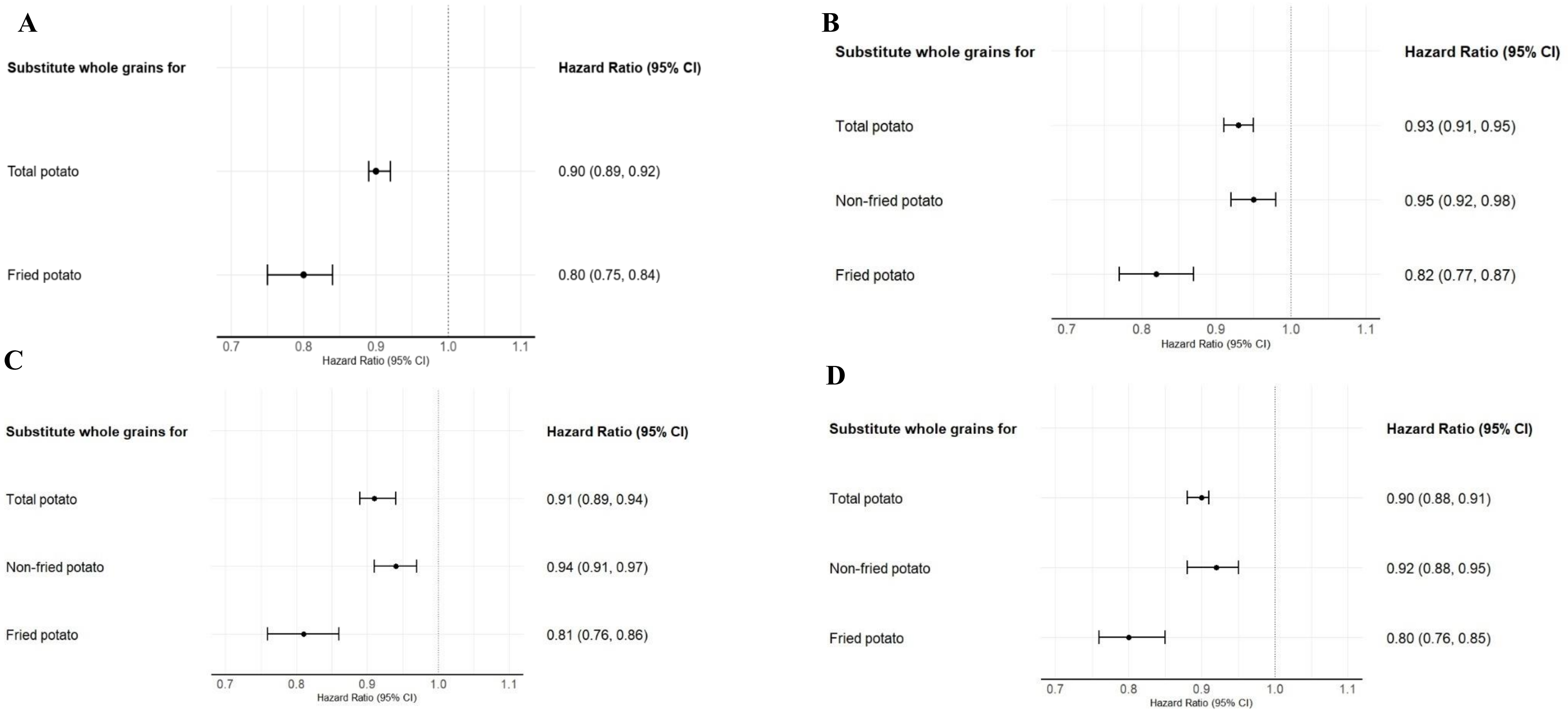

Supplementary Figure 19. The effect of replacing 3-serving/week of whole grain for different forms of potatoes on T2D incidence, using pooled data from meta-analyses of whole grains and potatoes: (A) limiting the analysis to the four overlapping cohorts (NHS, NHS II, HPFS, and UK Biobank); (B) studies with energy adjustment; (C) US-based studies; (D) studies with repeated dietary assessments.

Supplementary references:

1. Wells GA, Shea B, O'Connell D, et al. The Newcastle-Ottawa Scale (NOS) for assessing the quality of nonrandomised studies in meta-analyses. 2000
2. Higgins JP, Morgan RL, Rooney AA, et al. A tool to assess risk of bias in non-randomized follow-up studies of exposure effects (ROBINS-E). *Environ Int* 2024;186:108602.
3. Schünemann HJ, Cuello C, Akl EA, et al. GRADE guidelines: 18. How ROBINS-I and other tools to assess risk of bias in nonrandomized studies should be used to rate the certainty of a body of evidence. *Journal of clinical epidemiology* 2019;111:105-14.
4. Schwingshackl L, Knüppel S, Schwedhelm C, et al. Perspective: NutriGrade: a scoring system to assess and judge the meta-evidence of randomized controlled trials and cohort studies in nutrition research. *Advances in nutrition* 2016;7:994-1004.
5. Tobias DK, Wittenbecher C, Hu FB. Grading nutrition evidence: where to go from here? *Am J Clin Nutr* 2021;113:1385-87. doi: 10.1093/ajcn/nqab124 [published Online First: 2021/05/09]
6. Greenland S, Longnecker MP. Methods for trend estimation from summarized dose-response data, with applications to meta-analysis. *Am J Epidemiol* 1992;135:1301-09.
7. Orsini N, Bellocco R, Greenland S. Generalized least squares for trend estimation of summarized dose–response data. *The stata journal* 2006;6:40-57.
8. Harrell FE. Regression modeling strategies. *Bios* 2017;330:14.
9. Crippa A, Discacciati A, Bottai M, et al. One-stage dose–response meta-analysis for aggregated data. *Stat Methods Med Res* 2019;28:1579-96.
10. Orsini N, Li R, Wolk A, et al. Meta-analysis for linear and nonlinear dose-response relations: examples, an evaluation of approximations, and software. *American journal of epidemiology* 2012;175:66-73.
11. Rice K, Higgins JP, Lumley T. A re-evaluation of fixed effect (s) meta-analysis. *Journal of the Royal Statistical Society Series A: Statistics in Society* 2018;181:205-27.
12. DerSimonian R, Laird N. Meta-analysis in clinical trials. *Controlled clinical trials* 1986;7:177-88.
13. Chandler J, Cumpston M, Li T. Cochrane Handbook for Systematic Reviews of Interventions.
14. Higgins JP, Thompson SG. Quantifying heterogeneity in a meta-analysis. *Statistics in medicine* 2002;21:1539-58.
15. Deeks JJ, Bossuyt PM, Leeflang MM, et al. Cochrane handbook for systematic reviews of diagnostic test accuracy: John Wiley & Sons 2023.
16. Dunford EK, Miles DR, Popkin B, et al. Whole grain and refined grains: an examination of US household grocery store purchases. *The Journal of Nutrition* 2022;152:550-58.
17. Ghanbari-Gohari F, Mousavi SM, Esmaillzadeh A. Consumption of whole grains and risk of type 2 diabetes: A comprehensive systematic review and dose–response meta-analysis of prospective cohort studies. *Food Science & Nutrition* 2022;10:1950-60.
18. Bernstein AM, Sun Q, Hu FB, et al. Major dietary protein sources and risk of coronary heart disease in women. *Circulation* 2010;122:876-83.
19. Liu M, Liu C, Zhang Z, et al. Quantity and variety of food groups consumption and the risk of diabetes in adults: a prospective cohort study. *Clin Nutr* 2021;40:5710-17.

20. Li J, Glenn AJ, Yang Q, et al. Dietary protein sources, mediating biomarkers, and incidence of type 2 diabetes: findings from the Women's Health Initiative and the UK Biobank. *Diabetes Care* 2022;45:1742-53.
21. Egger M, Smith GD, Schneider M, et al. Bias in meta-analysis detected by a simple, graphical test. *bmj* 1997;315:629-34.
